# The Cultural Project: Formal Chronological Modelling of the Early and Middle Neolithic Sequence in Lower Alsace

**DOI:** 10.1007/s10816-016-9307-x

**Published:** 2017-01-09

**Authors:** Anthony Denaire, Philippe Lefranc, Joachim Wahl, Christopher Bronk Ramsey, Elaine Dunbar, Tomasz Goslar, Alex Bayliss, Nancy Beavan, Penny Bickle, Alasdair Whittle

**Affiliations:** 1 UMR 7044, Université de Strasbourg/ANTEA-Archéologie, 11 rue de Zurich, 68400 Habsheim, France; 2 UMR 7044, Université de Strasbourg/INRAP, 10 rue d’Altkirch, 6700 Strasbourg, France; 3 State Office for Cultural Heritage Management Baden-Württemberg, Osteology, Stromeyersdorfstraße 3, 78467 Constance, Germany; 4 Oxford Radiocarbon Accelerator Unit, Research Laboratory for Archaeology and the History of Art, Dyson Perrins Building, University of Oxford, Oxford, OX1 3QY UK; 5 SUERC Radiocarbon Dating Laboratory, Scottish Enterprise Technology Park, Rankine Avenue, East Kilbride, G75 0QF UK; 6 Poznań Radiocarbon Laboratory, Adam Mickiewicz University, Rubież 46, 61-612 Poznań, Poland; 7 Scientific Dating, Historic England, 1 Waterhouse Square, 138-42, Holborn, London EC1N 2ST UK; 8 University of Stirling, FK9 4LA Stirling, UK; 9 Department of Archaeology and Conservation, Cardiff University, John Percival Building, Colum Drive, Cardiff, CF10 3EU UK; 10 Department of Archaeology, University of York, King’s Manor, York, Yo1 7EP UK

**Keywords:** Neolithic, Lower Alsace, Formal chronological modelling, Cultural diversity, Continuity and discontinuity

## Abstract

Starting from questions about the nature of cultural diversity, this paper examines the pace and tempo of change and the relative importance of continuity and discontinuity. To unravel the cultural project of the past, we apply chronological modelling of radiocarbon dates within a Bayesian statistical framework, to interrogate the Neolithic cultural sequence in Lower Alsace, in the upper Rhine valley, in broad terms from the later sixth to the end of the fifth millennium cal BC. Detailed formal estimates are provided for the long succession of cultural groups, from the early Neolithic Linear Pottery culture (LBK) to the Bischheim Occidental du Rhin Supérieur (BORS) groups at the end of the Middle Neolithic, using seriation and typology of pottery as the starting point in modelling. The rate of ceramic change, as well as frequent shifts in the nature, location and density of settlements, are documented in detail, down to lifetime and generational timescales. This reveals a Neolithic world in Lower Alsace busy with comings and goings, tinkerings and adjustments, and relocations and realignments. A significant hiatus is identified between the end of the LBK and the start of the Hinkelstein group, in the early part of the fifth millennium cal BC. On the basis of modelling of existing dates for other parts of the Rhineland, this appears to be a wider phenomenon, and possible explanations are discussed; full reoccupation of the landscape is only seen in the Grossgartach phase. Radical shifts are also proposed at the end of the Middle Neolithic.

**Electronic supplementary material:**

The online version of this article (doi:10.1007/s10816-016-9307-x) contains supplementary material, which is available to authorized users.

## The Cultural Project: Variations in Time and Space

In asking why there is so much diversity in human social and cultural life, at any one time, down the generations and across space, archaeologists often look to anthropology to explore the myriad possibilities. In charting his disciplines’s shift from seeing diversity as a ‘great arc’ of bounded entities, to regarding it as a symptom of a much more fluid and interconnected world, the anthropologist Michael Carrithers argued that we should think of human life as formed by a system or systems of relationships, and as metamorphic, causal and interactive (Carrithers 1992, pp. 27 and 31); he has sketched ‘a setting in which change might be thought of as natural, the setting of actual social life with all its fluidity, uncertainty, construals and misconstruals, its laboriously achieved continuity, its planned and inadvertent innovations’ (Carrithers 1992, p. 36). What he has also called the culture project involves ‘the finding and displaying of variations in the cultural rhetorical resources which people use on themselves and one another to establish a scene, make a movement, and lead to a performance’ (Carrithers 2010, p. 167). He is by no means alone in characterising human diversity thus, but not everyone has agreed. Despite studies which have sought to map the subtleties, inter-penetrations and boundaries of variation (e.g. Barth 1969; Knauft 1993), the search for order and pattern has been dismissed, with a counter-appeal to ‘an unbroken landscape of variation’ (Ingold 1996, p. 117), which may fit into a distinctively ‘British approach’ in which the ultimate aim is to unpick the claim for unified culture (Kuper 1999). However, and importantly, there is also the problem of the timescales of anthropology and ethnographic observation, in which the convenient fiction of the ‘ethnographic present’ looms too large (Fabian 1983).

This can be contrasted with the practice of archaeology, which, classically and distinctively, is in possession of long timescales for ordering the diversity of material culture, which is every bit as varied as that described above with reference to anthropology. But how that greater sense of time depth has been constructed and regarded in archaeology, especially in prehistory, in turn leaves much to be desired. There have been various approaches. One familiar amalgam is witnessed in the near-ubiquitous use of charts of relative material culture sequences, arranged into neat blocks of time, more often than not divided up by straight horizontal lines, each successive brick in the edifice resting squarely on its predecessor, and often encompassing a couple of centuries—or more (Schier 2014).

These kinds of sequences often derive from typological series, which attempt to capture the linkages or drift between categories of finds (Adams and Adams 2008). More formal ordination can be derived from the statistical analysis of the occurrence of types in units, often using correspondence analysis or multidimensional scaling (Baxter 1994). These regional relative chronological series are joined through comparison of types and contact finds, and a calendrical timescale then applied to the working structure using tree-ring dates if to hand (not normally the case) and by visual inspection of the available assemblage of radiocarbon dates, usually measured on less than perfectly selected samples.

The seeming solidity of such chronological constructions brings interpretive consequences. In many cases, there is an acceptance of working with considerable and often undifferentiated blocks of time, although, in situations where change in material culture is swift, seriation can reveal the relative sequence in detail. These formal statistical methods demonstrate continuity in the types and units analysed, but seriation of itself does not produce either timescale or tempo. Where formal seriation has not been undertaken, or is not possible, there is also a default tendency to assume continuity: unbroken and smooth transition from one cultural phase to another, with a steady and even pace of change.

So from the outset, approaching the diversity of human life and culture, we can identify major issues in understanding the pace and tempo of change and major questions about the potential for continuity and discontinuity. One aspect which the chronological charts of archaeology are good at is tracking regional variation over the long term but, given the lack of precision and difficulties in dating many of the existing schemes, currently this variation is usually only visible at rather coarse scales of resolution. To unravel the cultural project of the past in much more detail requires much more effective means of observing and dating diversity and change.

Applying a Bayesian methodological approach to an archaeological chronology, we examine here a sequence where the default position has been to assume both a more or less regular pace of turnover and continuity.

## The Neolithic Archaeology of the Southern Part of the Upper Rhine Valley

**Fig. 1:**
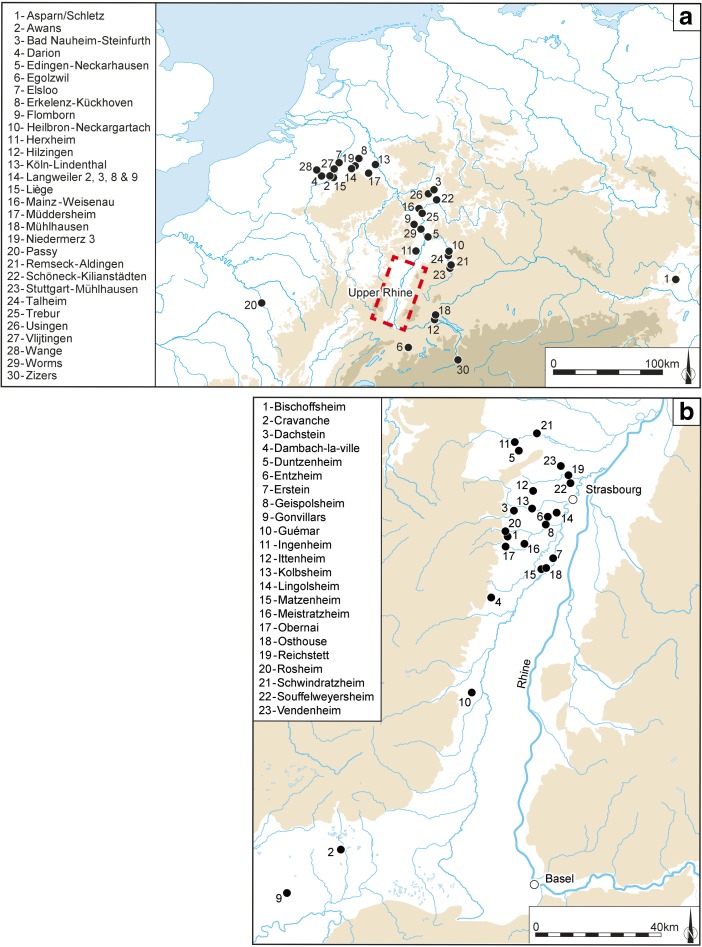
Map of **a** the Rhineland and surrounding regions and **b** the upper Rhine valley, showing sites mentioned in the text

To explore these issues in greater depth, within the context of a dating and formal modelling project on the Neolithic of Europe (see ‘Acknowledgements’), the Early and Middle Neolithic sequence of the southern part of the upper Rhine valley was chosen as a case study (Fig. 1). The region under study lies on the west side of the upper Rhine, in Lower Alsace (in France), approximately some 55 km from south to north and some 25 km from river to flanking uplands of the Vosges. There is also a narrow strip on the east side of the Rhine (in Germany), before the uplands of the Black Forest, from where comparable finds have been made, especially around the Kaiserstuhl and to its south. Much of the rich Neolithic archaeology of the region comes from rescue and contract excavations on the loess-covered terraces of the valley, though an older tradition of research investigations can be tracked back to the late nineteenth and beginning of the twentieth century (Lefranc 2007; Denaire 2009).

**Fig. 2:**
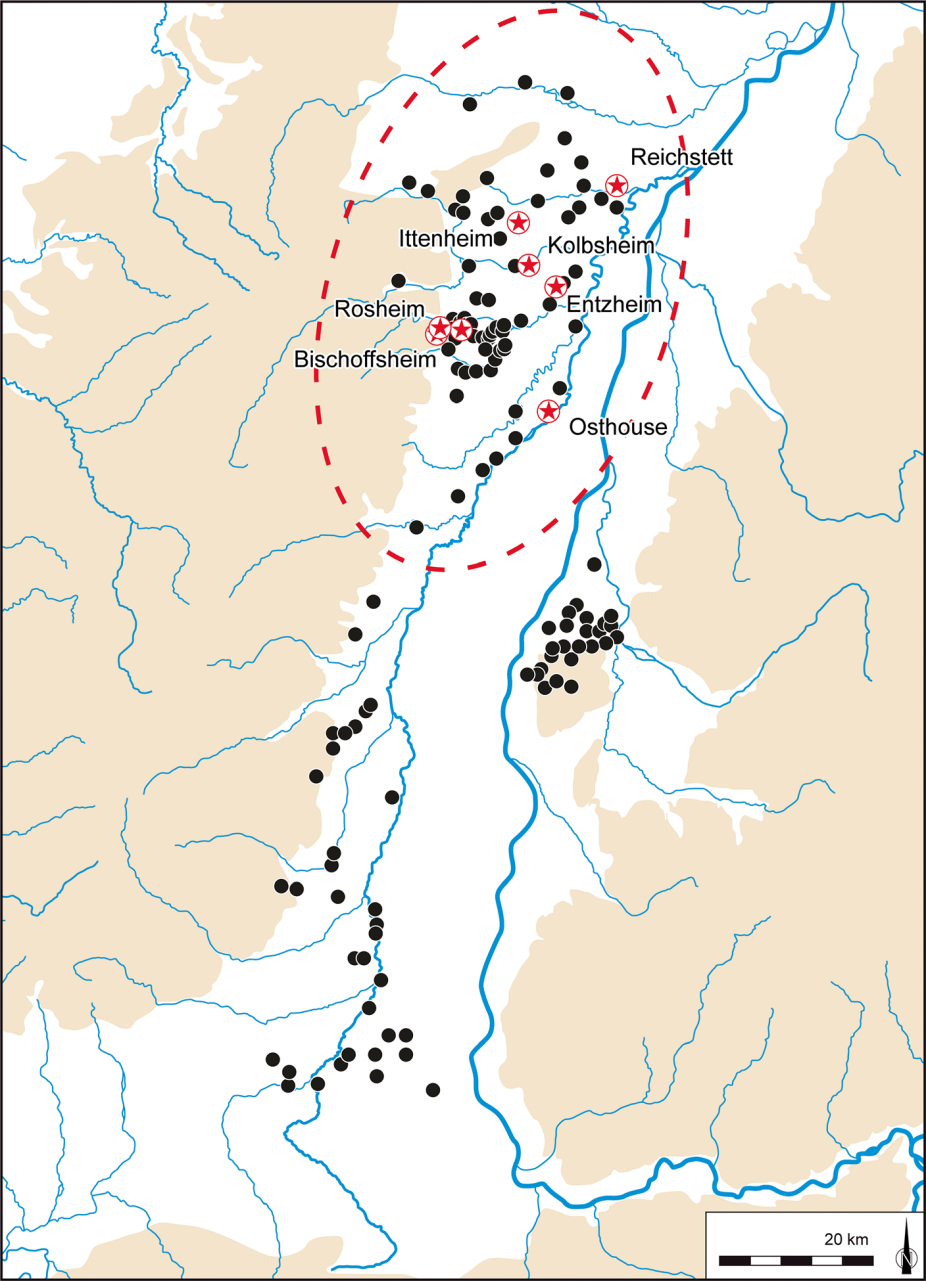
Map of the upper Rhine valley, showing the distribution of LBK settlements and sites which provided radiocarbon samples. Lower Alsace is *circled by the red dotted line*

**Fig. 3:**
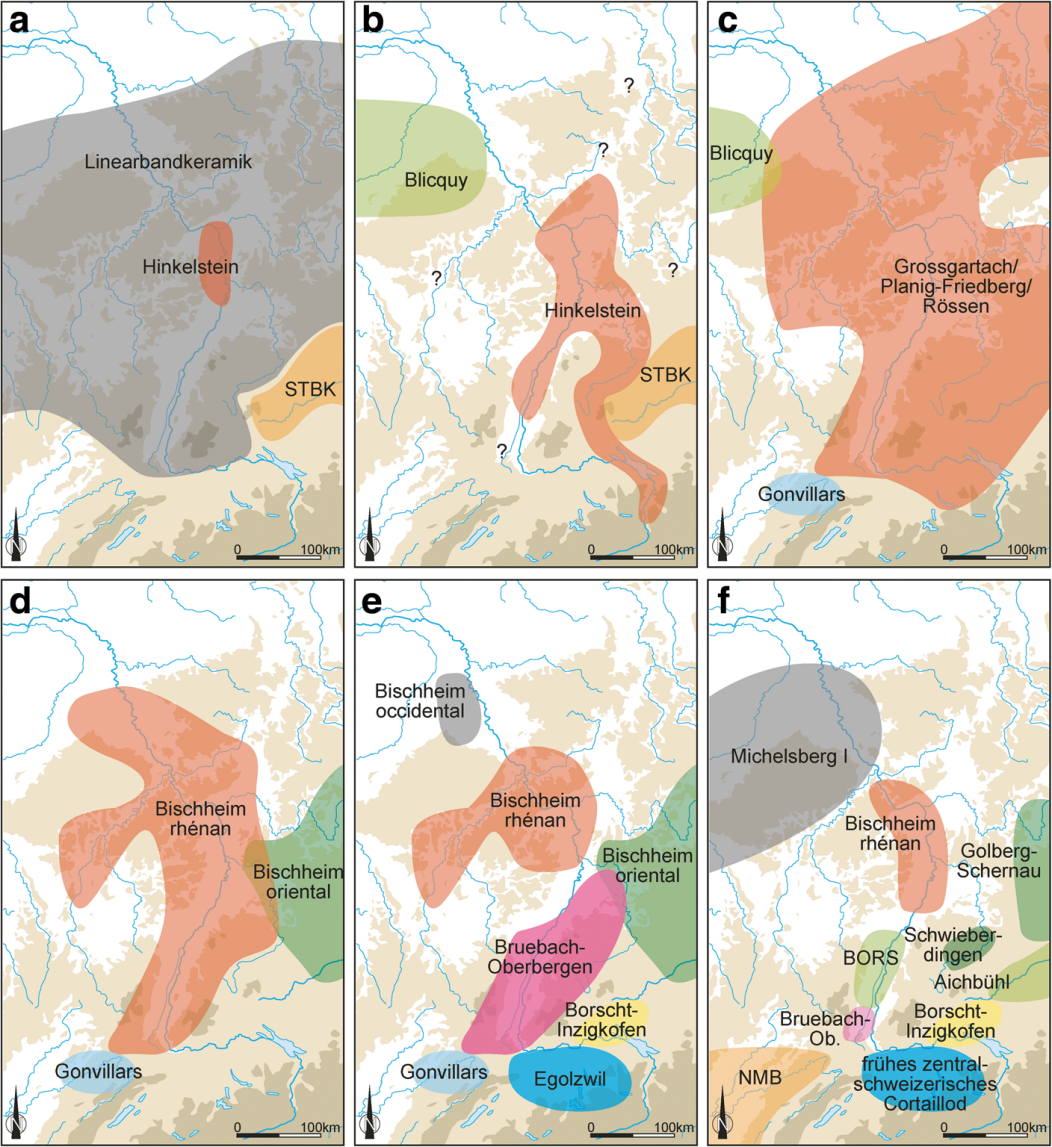
Maps of the upper Rhine valley and surrounding regions, showing the maximum spatial extent of different styles of Early and Middle Neolithic ceramics (related pottery groups are shown only where undisputed contact finds demonstrate at least partial contemporaneity): **a** LBK, **b** Hinkelstein, **c** Grossgartach, Planig-Friedberg and Rössen, **d** Bischeim, **e** Bruebach-Oberbergen and **f** BORS (NMB: Néolithique moyen Bourguignon)

**Fig. 4:**
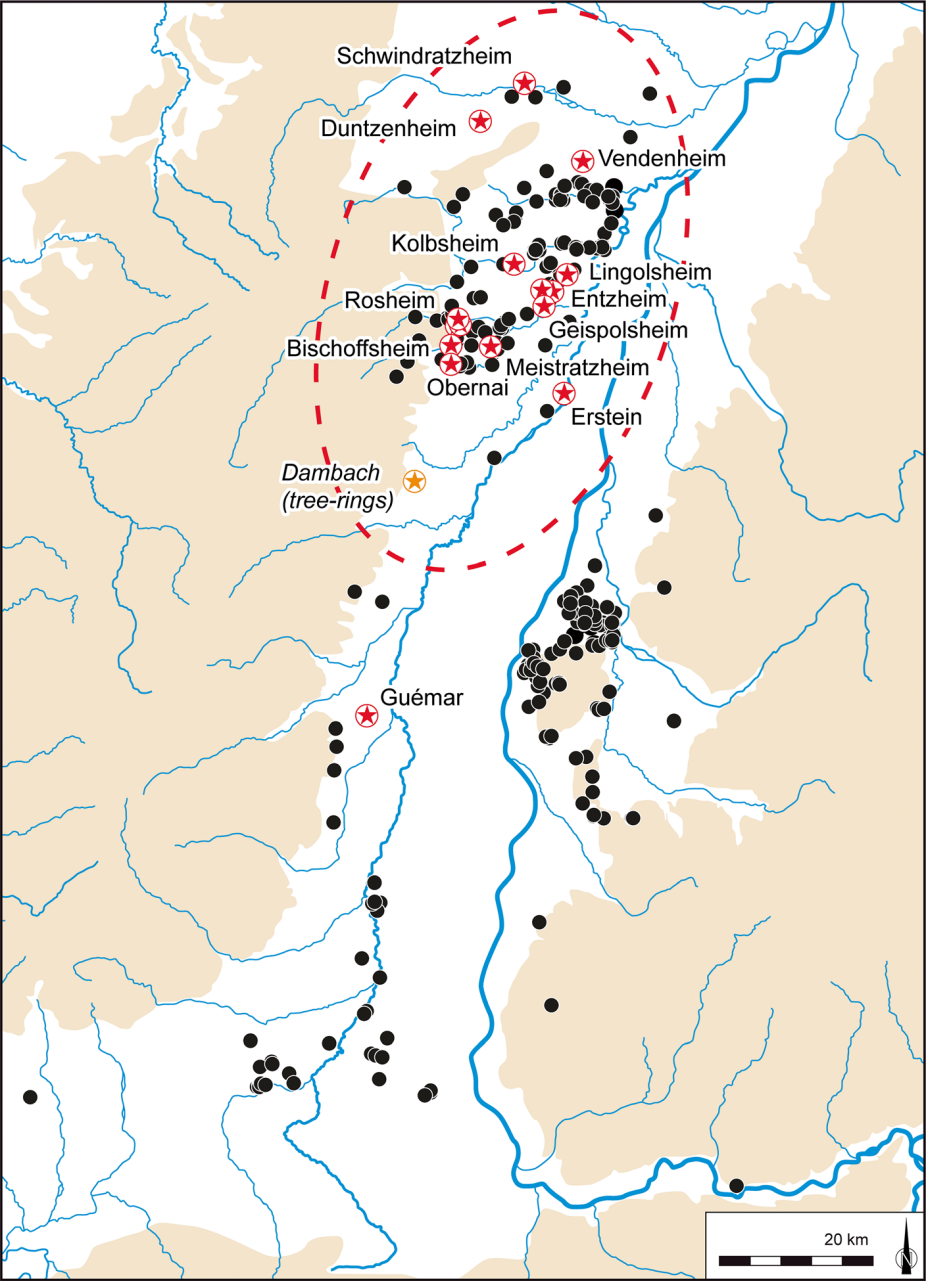
Map of the upper Rhine valley, showing the distribution of Middle Neolithic settlements and sites which provided radiocarbon samples. Lower Alsace is *circled by the red dotted line*

In broad terms, the Early Neolithic sequence begins in the latter part of the sixth millennium cal BC, with the appearance and development of the *Rubané* culture (Linear Pottery culture (LBK); Figs 2 and 3a). There then follows the Middle Neolithic sequence, covering much of the fifth millennium cal BC, from the Hinkelstein, Grossgartach, Planig-Friedberg and Rössen phases, on to the Bischheim, Bruebach-Oberbergen and *Bischheim Occidental du Rhin Supérieur* (BORS) 0–II phases (Figs. 3b–f and 4). Beyond the detail, the sequence represents the appearance of a Neolithic way of life, presumably derived from central Europe (Bickle and Whittle 2013, with references), and its subsequent development. From the end of the fifth millennium cal BC, a rather different scene emerged, with a series of contrasts to this initial ‘Danubian’ world.

Up till now, the absolute dating of these successive cultural phases in Lower Alsace and, more widely, across the upper Rhine valley as a whole, has been based on a small number of radiocarbon dates. Moreover, a number of them, in particular those from the end of fifth millennium, were only indirectly fixed in time, through comparison with the better-dated sequences in neighbouring regions such as the Alpine foreland. Despite these substantial limitations, the arrival of the early Neolithic (the LBK) in the Rhineland was previously estimated at about 5300 cal BC and its end placed a little after 5000 cal BC, based on a dendrochronological date from Erkelenz-Kückhoven much further down the Rhine (Lefranc 2007; Denaire 2009, 2011). The beginning of the Hinkelstein phase (and the Middle Neolithic) was thought to overlap with the end of the LBK, at least about 4950 cal BC, and from those, developed the Grossgartach phase. This culture, with the Planig-Friedberg group, was seen to persist until after 4750 cal BC, and to be replaced by the Rössen culture, itself succeeded about 4450 cal BC at the latest by the Bischheim group. That in turn evolved locally into the Bruebach-Oberbergen group about 4300 cal BC, which was supplanted by the BORS group a little after 4200 BC cal BC. The arrival of the Michelsberg culture, about 4000 cal BC, marked the end of the BORS and the Middle Neolithic sequence in Lower Alsace.

## Aims and Approach of This Study

This dating project aimed to demonstrate the potential for a carefully selected series of radiocarbon dates to be combined with ceramic sequences derived from seriation, to produce refined chronologies for the Neolithic period in situations where vertical stratigraphic sequences are limited. We thus aim to counter by practical demonstration the, in our view erroneous and widespread, belief that precise chronologies can only be obtained by Bayesian chronological modelling of radiocarbon dates in situations where we have deep archaeological stratigraphies.

It exploits methodology developed in a study of Anglo-Saxon graves and grave-goods (Bayliss et al. 2013) but aims to provide a similar generational chronology for prehistory. The Anglo-Saxon study was deliberately designed to combine the relative dating provided by seriation of artefact-types in grave assemblages with a suite of high-precision radiocarbon dates in formal Bayesian chronological models. The aim was to produce accurate date estimates precise to within the few decades that are required to be useful in archaeological interpretation in a period and a place that is on the margins of written history. To this end, all elements of the analysis were optimised for chronological sensitivity and precision. A reflexive and interactive approach was taken to the construction (and re-construction) of artefact typologies, seriation of the artefact types and grave assemblages by correspondence analysis, and the radiocarbon dating programme. This ensured that both the typologies and seriation were optimised to reveal chronological variation and that the sample of radiocarbon-dated graves was representative of the seriation. The high-precision and accuracy demanded for the Anglo-Saxon study also meant that technical aspects of radiocarbon dating that are of little practical relevance in most applications had to be considered. This led to re-measurement of the relevant portion of the radiocarbon calibration curve (McCormac et al. 2004, 2008) and detailed consideration of the potential for diet-derived radiocarbon reservoir effects in the dated individuals (Mays and Beavan 2012; Bayliss et al. 2013, Chap. 4), laboratory reproducibility (McCormac et al. 2001, 2011), sample diagenesis (Beavan et al. 2011) and even the turnover time of collagen in human bone (Barta and Stolc 2007; Hedges et al. 2007).

In this study, we wished to transfer these careful methodologies to the prehistoric period, applying them not only to well-established existing seriations, but also to material that did not fall on a particularly favourable part of the calibration curve (the Anglo-Saxon project had deliberately targeted the steep part of the curve in the seventh century AD) and which could only be dated by accelerator mass spectrometry. The southern part of the upper Rhine valley was chosen as our case study because in this small and coherent area formal seriations for the LBK and Middle Neolithic Hinkelstein–Rössen assemblages were already available, based on the presence of decorative motifs on pots from pits and graves (Lefranc 2007; Denaire 2009). The clear parabolae obtained from the correspondence analyses in these studies demonstrate that pottery in the pits and graves concerned can be considered as closed assemblages (Greenacre 1984, 1993). Critically, bone preservation in this region is generally sufficiently good to allow radiocarbon dating. This enabled sufficient samples of articulated or articulating bone, which must be close in age to their parent contexts, to be dated. Occasionally, charred food crusts or pitch used to repair vessels could also be dated. The association between the dated material and the closed context included in the correspondence analysis is thus watertight.

In this way, we aimed to obtain precise chronologies for the development of material culture over the long term of this regional sequence. From these, we hoped to derive a much more detailed understanding of diversity in general, and of the pace and tempo of change in particular, if possible down to a generational timescale, and to interrogate existing assumptions about continuity over the long term. Finally, we intended to compare results from the small case study area with existing dates from surrounding areas, and thus to place the regional ‘cultural project’ in a wider context.

## Radiocarbon Dating and Chronological Modelling

The radiocarbon dating programme for the ceramic sequence in Lower Alsace was conceived within the framework of Bayesian chronological modelling (Buck et al. 1996). Such an approach allows the combination of archaeological information from the ceramic seriations with calibrated radiocarbon dates using a formal statistical methodology.

In this case study, we use the relative sequence derived from correspondence analyses of the decorative motifs on pottery assemblages from closed contexts (graves or pits) as informative prior information in our Bayesian chronological models. We therefore assume that this ordering is statistically independent of the radiocarbon content of the dated samples. This is patently not the case, since both are clearly related to earth-based chronology. In this study, however, we wished to build on the solid foundation of typology and material sequences established by past generations of researchers. The seriations employed in this study were constructed without reference to the radiocarbon dates, so our Bayesian modelling effectively combined two separate strands of information both of which relate to the calendar dating of the features and assemblages included in our analyses.

For our models to be valid, however, it is essential that the relative sequence of contexts derived from the seriation is the same as the relative sequence of the radiocarbon dates. First, we consider the archaeological association between the dated samples and the closed contexts included in the seriation; then we consider the measurement and reproducibility of the scientific measurements obtained. Issues of calibration are beyond the scope of this paper. Although we note that there may be additional structure in the changing concentration of atmospheric radiocarbon that is not revealed by the data presently available (e.g. Kromer et al. 2010; Taylor and Southon 2013), the use of a common standard in the form of an internationally agreed calibration dataset (IntCal13; Reimer et al. 2013) means that our chronologies are comparable with others based on radiocarbon dating. As only one mature individual was dated (a woman from grave 0010 at Schwindratzheim ‘Les Terrasses de la Zorn’ associated with Bischheim pottery; Table 2), we do not consider the impact of bone turnover on this study (cf Bayliss et al. 2013, Fig. 2.23). The potential impact of diet-derived radiocarbon reservoir effects in measurements on human bone are explored in the sensitivity analysis described below.

### Sampling

As the identification of closed assemblages and chronologically sensitive traits is key to successful seriation, the identification of samples for radiocarbon dating, which are demonstrably not residual in the contexts from which they were recovered, is essential if the sequence of those contexts provided by seriation is to be used to constrain the calibration of the radiocarbon dates. First, all potential samples must be of short-lived material. Secondly, they must be contemporary with their parent contexts. Assessing whether material is residual is the most hazardous step in sample selection, since the taphonomic relationship between a sample and its context is always a matter of interpretative judgement rather than certain knowledge. We employed the hierarchy of criteria for assessing sample taphonomy outlined by Bayliss et al. (2011, pp. 38–42), selecting samples of:

- Bones found in articulation, which would have been still connected by soft tissue when buried and hence from recently dead individuals;
- Bones identified as articulating during analysis, which may have been articulated in the ground or have only been slightly disturbed before burial;
- Bones with refitting unfused epiphyses identified during analysis, for the reasons given above;
- Bones where the refitting unfused epiphysis (although lost) is judged to have been present in the ground on the basis of the colour and preservation of the epiphyseal surface;
- Carbonised food residues relating to the final use of pottery sherds, which are thought to be from closed contexts because of the results of the correspondence analysis;
- Pitch used to repair pottery, on sherds that are similarly judged to be from closed contexts;
- Pairs of bones judged on osteological grounds to be from the same individual.

Selecting samples on this basis minimises the archaeological risk that the dated sample may not have a direct association with the time when the ceramic assemblage included in the correspondence analysis was deposited. Bone and residues on pottery are not, however, the easiest materials to date accurately using radiocarbon (Bayliss et al. 2011, pp. 45–57), and so concentrating on these materials adds scientific risk to the dating programme. This risk was managed by obtaining replicate measurements from two laboratories on a significant number of samples (see below), through a programme of stable isotopes to investigate the potential for dietary offsets in the samples of human bone, and through statistical approaches which measure the compatibility of the radiocarbon dates with the sequences produced by the correspondence analyses.

A sequential sampling strategy was adopted (Bayliss 2009), with a skeleton set of five or six samples submitted from each phase of the LBK and Middle Neolithic seriations. This led to the large number of samples submitted from the Grossgartach phase, as the initial seriation suggested that this might be sub-divided into five sub-phases (of which four could be sampled). Initial results led to an intensive search for datable material from features that contained the latest LBK and Hinkelstein pottery. A small number of additional samples were also submitted from selected other phases where simulation suggested these would refine the interim chronology and/or make it more representative. Sampling was also extended to include the typological succession of the later Middle Neolithic Bischheim/BORS stages. A final round of samples increased the sample of Hinkelstein dates by sampling three burials from the cemetery Remseck-Aldingen ‘Halden’ in the neighbouring Neckar valley.

### Radiocarbon Dating

The details of the radiocarbon dates used in the chronological models for the LBK and Middle Neolithic ceramic sequences in Lower Alsace are given in Tables 1 and 2. One hundred and fifteen measurements were obtained as part of this study, with a further 37 inherited from previous research. All are conventional radiocarbon ages (Stuiver and Polach 1977), except for the three results produced by the Lyon laboratory (Ly-) in the 1970s which were not corrected for fractionation. Details of methods used for their processing and dating are provided in the Electronic Supplementary Material.

**Table 1:** Radiocarbon and stable isotopic measurements for the LBK ceramic sequence in Lower Alsace (reported stable isotope values were measured by isotopic ratio mass spectrometry)

Laboratory number	Sample/find number	Material, stratigraphic details and site	Radiocarbon age (BP)	δ^13^C (‰)	δ^15^N (‰)	C/N	References
LBK IIb							
Ly-865	Reich.43	Fossil pitch mixed with earth from pit 43, Reichstett	5940 ± 150				Evin *et al*. (1976)
OxA-27767	TOTL_RL_032	Cattle, articulated right 1st phalanx and metacarpal from pit 152, Kolbsheim ‘Vogeseblick’	6211 ± 34	−22.4 ± 0.2	9.1 ± 0.3	3.1	Denaire (2013)
SUERC-47710	TOTL_RL_062	Cattle calcaneum articulating with astragalus, from pit 1413, Bischoffsheim ‘AFUA du Stade’	6234 ± 30	−21.1 ± 0.2	8.4 ± 0.3	3.2	Lefranc *et al*. (2004)
OxA-29695	TOTL_RL_068	Residue, pitch/food crust, on sherd which is part of seriation from pit 264, square A.14, Bischoffsheim ‘AFUA du Stade’	6225 ± 40	−24.6 ± 0.2			Lefranc *et al*. (2004)
SUERC-55324	TOTL_RL_092	Pig lumbar vertebra refitting with unfused epiphysis, from pit 696, Bischoffsheim ‘AFUA du Stade’	6207 ± 34	−21.4 ± 0.2	6.5 ± 0.3	3.2	Lefranc *et al*. (2004)
OxA-30785	TOTL_RL_093	Pig right tibia articulating with other foot bones, from pit 87A, Osthouse ‘Kleinfeld’	6227 ± 39	−21.2 ± 0.2	7.3 ± 0.3	3.2	Perrin (2013)
SUERC-55325	TOTL_RL_094	Pig, articulating left 3rd and 4th metacarpals from pit 87B, Osthouse ‘Kleinfeld’	6175 ± 34	−20.8 ± 0.2	7.1 ± 0.3	3.2	Perrin (2013)
LBK IIc							
SUERC-46497	TOTL_RL_033	Pig, two pieces of refitting unfused atlas, from pit 215, Bischoffsheim ‘AFUA de Stade’	6288 ± 34	−21.1 ± 0.2	8.9 ± 0.3	3.3	Lefranc *et al*. (2004)
OxA-27805	TOTL_RL_034	*Cervus elaphus*, articulating calcaneum and astragalus from pit 430, Bischoffsheim ‘AFUA du Stade’	6281 ± 31	−21.3 ± 0.2	5.3 ± 0.3	3.3	Lefranc *et al*. (2004)
OxA-28981	TOTL_RL_063	*Capra hircus*, horn core, both horns present and articulating with part of unfused skull, from pit 1, Bischoffsheim ‘AFUA du Stade’	6209 ± 33	−20.2 ± 0.2	9.2 ± 0.3	3.2	Lefranc *et al*. (2004)
SUERC-47711	TOTL_RL_064	Pig, distal unfused metacarpal, epiphysis missing but judged to have been present when the bone entered the ground, from pit 1961, Bischoffsheim ‘AFUA du Stade’	6206 ± 33	−21.4 ± 0.2	7.3 ± 0.3	3.3	Lefranc *et al*. (2004)
OxA-28898	TOTL_RL_065	Cattle vertebra with refitting unfused epiphysis, from pit 693, Kolbsheim ‘Vogeseblick’	6237 ± 32	−22.06 ± 0.2	7.3 ± 0.3	3.3	Denaire (2013)
LBK III							
OxA-27766	TOTL_RL_030 (a)	*Capra hircus* articulated radius and ulna from pit 107, Kolbsheim ‘Vogeseblick’	6147 ± 33	−19.9 ± 0.2	7.8 ± 0.3	3.2	Denaire (2013)
OxA-27804	TOTL_RL_030 (a)	Replicate of OxA-27766	6193 ± 31	−19.7 ± 0.2	7.7 ± 0.3	3.1	
SUERC-46451	TOTL_RL_030 (b)	Replicate of OxA-27766	6107 ± 33	−20.1 ± 0.2	7.7 ± 0.3	3.3	Denaire (2013)
6151 ± 19 BP, *T′* = 3.6; −19.9 ± 0.12‰, *T′* = 2.0; +7.7 ± 0.17‰, *T′* = 0.1 (*T′* (5%) = 6.0, *ν* = 2 for all)							
OxA-27768	TOTL_RL_035	*Ovis aries* metacarpal with refitting unfused epiphysis, from pit 138, Bischoffsheim ‘AFUA du Stade’	6205 ± 32	−21.2 ± 0.2	6.9 ± 0.3	3.3	Lefranc *et al*. (2004)
SUERC-46452	TOTL_RL_037	Sheep/goat, articulating tibia and astragalus from pit 1682, Bischoffsheim ‘AFUA du Stade’	6192 ± 33	−21.4 ± 0.2	9.1 ± 0.3	3.2	Lefranc *et al*. (2004)
OxA-27769	TOTL_RL_038 (a)	Cattle 1st phalanx, articulating with epiphysis of metatarsal/carpal, from pit 447, Kolbsheim ‘Vogeseblick’	6178 ± 31	−21.8 ± 0.2	8.8 ± 0.3	3.2	Denaire (2013)
SUERC-46453	TOTL_RL_038 (b)	Replicate of OxA-27769	6147 ± 29	−21.8 ± 0.2	8.8 ± 0.3	3.3	Denaire (2013)
6162 ± 22 BP, *T′* = 0.5; −21.8 ± 0.14‰, *T′* = 0.0; +8.8 ± 0.21‰, *T′* = 0.0 (*T′* (5%) = 3.8, *ν* = 1 for all)							
OxA-28899	TOTL_RL_066	Sheep/goat vertebra with refitting unfused epiphysis, from pit 447, Kolbsheim ‘Vogeseblick’	6238 ± 32	−20.74 ± 0.2	8.3 ± 0.3	3.3	Denaire (2013)
OxA-X-2555-56	TOTL_RL_070	Residue, pitch/food crust, on sherd which is part of seriation from pit 138, square A.20, Bischoffsheim ‘AFUA du Stade’	6270 ± 90	−27.6 ± 0.2			Lefranc *et al*. (2004)
OxA-29630	TOTL_RL_071	Residue, pitch/food crust, on sherd which is part of seriation from pit 138, square B.15, Bischoffsheim ‘AFUA du Stade’	6219 ± 33	−28.3 ± 0.2			Lefranc *et al*. (2004)
OxA-30786	TOTL_RL_095	Cattle, articulating calcaneum and astragalus, from pit 1403, square 1, Bischoffsheim ‘AFUA du Stade’	6252 ± 38	−23.5 ± 0.2	7.4 ± 0.3	3.1	Lefranc *et al*. (2004)
SUERC-55326	TOTL_RL_096	Pig humerus with refitting unfused epiphysis, from pit 1403, square 5, Bischoffsheim ‘AFUA de Stade’	6224 ± 34	−21.1 ± 0.2	7.6 ± 0.3	3.2	Lefranc *et al*. (2004)
LBK IVa							
SUERC-46502	TOTL_RL_049	Cattle humerus with refitting unfused epiphysis, from pit 450, Kolbsheim ‘Vogeseblick’	6156 ± 34	−22.8 ± 0.2	9.1 ± 0.3	3.2	Denaire (2013)
LBK IVa1							
OxA-27770	TOTL_RL_040	*Cervus elaphus,* vertebra with partially fused epiphysis articulating with unfused epiphysis of the neighbouring vertebra, from pit 686, Entzheim ‘Terres de la Chapelle 09’	6192 ± 30	−22.2 ± 0.2	5.6 ± 0.3	3.3	Lefranc (2012)
SUERC-46454	TOTL_RL_041	Cattle, articulating 1st and 2nd phalanges, from pit 689, Entzheim ‘Terres de la Chapelle 09’	6161 ± 33	−22.0 ± 0.2	9.1 ± 0.3	3.2	Lefranc (2012)
OxA-27771	TOTL_RL_042	Cattle, articulating 1st and 2nd phalanges, from Pit 689, Entzheim ‘Terres de la Chapelle 09’	6194 ± 31	−23.81 ± 0.2	7.0 ± 0.3	3.1	Lefranc (2012)
OxA-27772	TOTL_RL_045	Pig, articulating pair of parietal and temporal unfused skull fragments, from pit 1735. Bischoffsheim ‘AFUA du Stade’	6161 ± 36	−21.01 ± 0.2	8.0 ± 0.3	3.1	Lefranc *et al*. (2004)
OxA-27773	TOTL_RL_046 (a)	Cattle, articulating left unciform and magnum from pit 1807, Bischoffsheim ‘AFUA du Stade’	6219 ± 36	−21.6 ± 0.2	9.8 ± 0.3	3.1	Lefranc *et al*. (2004)
SUERC-46499	TOTL_RL_046 (b)	Replicate of OxA-27773	6337 ± 34	−21.7 ± 0.2	9.9 ± 0.3	3.2	Lefranc *et al*. (2004)
6208 ± 25 BP, *T′* = 5.7; −21.7 ± 0.14‰, *T′* = 0.1; +9.9 ± 0.14‰, *T′* = 0.1 (*T′* (5%) = 3.8; *ν* = 1 for all)							
OxA-27774	TOTL_RL_047 (a)	*Capra hircus sp., horn* core, one of a pair both attached to articulating unfused skull, from pit 478, Entzheim ‘Terres de la Chapelle 09’	6199 ± 34	−20.8 ± 0.2	8.1 ± 0.3	3.2	Lefranc (2012)
SUERC-46500	TOTL_RL_047 (b)	Replicate of OxA-27774	6217 ± 34	−20.7 ± 0.2	8.2 ± 0.3	3.2	Lefranc (2012)
6211 ± 27 BP, *T′* = 0.1; −20.8 ± 0.14‰, *T′* = 0.1; +8.2 ± 0.21‰, *T′* = 0.1 (*T′* (5%) = 3.8, *ν* = 1 for all)							
LBK IVa2							
Ly-1568	Reich.107	Unidentified charcoal from pit 107, Reichstett	6420 ± 320				Evin *et al*. (1979)
Ly-1569	Reich.75	Unidentified charcoal from pit 75, Reichstett	6870 ± 260				Evin *et al*. (1979)
SUERC-46498	TOTL_RL_043	Cattle, refitting fragments of unfused skull from pit 1201, Bischoffsheim ‘AFUA du Stade’	6219 ± 34	−21.4 ± 0.2	9.6 ± 0.3	3.2	Lefranc *et al*. (2004)
OxA-30787	TOTL_RL_097	Pig, left radius articulating with unfused ulna (both bones missing epiphyses), from pit 33B, Rosheim ‘Lotissement Sainte Odile’	6164 ± 38	−21.0 ± 0.2	10.5 ± 0.3	3.3	Jeunesse and Lefranc (1999)
OxA-30789	TOTL_RL_098	Human, left tibia from adult male (approx.. 20–39 years old), from grave 227 at Osthouse ‘Kleinfeld’	6316 ± 38	−20.2 ± 0.2	10.5 ± 0.3	3.3	Perrin (2013)
LBK IVb							
OxA-27806	TOTL_RL_048 (a)	Pig, right proximal radius with refitting unfused epiphysis, from pit 53, Ittenheim ‘2006’	6230 ± 31	−20.6 ± 0.2	9.4 ± 0.3	3.1	Lefranc *et al*. (2010)
SUERC-46501	TOTL_RL_048 (b)	Replicate of OxA-27806	6164 ± 48	−20.8 ± 0.2	9.5 ± 0.3	3.3	Lefranc *et al*. (2010)
6282 ± 25 BP, *T′* = 1.3; −20.7 ± 0.14‰, *T′* = 0.1; +9.5 ± 0.21‰, *T′* = 0.1 (*T′* (5%) = 3.8, *ν* = 1 for all)							
SUERC-46503	TOTL_RL_050	Cattle, articulating tibia and astragalus, from pit 627, Entzheim “Terres de la Chapelle 09’	6163 ± 34	−20.8 ± 0.2	9.0 ± 0.3	3.3	Lefranc (2012)
SUERC-46507	TOTL_RL_051 (b)	Pig, articulating left radius and humerus, from pit 627, Entzheim ‘Terres de la Chapelle 09’	6196 ± 34	−20.8 ± 0.2	7.7 ± 0.3	3.3	Lefranc (2012)
SUERC-46508	TOTL_RL_052	Cattle, articulating proximal and distal sesamoids, from pit 919 Entzheim ‘Terres de la Chapelle 09’	6095 ± 34	−21.5 ± 0.2	10.0 ± 0.3	3.2	Lefranc (2012)
OxA-27775	TOTL_RL_053	Pig vertebra, articulating with neighbouring vertebra, from pit 919, Entzheim ‘Terres de la Chapelle 09’	6171 ± 36	−20.9 ± 0.2	9.6 ± 0.3	3.1	Lefranc (2012)
SUERC-46509	TOTL_RL_054	Pig vertebra found with an attached but largely unfused epiphysis, from pit 940, Bischoffseheim ‘AFUA du Stade’	6084 ± 34	−21.0 ± 0.2	6.2 ± 0.3	3.3	Lefranc *et al*. (2004)
OxA-27776	TOTL_RL_055 (a)	Sheep/goat tibia with refitting unfused epiphysis from pit 941, Bischoffsheim ‘AFUA du Stade’	6172 ± 32	−21.7 ± 0.2	8.5 ± 0.3	3.1	Lefranc *et al*. (2004)
SUERC-46510	TOTL_RL_055 (b)	Replicate of OxA-27776	6156 ± 34	−20.7 ± 0.2	9.1 ± 0.3	3.2	Lefranc *et al*. (2004)
6164 ± 24 BP, *T′* = 0.1; −21.2 ± 0.14‰, *T′* = 12.5; +8.8 ± 0.21‰, *T′* = 2.0 (*T′* (5%) = 3.8, *ν* = 1 for all)							
SUERC-46511	TOTL_RL_056	*Ovis aries* tibia, one of pair from the same animal, from Pit 3011, Rosheim ‘Rittergass’	6183 ± 34	−21.2 ± 0.2	9.2 ± 0.3	3.2	Lefranc *et al*. (2012)
OxA-27807	TOTL_RL_057	Pig, articulating mandible and maxilla, from pit 3011, Rosheim ‘Rittergass’	6202 ± 30	−20.4 ± 0.2	8.5 ± 0.3	3.1	Lefranc *et al*. (2012)
SUERC-46512	TOTL_RL_058	Pig lumbar vertebra with refitting unfused epiphysis, from pit 3039, Rosheim ‘Rittergass’	6174 ± 34	−20.5 ± 0.2	7.6 ± 0.3	3.2	Lefranc *et al*. (2012)
OxA-27808	TOTL_RL_059	*Capra hircus*, articulated radius and ulna, from pit 3039, Rosheim ‘Rittergass’	6222 ± 31	−20.2 ± 0.2	8.8 ± 0.3	3.1	Lefranc *et al*. (2012)
SUERC-46513	TOTL_RL_060	Pig, articulating mandible and maxilla, from pit 3034, Rosheim ‘Rittergass’	6185 ± 34	−20.5 ± 0.2	9.1 ± 0.3	3.2	Lefranc *et al*. (2012)
OxA-27809	TOTL_RL_061	Sheep/goat mandible, 1 of a pair from the same animal, from pit 3034, Rosheim ‘Rittergass’	6274 ± 31	−19.7 ± 0.2	10.5 ± 0.3	3.1	Lefranc *et al*. (2012)
SUERC-55330	TOTL_RL_099	Cattle navicular cuboid articulating with other foot bones, from pit 627, spit E11 and E12, Entzheim ‘Terres de la Chapelle 09’	6231 ± 29	−21.2 ± 0.2	8.9 ± 0.3	3.3	Lefranc (2012)
OxA-30788	TOTL_RL_100A	Pig unfused lateral metapodial, epiphysis missing but judged to have been present when the bone entered the ground, from pit 919, square C2, Entzheim ‘Terres de la Chapelle 09’	6202 ± 38	−21.1 ± 0.2	6.4 ± 0.3	3.2	Lefranc (2012)
SUERC-55331	TOTL_RL_100B	Replicate of OxA-30788	6226 ± 34	−20.8 ± 0.2	7.0 ± 0.3	3.2	Lefranc (2012)
6215 ± 26 BP, *T′* = 0.2; −20.1 ± 0.14‰, *T′* = 1.1; +6.7 ± 0.21‰, *T′* = 2.0 (*T′* (5%) = 3.8, *ν* = 1 for all)

**Table 2:** Radiocarbon and stable isotopic measurements for the Middle Neolithic ceramic sequence in Lower Alsace (highest posterior density intervals are provided for samples of intrinsic interest and are derived from the model defined in Figs. 15 and 16; reported stable isotope values were measured by isotopic ratio mass spectrometry)

Laboratory number	Sample/find number	Material, stratigraphic information and site	Radiocarbon age (BP)	δ^13^C (‰)	δ^15^N (‰)	C/N	Highest posterior density interval (95% probability)	References
Hinkelstein								
Poz-34074	5046-OHU-7056	Human bone from articulated adult? female skeleton in grave 7056 at Entzheim ‘Lotissement d′activités Entzheim 4’	5940 ± 40				*4830–4720 cal BC*	Leprovost and Queyras (2011)
SUERC-60027	Grave 2	Femur from articulated child skeleton (10–11 years), grave 2, Remseck-Aldingen ‘Halden’	5827 ± 29	−20.1 ± 0.3	9.2 ± 0.3	3.3	*4790–4715 cal BC*	Joachim (1993)
SUERC-60026	Grave 7	Right tibia from articulated adult skeleton, grave 7, Remseck-Aldingen ‘Halden’	5904 ± 29	−20.4 ± 0.3	9.2 ± 0.4	3.2	*4810–4720 cal BC*	Joachim (1993)
Poz-33546		Human bone from articulated adult skeleton in grave 2 at Erstein ‘Krebsrott’	5880 ± 40				*4805–4715 cal BC*	Denaire (2011), p. 26; Jeunesse and Arbogast (1997), p. 72; Spatz (1996), p. 427
Grossgartach								
Poz-34073	5046-OHU-7043	Human bone from articulated sub-adult skeleton (15–20 years) in grave 7043 at Entzheim ‘Lotissement d′activités Entzheim 4’	5870 ± 40				*4750–4665 cal BC*	Denaire (2011, p. 26) and Leprovost and Queyras (2011, p. 123)
Poz-34077	5046-OHU-7105	Human bone from articulated child skeleton (2–4 years) in grave 7105 at Entzheim ‘Lotissement d′activités Entzheim 4’	5870 ± 40				*4750–4665 cal BC*	Denaire (2011, p. 26) and Leprovost and Queyras (2011, p. 123)
Poz-34091	5046-OHU-7112	Human bone from articulated adult skeleton in grave 7112 at Entzheim ‘Lotissement d′activités Entzheim 4’	5770 ± 40				*4725–4635 cal BC*	Denaire (2011, p. 26); Leprovost and Queyras (2011, p. 123)
Poz-34081	5046-OHU-7121	Human bone from articulated sub-adult skeleton (12–18 years) in grave 7121 at Entzheim ‘Lotissement d′activités Entzheim 4’	5790 ± 50				*4730–4635 cal BC*	Denaire (2011, p. 26) and Leprovost and Queyras (2011, p.123)
Poz-34083	5046-OHU-7126	Human bone articulated skeleton in grave 7126 at Entzheim ‘Lotissement d′activités Entzheim 4’	5750 ± 40				*4720–4635 cal BC*	Denaire (2011, p. 26) and Leprovost and Queyras (2011, p. 123)
OxA-27810	TOTL_RL_001	Cranium from articulated female adult skeleton 40770, from grave 42 at Lingolsheim ‘Sablières Fischer et Schott’	5870 ± 32	−20.1 ± 0.2	9.8 ± 0.3	3.3	*4750–4680 cal BC*	Denaire (2009, 2011) and Forrer (1911a, b, 1912, 1922, 1938)
SUERC-46273	TOTL_RL_002	Right femur from articulated adult skeleton 5539 in grave 7 at Erstein ‘Krebsrott’	5812 ± 33	−20.1 ± 0.2	10.0 ± 0.3	3.3	*4725–4640 cal BC*	Denaire (2011, p. 26), Jeunesse and Arbogast (1997, p. 72) and Spatz (1996, p. 427)
SUERC-46274	TOTL_RL_003 (b)	Right (?) femur from articulated child skeleton (1–4 years) in grave 85 at Rosheim ‘Rosenmeer’	5839 ± 33	−19.7 ± 0.2	12.7 ± 0.3	3.2	*4735–4645 cal BC*	Denaire (2009, 2011)
Poz-34072	5046-OHU-7007	Human bone from articulated child skeleton (2–3 years) in grave 7007 at Entzheim ‘Lotissement d′activités Entzheim 4’	5840 ± 40				*4745–4650 cal BC*	Denaire (2011, p. 26) and Leprovost and Queyras (2011, p. 123)
Poz-34076	5046-OHU-7103	Human bone from articulated adult? male skeleton in grave 7103 at Entzheim ‘Lotissement d′activités Entzheim 4’	5810 ± 40				*4735–4635 cal BC*	Denaire (2011, p. 26) and Leprovost and Queyras (2011, p. 123)
Poz-34079	5046-OHU-7114	Human bone from articulated adult female skeleton in grave 7114 at Entzheim ‘Lotissement d′activités Entzheim 4’	5920 ± 40				*4750–4685 cal BC*	Denaire (2011, p. 26) and Leprovost and Queyras (2011, p. 123)
Poz-34092	5046-OHU-7117	Human bone from articulated child skeleton (2–4 years) in grave 7117 at Entzheim ‘Lotissement d′activités Entzheim 4’	5890 ± 40				*4750–4675 cal BC*	Denaire (2011, p. 26) and Leprovost and Queyras (2011, p. 123)
Poz-34080	5046-OHU-7119	Human bone from articulated adult? female skeleton in grave 7119 at Entzheim ‘Lotissement d′activités Entzheim 4’	5790 ± 50				*4730–4635 cal BC*	Denaire (2011, p. 26) and Leprovost and Queyras (2011, p. 123)
Poz-34093	5046-OHU-7125	Human bone from articulated child skeleton (7–11 years) in grave 7125 at Entzheim ‘Lotissement d′activités Entzheim 4’	5760 ± 50				*4725–4635 cal BC*	Denaire (2011, p. 26) and Leprovost and Queyras (2011, p. 123)
OxA-27811	TOTL_RL_005	Sheep/goat radius with refitting unfused epiphysis from pit 656, Kolbsheim ‘Vogeseblick’	5848 ± 31	−18.9 ± 0.2	9.1 ± 0.3	3.3	*4745–4660 cal BC*	Denaire (2013)
SUERC-46517	TOTL_RL_006	Sheep/goat paired phalanges with refitting proximal unfused epiphyses from pit 656, Kolbsheim ‘Vogeseblick’	5804 ± 34	−19.8 ± 0.2	9.6 ± 0.3	3.2	*4725–4640 cal BC*	Denaire (2013)
SUERC-46275	TOTL_RL_007 (a)	Right femur from articulated adult male skeleton in grave 76 at Rosheim ‘Rosenmeer’	5767 ± 31	−20.3 ± 0.2	10.2 ± 0.3	3.2	*4720–4640 cal BC*	Denaire (2009, 2011)
OxA-27812	TOTL_RL_007 (b)	Replicate of SUERC-46275	5865 ± 31	−20.2 ± 0.2	9.8 ± 0.3	3.3		
5816 ± 22 BP, *T′* = 5.0; −20.3 ± 0.14‰, *T′* = 0.1, +10.0 ± 0.21‰, *T′* = 0.9 (*T′* (5%) = 3.8, *ν* = 1 for all)								
SUERC-46276	TOTL_RL_008	Right(?) humerus(?) from articulated baby skeleton (10–12 months) from grave 89 at Rosheim ‘Rosenmeer’	5732 ± 33	−20.1 ± 0.2	9.8 ± 0.3	3.2	*4705–4635 cal BC*	Denaire (2009, 2011)
OxA-27813	TOTL_RL_009	Right femur from articulated adult female skeleton in grave 112 at Rosheim ‘Rosenmeer’	5845 ± 33	−20.0 ± 0.2	9.7 ± 0.3	3.3	*4745–4655 cal BC*	Denaire (2009, 2011)
OxA-27814	TOTL_RL_010 (a)	Left femur from articulated sub-adult skeleton (14–19 years) in grave 45 at Rosheim ‘Rosenmeer’	5833 ± 29	−20.2 ± 0.2	9.7 ± 0.3	3.3	*4730–4655 cal BC*	Denaire (2009, 2011)
SUERC-46277	TOTL_RL_010 (b)	Replicate of OxA-27814	5830 ± 33	−20.5 ± 0.2	10.0 ± 0.3	3.2		
5832 ± 22 BP, *T′* = 0.0; −20.4 ± 0.14‰; *T′* = 1.1; +9.9 ± 0.21‰, *T′* = 0.5 (*T′* (5%) = 3.8, *ν* = 1 for all)								
Poz-34094	5046-OHU-7157	Human bone from articulated child skeleton (6–7 years) in grave 7157 at Entzheim ‘Lotissement d′activités Entzheim 4’	5830 ± 40				*4740–4645 cal BC*	Denaire (2011, p. 26) and Leprovost and Queyras (2011, p. 123)
Poz-34086	5046-OHU-7162	Human bone from articulated child skeleton (4–8 years) in grave 7162 at Entzheim ‘Lotissement d′activités Entzheim 4’	5800 ± 40				*4730–4640 cal BC*	Denaire (2011, p. 26) and Leprovost and Queyras (2011, p. 123)
SUERC-46278	TOTL_RL_011	Left femur from articulated adult female skeleton in grave 108 at Rosheim ‘Rosenmeer’	5787 ± 33	−20.3 ± 0.2	8.8 ± 0.3	3.2	*4720–4635 cal BC*	Denaire (2009, 2011)
OxA-27815	TOTL_RL_012	Right femur from articulated adult male skeleton in grave 91 at Rosheim ‘Rosenmeer’	5812 ± 30	−20.2 ± 0.2	9.0 ± 0.3	3.3	*4730–4645 cal BC*	Denaire (2009, 2011)
SUERC-46279	TOTL_RL_013	Right femur from articulated sub-adult skeleton (15–19 years) in grave 88 at Rosheim ‘Rosenmeer’	5792 ± 33	−20.3 ± 0.2	9.4 ± 0.3	3.2	*4720–4635 cal BC*	Denaire (2009, 2011)
OxA-27816	TOTL_RL_014	Right femur from articulated sub-adult skeleton (15–19 years) in grave 83 at Rosheim ‘Rosenmeer’	5898 ± 29	−20.3 ± 0.2	8.4 ± 0.3	3.3	*4750–4690 cal BC*	Denaire (2009, 2011)
SUERC-46283	TOTL_RL_015	Right femur from articulated adult male skeleton in grave 100 at Rosheim ‘Rosenmeer’	5789 ± 33	−20.2 ± 0.2	10.0 ± 0.3	3.2	*4720–4635 cal BC*	Denaire (2009, 2011)
OxA-27817	TOTL_RL_016	Right femur from articulated adult female skeleton in grave 95 at Rosheim ‘Rosenmeer’	5847 ± 30	−20.4 ± 0.2	8.7 ± 0.3	3.3	*4745–4660 cal BC*	Denaire (2009, 2011)
SUERC-46284	TOTL_RL_017	Right femur from articulated adult female skeleton in grave 111 at Rosheim ‘Rosenmeer’	5791 ± 33	−20.7 ± 0.2	9.3 ± 0.3	3.2	*4720–4635 cal BC*	Denaire (2009, 2011)
OxA-27818	TOTL_RL_018	Right femur from articulated baby skeleton (3–5 months) in grave 87 at Rosheim ‘Rosenmeer’	5885 ± 31	−19.4 ± 0.2	10.6 ± 0.3	3.3	*4750–4685 cal BC*	Denaire (2009, 2011)
SUERC-46435	TOTL_RL_019	Right femur from articulated adult female skeleton in grave 82 at Rosheim ‘Rosenmeer’	5816 ± 28	−20.5 ± 0.2	9.5 ± 0.3	3.3	*4725–4640 cal BC*	Denaire (2009, 2011)
OxA-27819	TOTL_RL_020 (a)	Right femur from articulated adult skeleton 5554-A in grave 19 at Erstein ‘Krebsrott’	5823 ± 30	−20.4 ± 0.2	8.0 ± 0.3	3.3	*4730–4650 cal BC*	Denaire (2011, p. 26), Jeunesse and Arbogast (1997, p. 72) and Spatz (1996, p. 427)
SUERC-46436	TOTL_RL_020 (b)	Replicate of OxA-27819	5834 ± 33	−20.4 ± 0.2	8.6 ± 0.3	3.2		
5828 ± 23 BP, *T′* = 0.1; −20.4 ± 0.14‰, *T′* = 0.0; +8.3 ± 0.21‰, *T′* = 2.0 (*T′* (5%) = 3.8, *ν* = 1 for all)								
OxA-27820	TOTL_RL_021	Right femur from articulated adult skeleton 5562 in grave 28 at Erstein ‘Krebsrott’	5888 ± 31	−20.4 ± 0.2	9.7 ± 0.3	3.4	*4750–4685 cal BC*	Denaire (2011, p. 26), Jeunesse and Arbogast (1997, p. 72) and Spatz (1996, p. 427)
SUERC-46440	TOTL_RL_022	Right femur from articulated adult female skeleton 36819–823 in grave 37 at Lingolsheim ‘Sablières Fischer et Schott’	5768 ± 30	−20.7 ± 0.2	9.4 ± 0.3	3.3	*4715–4635 cal BC*	Lichardus-Itten (1980)
Planig-Friedberg								
OxA-27821	TOTL_RL_023 (a)	Right femur from articulated adult female skeleton in grave 5 at Rosheim ‘Rosenmeer’	5800 ± 31	−20.6 ± 0.2	10.1 ± 0.3	3.2	*4680–4595 cal BC*	Denaire (2009, 2011)
SUERC-46441	TOTL_RL_023 (b)	Replicate of OxA-27821	5800 ± 30	−20.7 ± 0.2	10.5 ± 0.3	3.2		Denaire (2009, 2011)
5800 ± 22 BP, *T′* = 0.0; −20.7 ± 0.14‰, *T′* = 0.1; +10.3 ± 0.21‰,*T′* = 0.9 (*T′* (5%) = 3.8, *ν* = 1 for all)								
SUERC-46442	TOTL_RL_024	Right femur from articulated adult skeleton in grave 42 at Rosheim ‘Rosenmeer’	5762 ± 33	−20.5 ± 0.3	10.0 ± 0.3	3.2	*4680–4590 cal BC*	Denaire (2009, 2011)
OxA-30277	TOTL_RL_072	Right femur from articulated adult female skeleton in grave 4008 at Obernai ‘PAEI’	5809 ± 33	−20.2 ± 0.2	9.3 ± 0.3	3.2	*4680–4595 cal BC*	Lefranc *et al*. (in prep)
SUERC-52378	TOTL_RL_073	Right femur from articulated adult skeleton from grave 4015, Obernai	5743 ± 30	−20.8 ± 0.2	9.7 ± 0.3	3.2	*4680–4585 cal BC*	Lefranc *et al*. (in prep)
OxA-30278	TOTL_RL_074	Left femur from articulated adult female skeleton in grave 4022 at Obernai ‘PAEI’	5744 ± 33	−20.2 ± 0.2	9.2 ± 0.3	3.3	*4680–4585 cal BC*	Lefranc *et al*. (in prep)
Poz-50940		Human bone from articulated adult female skeleton in grave 39 at Guémar ‘Rotenberger Weg’	5730 ± 50				*4680–4585 cal BC*	Denaire and Mauvilly (2012)
Rössen								
SUERC-46443	TOTL_RL_025	Left femur from articulated adult female skeleton in grave 36 at Rosheim ‘Rosenmeer’	5753 ± 33	−20.6 ± 0.2	9.6 ± 0.3	3.2	*4635–4500 cal BC*	Denaire (2009, 2011)
OxA-27972	TOTL_RL_026 (a)	Right femur from articulated adult skeleton in grave 50 at Rosheim ‘Rosenmeer’	5772 ± 35	−20.4 ± 0.2	10.2 ± 0.3	3.3	*4640–4540 cal BC*	Denaire (2009, 2011)
OxA-27973	TOTL_RL_026 (a)	Replicate of OxA-27972	5803 ± 32	−20.1 ± 0.2	10.3 ± 0.3	3.3		
SUERC-46444	TOTL_RL_026 (b)	Replicate of OxA-27972	5735 ± 32	−20.5 ± 0.2	10.1 ± 0.3	3.3		
5770 ± 20 BP, *T′* = 2.3; −20.3 ± 0.12‰, *T′* = 2.2; +10.2 ± 0.17‰, *T′* = 0.2 (*T′* (5%) = 6.0, *ν* = 2 for all)								
OxA-27822	TOTL_RL_027 (a)	Right femur from articulated adult male skeleton in grave 55 at Rosheim ‘Rosenmeer’	5804 ± 30	−20.1 ± 0.2	10.0 ± 0.3	3.2	*4645–4540 cal BC*	Denaire (2009, 2011)
SUERC-46445	TOTL_RL_027 (b)	Replicate of OxA-27822	5731 ± 30	−20.3 ± 0.2	10.3 ± 0.3	3.2		
5768 ± 22 BP, *T′* = 3.0; −20.2 ± 0.14‰, *T′* = 0.5; +10.3 ± 0.21‰, *T′* = 0.0 (T′(5%) = 3.8, *ν* = 1 for all)								
OxA-27823	TOTL_RL_028 (a)	Cattle, articulating metacarpal and phalanx from pit 116, Meistratzheim ‘Station Intercommunale d′épuration’	5749 ± 29	−21.9 ± 0.2	6.0 ± 0.3	3.4	–	Denaire (2011) and Perrin (2011)
SUERC-46446	TOTL_RL_028 (b)	Replicate of OxA-27823	5686 ± 30	−22.1 ± 0.2	6.3 ± 0.3	3.2		
5719 ± 21 BP, *T′* = 2.3; −22.0 ± 0.14‰, *T′* = 0.5; +6.2 ± 0.21‰, *T′* = 0.5 (T′(5%) = 3.8, *ν* = 1 for all)								
SUERC-46450	TOTL_RL_029	Cattle, articulating metacarpal and phalanx from pit 116, Meistratzheim ‘Station Intercommunale d′épuration’	5649 ± 32	−21.4 ± 0.2	7.2 ± 0.3	3.3	–	Denaire (2011) and Perrin (2011)
SUERC-52377	TOTL_RL_075	Mandible from an articulated child skeleton (0–4 years) in grave 4106 at Obernai ‘PAIE’	5697 ± 32	−20.1 ± 0.2	13.4 ± 0.3	3.3	*4600–4460 cal BC*	Lefranc *et al*. (in prep)
GrA-45797		Human bone from articulated child (6–8 years) skeleton (individual 3) in grave 17 at Entzheim ‘Les Terres de la Chapelle 1–Lotissement’	5660 ± 40				*4550–4455 cal BC*	Lefranc (2012)
GrA-45953		Human bone from articulated adult skeleton (individual 1) in grave 17 at Entzheim ‘Les Terres de la Chapelle 1–Lotissement’	5665 ± 40					
Poz-32444		Animal bone from pit 116, Meistratzheim ‘Station Intercommunale d′épuration’	5750 ± 70				–	Denaire (2011) and Perrin (2011)
Poz-32445		Animal bone from pit 116, Meistratzheim ‘Station Intercommunale d′épuration’	5690 ± 40				–	Denaire (2011) and Perrin (2011)
Poz-32446		Animal bone from pit 116, Meistratzheim ‘Station Intercommunale d′épuration’	5780 ± 50				–	Denaire (2011) and Perrin (2011)
Poz-33544		Animal bone from pit 113, Meistratzheim ‘Station Intercommunale d′épuration’	5680 ± 50				–	Denaire (2011) and Perrin (2011)
Bischheim								
OxA-30279	TOTL_RL_077 (a)	Human burial, right femur from a mature adult female, grave 0010 (square 17), Schwindratzheim ‘Les Terrasses de la Zorn’	5530 ± 32	−20.2 ± 0.2	11.0 ± 0.3	3.3	*4445–4415 cal BC (7%)* or *4400–4380 cal BC (2%)* or *4375–4325 cal BC (86%)*	Denaire *et al*. (2014)
SUERC-52370	TOTL_RL_077 (b)	Replicate of OxA-30279	5487 ± 32	−20.7 ± 0.2	11.8 ± 0.3	3.2		
5509 ± 23 BP, *T′* = 0.9; −20.5 ± 0.14‰, *T′* = 3.1; +11.4 ± 0.21‰, *T′* = 3.6 (*T′* (5%) = 3.8, *ν* = 1 for all)								
SUERC-52371	TOTL_RL_078	Cattle right pre-maxilla, one of a pair (so probably from a fleshed animal or part thereof), from pit 552, Schwindratzheim ‘Les Terrasses de la Zorn’	5395 ± 29	−22.0 ± 0.2	7.4 ± 0.3	3.2	–	Denaire *et al*. (2014)
SUERC-52375	TOTL_RL_079	Pig left mandible; 1 of a pair (so probably from a fleshed animal or part thereof), from pit 875, Schwindratzheim ‘Les Terrasses de la Zorn’	5392 ± 32	−20.4 ± 0.2	7.7 ± 0.3	3.2	–	Denaire *et al*. (2014)
OxA-30280	TOTL_RL_080 (a)	Pig unfused right metapodial with refitting epiphysis, from pit 875, Schwindratzheim ‘Les Terrasses de la Zorn’	5350 ± 32	−22.0 ± 0.2	8.5 ± 0.3	3.3	–	Denaire *et al*. (2014)
SUERC-52376	TOTL_RL_080 (b)	Replicate of OxA-30280	5489 ± 31	−22.3 ± 0.2	9.6 ± 0.3	3.2		
UBA-27378	TOTL_RL_080 (c)	Replicate of OxA-30280	5307 ± 53					
5406 ± 21 BP, *T′* = 13.7, *T′* (5%) = 6.0, *ν* = 2; −22.2 ± 0.14‰, *T′* = 1.1, *T′* (5%) = 3.8, *ν* = 1; +9.1 ± 0.21‰, *T′* = 6.7, *T′* (5%) = 3.8, *ν* = 1								
SUERC-52397	TOTL_RL_081	Human burial, left femur from articulated child skeleton (3–6 years), grave 722, Entzheim ‘Les Terres de la Chapelle 2’	5649 ± 30	−20.6 ± 0.2	10.3 ± 0.3	3.2	*4490–4370 cal BC*	Lefranc (2012)
Poz-45609		Articulated child skeleton (1–4 years) from well 142 at Obernai ‘Schulbach’	5610 ± 40				*4455–4365 cal BC*	Croutsch *et al*. (2014)
Poz-45612		Articulated dog burial from well 142 at Obernai ‘Schulbach’	5600 ± 40				*4475–4375 cal BC*	Croutsch *et al*. (2014)
Poz-45620		Unidentified charcoal from well 142 at Obernai ‘Schulbach’	5610 ± 40				–	Croutsch *et al*. (2014)
Brubach-Oberbergen								
OxA-30019	TOTL_RL_082	Carbonised residue on Bruebach-Oberbergen sherd from pit 3175, Duntzenheim ‘Frauabwand’	5430 ± 31	−26.3 ± 0.2			*4345–4250 cal BC*	Lefranc (2011)
OxA-30053	TOTL_RL_083	Carbonised residue (pitch?) on Bruebach-Oberbergen sherd from pit 3176, Duntzenheim ‘Frauabwand’	5536 ± 31	−27.0 ± 0.2			*4405–4330 cal BC*	Lefranc (2011)
OxA-30020	TOTL_RL_084	Carbonised residue on Bruebach-Oberbergen sherd from pit 3176, Duntzenheim ‘Frauabwand’	5426 ± 33	−24.9 ± 0.2			*4345–4245 cal BC*	Lefranc (2011)
SUERC-52398	TOTL_RL_085	Right femur from articulated adult skeleton, from pit 107 at Vendenheim ‘Lotissment des Portes du Kochersberg’	5524 ± 32	−19.9 ± 0.2	10.0 ± 0.3	3.2	*4405–4325 cal BC*	Lefranc *et al*. (2015)
SUERC-55323	TOTL_RL_104	Left femur from articulated adult skeleton from grave 509 at Kolbsheim ‘Vogeseblick’	5415 ± 31	−20.3 ± 0.2	9.6 ± 0.3	3.2	*4340–4245 cal BC*	Denaire (2013)
Poz-45610		Animal bone from pit 27 at Obernai ‘Schulbach’	5550 ± 40				–	Croutsch *et al*. (2014)
Poz-45611		Animal bone from pit 121 at Obernai ‘Schulbach’	5640 ± 40				–	Croutsch *et al*. (2014)
Poz-45614		Cereal grain from pit 27, Obernai ‘Schulbach’	5630 ± 35				–	Croutsch *et al*. (2014)
Poz-45615		Cereal grain from pit 121, Obernai ‘Schulbach’	5660 ± 40				–	Croutsch *et al*. (2014)
Poz-45616		Cereal grain from well 167, Obernai ‘Schulbach’	5590 ± 40				–	Croutsch *et al*. (2014)
Poz-45618		Unidentified charcoal from pit 27, Obernai ‘Schulbach’	5630 ± 40				–	Croutsch *et al*. (2014)
Poz-45621		Unidentified charcoal from well 167, Obernai ‘Schulbach’	5610 ± 50				–	Croutsch *et al*. (2014)
Poz-45762		Unidentified charcoal from pit 121, Obernai ‘Schulbach’	5640 ± 40				–	Croutsch *et al*. (2014)
BORS I								
OxA-30281	TOTL_RL_087	Right humerus from almost complete hare skeleton from pit 10 at Bischoffsheim ‘Rue du Stade’	5338 ± 32	−22.1 ± 0.2	5.5 ± 0.3	3.3	–	Jeunesse *et al*. (2003)
SUERC-52399	TOTL_RL_088	Cattle tibia with refitting unfused epiphysis from pit 12 at Bischoffsheim ‘Rue du Stade’	5277 ± 28	−22.0 ± 0.2	6.1 ± 0.3	3.2	–	Jeunesse *et al*. (2003)
OxA-30282	TOTL_RL_089 (a)	Pig, articulating 3rd and 4th metatarsals from pit 12 at Bischoffsheim ‘Rue du Stade’	5273 ± 34	−20.8 ± 0.2	7.7 ± 0.3	3.3	–	Jeunesse *et al*. (2003)
SUERC-52400	TOTL_RL_089 (b)	Replicate of OxA-30282	5391 ± 32	−21.4 ± 0.2	8.5 ± 0.3	3.2		
5336 ± 24 BP, *T′* = 6.4; −21.1 ± 0.14‰, *T′* = 4.5; +8.1 ± 0.21‰, *T′* = 3.6 (*T′* (5%) = 3.8, *ν* = 1 for all)								
SUERC-55321	TOTL_RL_090 (b)	Pig, articulating? left radius and ulna from pit 31, Bischoffsheim ‘Rue du Stade’	5274 ± 33	−20.4 ± 0.2	9.2 ± 0.3	3.2	–	Jeunesse *et al*. (2003)
SUERC-55322	TOTL_RL_102	Sheep/goat 1st phalanx with refitting unfused epiphysis from pit 38 at Bischoffsheim ‘Rue du Stade’	5361 ± 31	−20.3 ± 0.2	6.6 ± 0.3	3.2	–	Jeunesse *et al*. (2003)
BORS II								
UBA-27377	TOTL_RL_091 (c)	Medium-sized mammal lumbar vertebra with refitting unfused epiphysis from the base of pit 14 at Geispolsheim ‘Schlossgarten’	5208 ± 52				–	Denaire (2013)
Poz-46043		Pig bone from pit 14 at Geispolsheim ‘Schlossgarten’	5260 ± 40				–	Denaire (2013)

As part of this project, replicate measurements were undertaken on 17 samples (three of which have three radiocarbon measurements). Thirteen of these samples produced radiocarbon ages that are statistically consistent at 95% confidence, two more have measurements that are statistically inconsistent at 95% confidence, but statistically consistent at 99% confidence, and two have results that are divergent at more than 99% confidence (Ward and Wilson 1978; Tables 1 and 2). This variance is slightly more than would be expected on purely statistical grounds although, when weighted means are taken of these measurements, all have good individual agreement in the models presented (Bronk Ramsey 1995, p. 429). This suggests that our modelled chronologies are robust in the face of the observed inter-laboratory reproducibility.

Of the 17 replicate groups of δ^13^C measurements, 15 are statistically consistent at 95% confidence, one is inconsistent at 95% confidence but consistent at 99% confidence, and one is inconsistent at more than 99% confidence. For δ^15^N all but one of the replicate groups are statistically consistent at 95% confidence, the remaining one being divergent at slightly more than 99% confidence. This variation is within statistical expectation.

### Radiocarbon Calibration and Bayesian Chronological Modelling

The Bayesian chronological modelling has been undertaken using the programme OxCal v4.2 (Bronk Ramsey 2009a, b; Bronk Ramsey and Lee 2013) and the atmospheric calibration curve for the northern hemisphere published by Reimer et al. (2013). Mixed-source calibration has been used in the sensitivity analysis and is fully described below. The algorithms used are defined exactly by the brackets and OxCal keywords on the left-hand side of Figs. 8, 15, 16 and 23. The OxCal CQL2 code for all models is provided as Electronic Supplementary Material (http://c14.arch.ox.ac.uk/). The posterior density estimates output by the model are shown in the figures in black, with the unconstrained calibrated radiocarbon dates shown in outline. The other distributions correspond to aspects of the model. For example, the distribution ‘*LBK IIb/LBK IIc*’ (Fig. 8) is the posterior density estimate for the time when pottery characteristic of phase LBK IIb was replaced by that characteristic of phase LBK IIc in Lower Alsace. In the text and tables, the highest posterior density intervals of the posterior density estimates are given *in italics*.

The chronological models described below have been constructed using abutting uniform phases, with flexible trapezium phases for the start of the LBK sequence and for the start and end of the Middle Neolithic sequence. Archaeologically this allows for a gradual introduction and demise of the different traditions and for the continuous frequency of changes within each seriation. The statistical methods are fully described by Buck et al. (1992), Karlsberg (2006) and Lee and Bronk Ramsey (2012). Their implementation in OxCal is described by Bronk Ramsey (1995) and Bronk Ramsey and Lee (2013).

## LBK Pottery from Lower Alsace: Types, Motifs and Sequence

The correspondence analysis of LBK ceramics in Lower Alsace used 112 assemblages from 20 settlements (Fig. 2; Electronic Supplementary Material Matrix 1) and was based on 87 typological criteria (Lefranc 2007). Those include decorative motifs on rims, the types of bands which formed the principal decorative structure, and the secondary motifs which occur in the spaces in between. In order to obtain a parabola with clear differentiation between successive phases, we have chosen chronologically sensitive types and assemblages in order to maximise the temporal trends in the material.

**Fig. 5:**
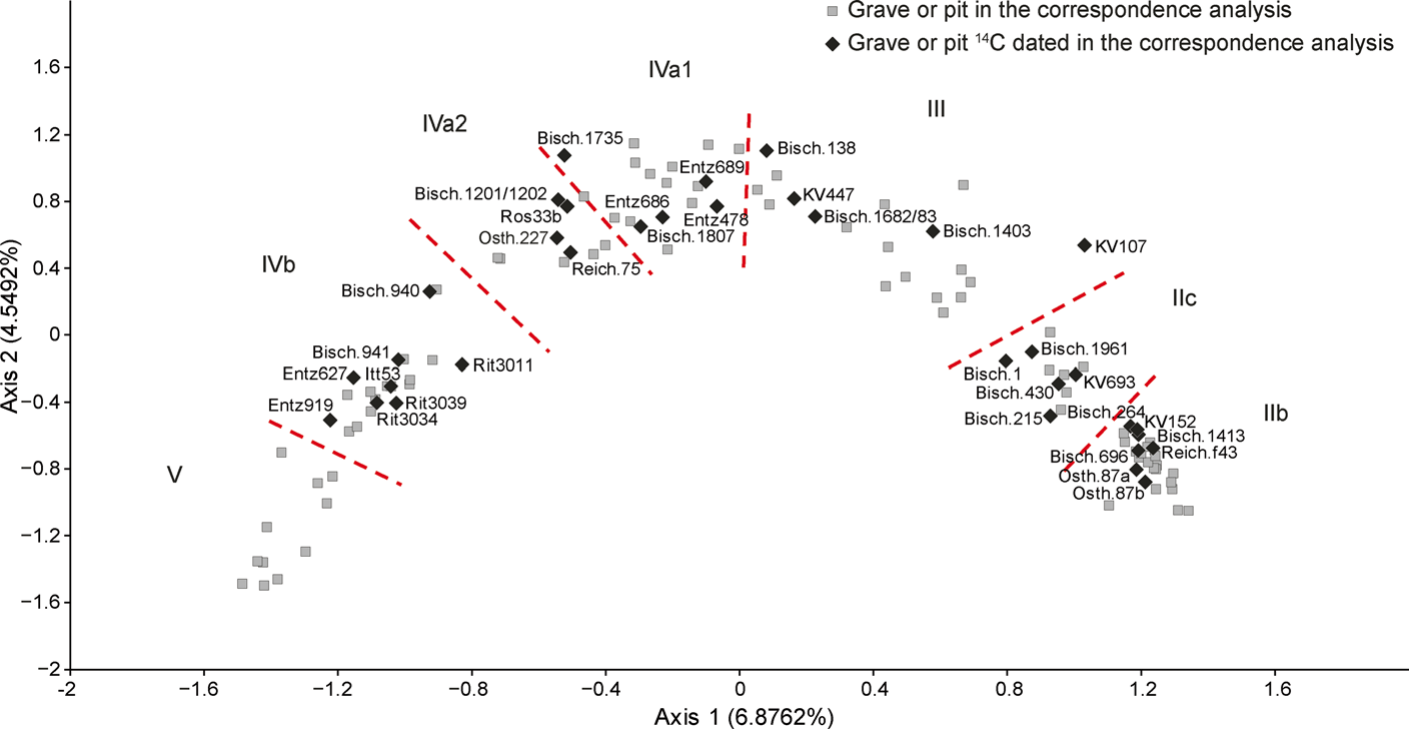
Correspondence analysis of LBK ceramics in Lower Alsace

The correspondence analysis was partitioned into seven phases, seen from right to left across Fig. 5. We have interpreted these as showing temporal sequence. The parabola is easy to put into context. The first phase includes assemblages equivalent to the Flomborn style of the earlier LBK in central Europe, and the last, that of the Dachstein style, traditionally identified as the final LBK style in Lower Alsace. The succession of chronological phases defined by the seriation is confirmed by numerous stratigraphic relationships in the settlements of the region.

The structure of the chronological scheme of Meier-Arendt (1966), used since the first systematic periodisations of the LBK in Alsace, has been retained, for purposes of comparability. In that scheme, the LBK was divided into five main stages: the earliest LBK I (*älteste* LBK), an early LBK II, a middle LBK III, a late LBK IV and a final LBK V.

All but the first of these typological stages can be seen in the correspondence analysis for Lower Alsace, which enables the subdivision of LBK II into two, phases LBK IIb and IIc, and LBK IV into three phases, LBK IVa1, IVa2 and IVb. It has not proved possible to subdivide the middle (LBK III) and final stages (LBK V).

**Fig. 6:**
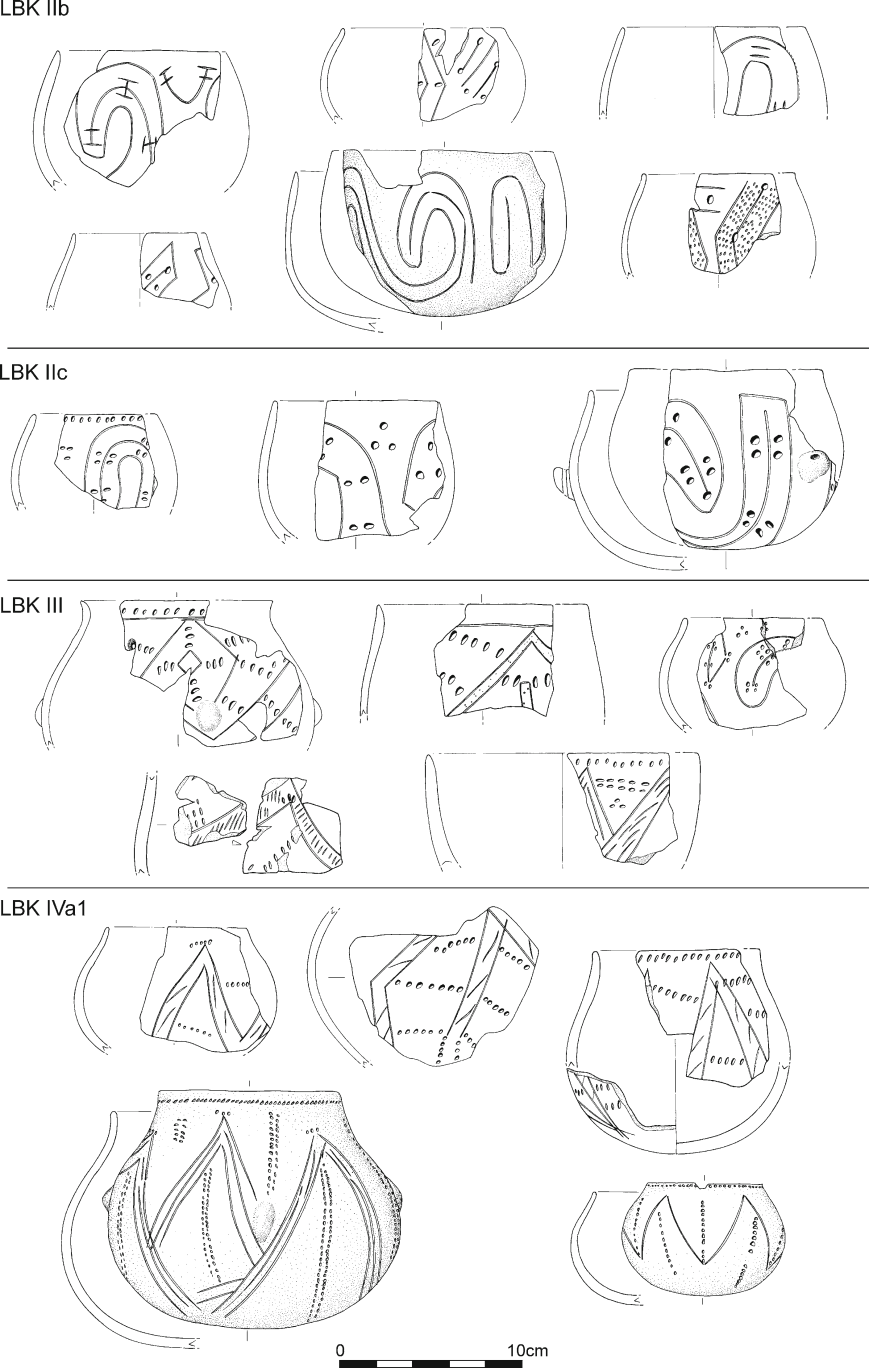
Typical decorative motifs for LBK pottery belonging to phases IIb, IIc, III and IVa1 of the LBK seriation for Lower Alsace (Fig. 5; Electronic Supplementary Material Matrix 1)

The 26 assemblages which constitute the first phase of the seriation have vessels decorated in Flomborn style (Fig. 6). Rims were plain, and the principal decorative elements covering vessel surfaces are big geometric patterns, such as wavy lines and spirals, formed by wide bands often interrupted at intervals by big circular impressions, in pairs or double pairs. Bands filled by scattered impressions are equally frequent. Secondary motifs are extremely varied. The most frequent are incisions, stabbed and not stabbed, and then circular impressions, cruciform motifs, spirals and isolated or paired impressions. Phase IIb of this Lower Alsace seriation can be synchronised with phase 2 from the seriation of the south-western LBK (Strien 2000). The early Flomborn style, characterised by unfilled bands, is well represented in the Neckar valley but so far absent from Alsace (where it would correspond to LBK IIa).

The second phase, constituted by 11 assemblages, shares many motifs with LBK IIb but new decorative motifs presage the middle LBK or LBK III. For the first time rim decoration appears, in single rows of impressions, sometimes underlined by a groove (Fig. 6). These contexts are assigned to LBK IIc, corresponding to late Flomborn. This is characterised by transitional assemblages with vessels combining decoration typical of LBK IIb and new motifs which become more common in the next phase.

The third phase, which is formed by material from 19 pits, defines the middle LBK or LBK III. This has the majority of motifs previously seen in IIb and also numerous new decorative motifs of which the most characteristic are the alternating incisions and impressions on rims and the bands interrupted by pairs or sets of impressions. Traditional Flomborn decoration such as the spiral gives way to angular or wavy-line elements (Fig. 6). The secondary motifs of the early LBK disappear, to be replaced by short impressed lines.

The fourth phase, based on material from 18 pits, is defined as LBK IVa1, the start of the late LBK. Up to 85% of decoration is angular, though there are still some spirals. These are made by ribbons or bands formed by triple narrow incised lines. There are fewer interruptions to bands, and spaced oblique hatching within bands develops. Rims are most often decorated by single rows of impressions, though sometimes by two such rows. Secondary motifs now include new impressed segments, though mostly rather similar to those seen in LBK III. Small ‘comma’ impressions are very characteristic.

**Fig. 7:**
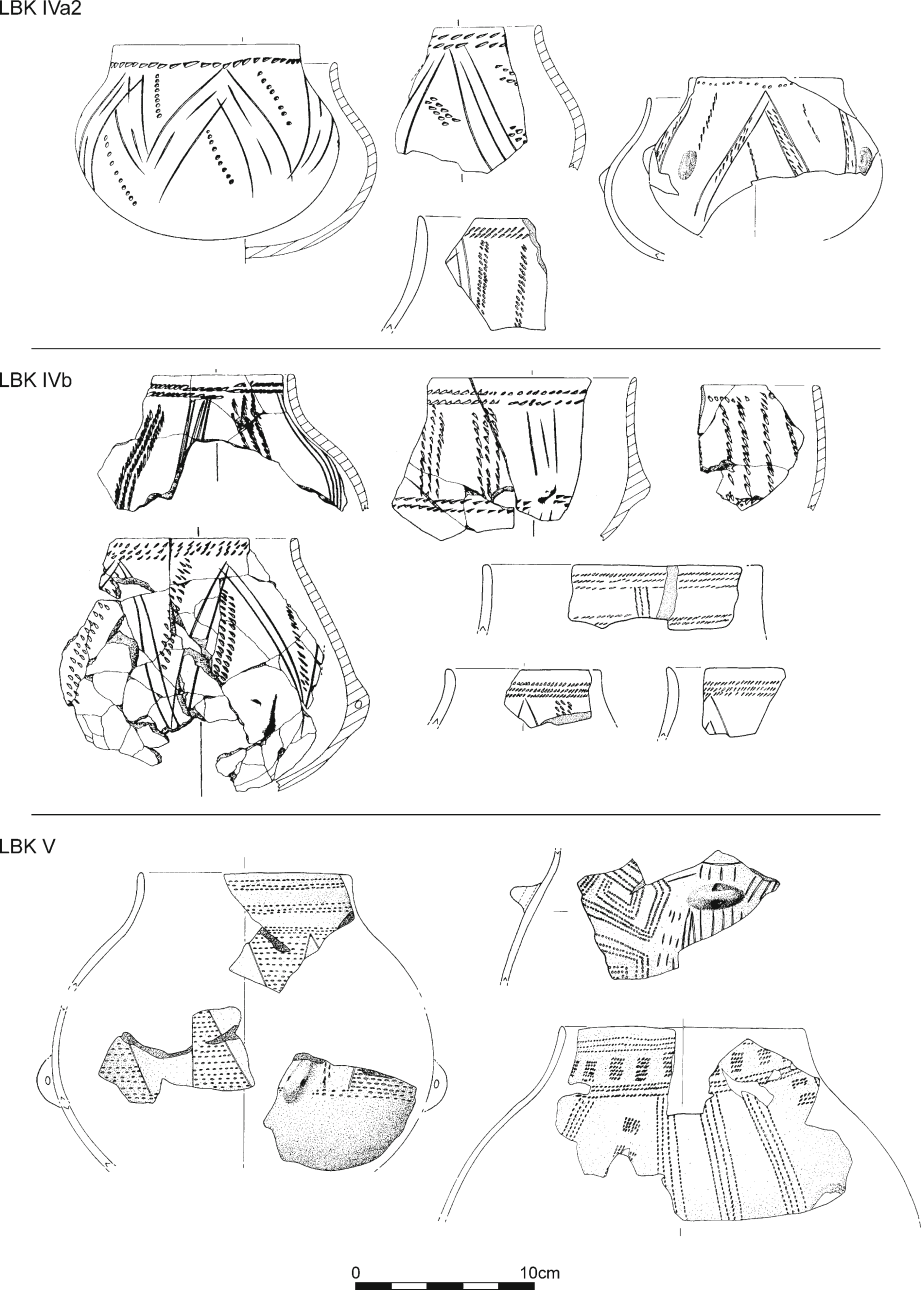
Typical decorative motifs for LBK pottery belonging to phases IVa2, IVb and V of the LBK seriation for Lower Alsace (Fig. 5; Electronic Supplementary Material Matrix 1)

The fifth phase, defined as LBK IVa2, is based on only seven assemblages. It has no new decorative motifs, but the motifs going back to phase III disappear. There are a very few motifs presaging LBK IVb. Angular decoration remains dominant. Rim decoration and segmented secondary motifs are formed by one or two rows of impressions (Fig. 7).

The sixth phase, using 20 assemblages, defines developed late LBK or LBK IVb. There are a dozen new decorative motifs, most typically rim decoration and segments of three parallel rows of impressions. Angular decoration remains dominant, and within that, the appearance of T-shapes and ladder motifs is noteworthy (Fig. 7).

The last phase, based on 11 pits, defines the final LBK or LBK V. Angular decoration disappears, to be replaced by orthogonal T-shaped or horizontal ladder motifs, formed by wide impressed vertical lines. Rim decoration develops further, with up to six rows of impressions, often with ‘metope’ layout.

## Modelling the Chronology of the LBK Ceramic Sequence in Lower Alsace

**Fig. 8:**
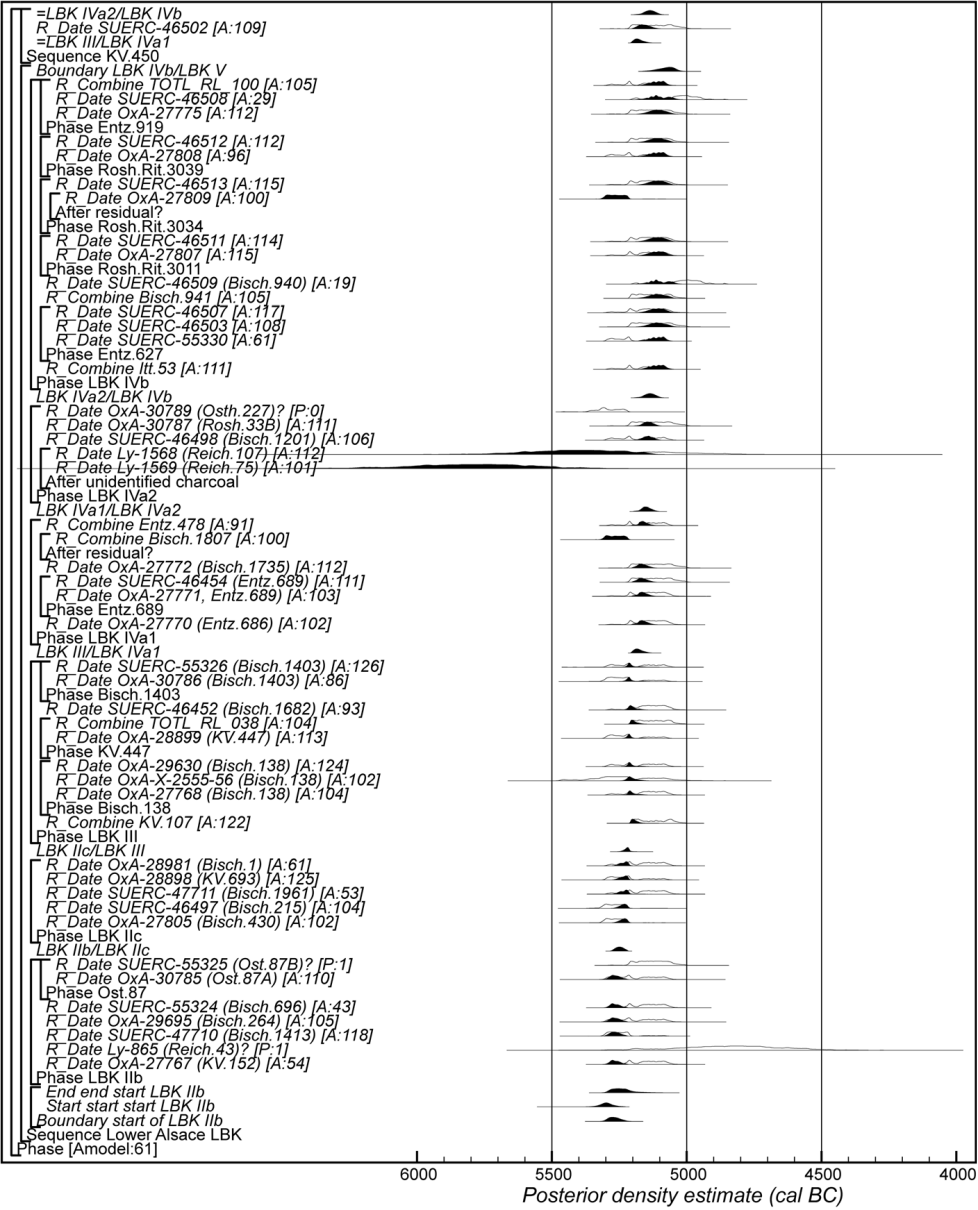
Probability distributions of radiocarbon dates from the sequence of LBK ceramics in Lower Alsace suggested by correspondence analysis (Fig. 5; Electronic Supplementary Material Matrix 1). Each distribution represents the relative probability that an event occurs at a particular time. For each date, two distributions are plotted: one in outline, which is the result of simple radiocarbon calibration, and a solid one, based on the chronological model used. Distributions other than those relating to particular samples correspond to aspects of the model. For example, the distribution ‘*start start LBK IIb*’ is the estimated date when the very first LBK ceramics appeared in Lower Alsace. Measurements followed by a *question mark* and shown in outline have been excluded from the model for reasons explained in the text and are simple calibrated dates (Stuiver and Reimer 1993). The *large square brackets down the left-hand side*, *along with the OxCal keywords*, define the overall model exactly

The chronological model for the LBK sequence in Lower Alsace is shown in Fig. 8. This model has good overall agreement (Amodel: 70; Bronk Ramsey 2009a, pp. 356–7). This means that the radiocarbon dates are compatible with the seriation shown in Fig. 5.

We have allowed for a gradual appearance of LBK ceramics in Lower Alsace (using a flexible trapezium distribution; Lee and Bronk Ramsey 2012), since we would like to estimate the duration of this process. In contrast, the clear boundaries between the succeeding ceramic phases shown in the seriation (Fig. 5) mean that we have modelled these transitions as effectively instantaneous.

Six assemblages have been dated from phase LBK IIb, with two radiocarbon dates excluded from the modelling. Ly-865 appears to be anomalously recent for technical reasons, as it probably contained exogenous later contaminants that were not fully removed by the pretreatment protocols used in the early 1970s. SUERC-55325 from Osthouse Kleinfeld pit 87 also appears to be slightly too recent (although it is statistically consistent with the other articulating bone sample from this pit). Either this is a statistical outlier or the sample came from an unrecorded later feature which cut through this pit.

**Fig. 9:**
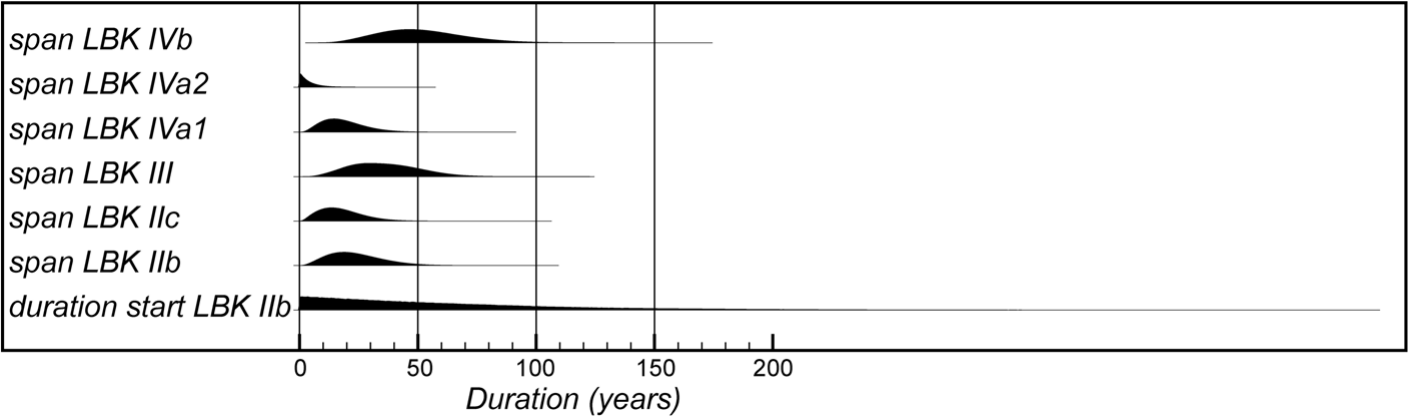
Posterior density estimates for the durations of the LBK ceramic phases and the introduction of LBK pottery into Lower Alsace (derived from the model shown in Fig. 8)

The model suggests that the first LBK pottery appeared in Lower Alsace in *5355–5240 cal BC* (*95% probability*; *start start LBK IIb*; Fig. 8), probably in *5325–5270 cal BC* (*68% probability*). The end of this beginning occurred in *5305–5145 cal BC* (*95% probability*; *end start LBK IIb*; Fig. 8), probably in *5285–5215 cal BC* (*68% probability*). Overall, this process took *1–170 years* (*95% probability*; *duration start LBK IIb*; Fig. 9), probably *1–75 years* (*68% probability*). Probably, the LBK appeared in Lower Alsace over the course of a few generations in the first half of the fifty-third century cal BC. LBK IIb ceramics ended and LBK IIc ceramics appeared in *5280–5220 cal BC* (*95% probability*; *LBK IIb/LBK IIc*; Fig. 8), probably in *5265–5230 cal BC* (*68% probability*). LBK IIb ceramics were in use for *1–50 years* (*95% probability*; s*pan LBK IIb*; Fig. 9), probably for *5–35 years* (*68% probability*). LBK IIb ceramics seem to have been used by the first generation of Neolithic Danubian settlers in Lower Alsace in the first half of the fifty-third century cal BC.

Five assemblages from phase IIc of the LBK ceramic seriation have radiocarbon dates. LBK IIc ceramics also appear to have had a limited currency in Lower Alsace. They ended and the succeeding LBK phase III began in *5250–5200 cal BC* (*95% probability*; *LBK IIc/III*; Fig. 8), probably in *5235–5210 cal BC* (*68% probability*). They were thus current for a period of *1–40 years* (*95% probability*; s*pan LBK IIc*; Fig. 9), probably for *1–25 years* (*68% probability).* LBK IIc encompasses the ceramics used by the Neolithic population of Lower Alsace in the third quarter of the fifty-third century cal BC.

Five assemblages from phase III of the LBK pottery seriation have radiocarbon dates. The model suggests that this phase of ceramic style went out of use and the succeeding LBK phase IVa1 commenced in *5210–5145 cal BC* (*95% probability*; *LBK III/IVa1*; Fig. 8), probably in *5200–5165 cal BC* (*68% probability*). LBK III ceramics were thus in use for *5–65 years* (*95% probability*; *span LBK III*; Fig. 9), probably for *15–50 years* (*68% probability*). The LBK III style was used in Alsace by the two generations who lived either side of 5200 cal BC.

Five assemblages included in phase IVa1 of the LBK pottery seriation have radiocarbon dates. One sample, articulating animal bone from Bischoffsheim pit 1807, appears to be reworked and has been incorporated in the model as a *terminus post quem*. LBK IVa1 ended and IVa2 began in *5185–5110 cal BC* (*95% probability*; *LBK IVa1/IVa2*; Fig. 8), probably in *5170–5130 cal BC* (*68% probability*). It was in use for a period of *1–40 years* (*95% probability*; *span LBK IVa1*; Fig. 9), probably for *5–25 years* (*68% probability*). LBK IVa1 pottery was used in Lower Alsace by the generation who lived around 5160 cal BC.

Five assemblages from phase IVa2 of the LBK ceramic seriation have been dated. Two of these dates are on unidentified charcoal, so simply provide *termini post quos* for these assemblages (Ly-1568*–*9). The grave 227 at Osthouse Kleinfeld contains a vessel that is clearly of IVa2 type. The radiocarbon date (OxA-30789) from this burial, however, is clearly too early for its position in the model. Either this is a statistical outlier or there is a dietary offset in the human skeleton (see below). This measurement has therefore been excluded from the analysis. On this basis LBK IVa2 ceramics ended and LBK IVb ceramics began in *5180–5095 cal BC* (*95% probability*; *LBK IVa2/IVb*; Fig. 8), probably in *5160–5115 cal BC* (*68% probability*). LBK IVa2 appears to have been an extremely brief style. It was in use for only *1–15 years* (*95% probability*; *span LBK IVa2*; Fig. 9), probably for *1–10 years* (*68% probability*). This therefore represents ceramics used by people living in Lower Alsace around 5140 cal BC.

Eight assemblages from phase IVb of the LBK pottery seriation have radiocarbon dates. One sample, a pair of sheep/goat mandibles thought to come from the same animal from Rosheim Rittergass, pit 3034 (OxA-27809), seems to be residual and has been modelled as a *terminus post quem* for this feature. LBK IVb ceramics ended and the succeeding LBK V ceramics began in *5145–5020 cal BC* (*95% probability*; *LBK IVb/V*; Fig. 8), probably in *5100–5040 cal BC* (*68% probability*). LBK IVb ceramics were, therefore, in use for a period of *15–90 years* (*95% probability*; *span LBK IVb*; Fig. 9), probably for *30–70 years* (*68% probability*). LBK IVb style was in use for two or three generations around 5100 cal BC.

No datable material could be located from the 11 pits whose pottery comprises phase V of the LBK pottery seriation.

## Middle Neolithic Pottery from Lower Alsace: Types, Motifs and Sequence

The ceramic sequence in the upper Rhine valley in Lower Alsace is divided into seven successive typological stages. The Middle Neolithic sequence begins with Hinkelstein, the pottery of which is taken by some to mark an intrusive style (Spatz 1996; Jeunesse and Arbogast 1997). Then comes Grossgartach, followed by Planig-Friedberg and Rössen. The latter part of the sequence is marked by more differentiation, from Bischheim, through Bruebach-Oberbergen and BORS. The origins of the latter may lie in the Paris basin (Jeunesse et al. 2003; Lefranc et al. 2012), as with the Michelsberg culture, whose appearance in Lower Alsace brought to an end the long Danubian cultural tradition. This profound break is seen particularly clearly in the pottery, with decoration, once so abundant and so widely shared, becoming extremely rare.

The links between the decoration and forms of Hinkelstein, Grossgartach, Planig-Friedberg and Rössen pottery make it possible to include all four groups in a single correspondence analysis. Although this had been done recently for the southern part of the upper Rhine valley (Denaire 2009), a new correspondence analysis has been carried out for this paper, to include features from newly excavated finds. This is based entirely on decorative motifs. These include horizontal rows, usually in twos or threes, a little under the rims and around the greatest girth of pots; when there is a third row, it is placed between these two zones, and if a fourth, lower down.

**Fig. 10:**
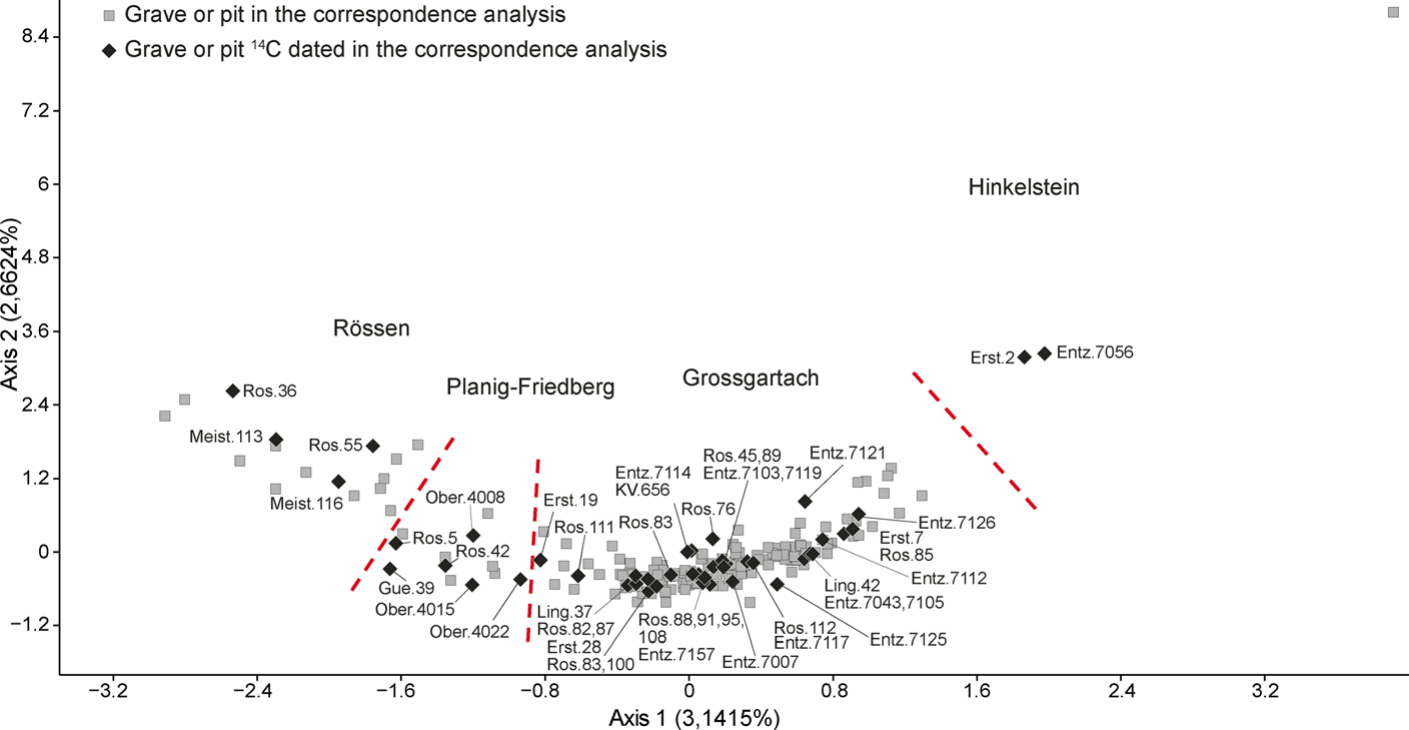
Correspondence analysis of Middle Neolithic ceramics in Lower Alsace

**Fig. 11:**
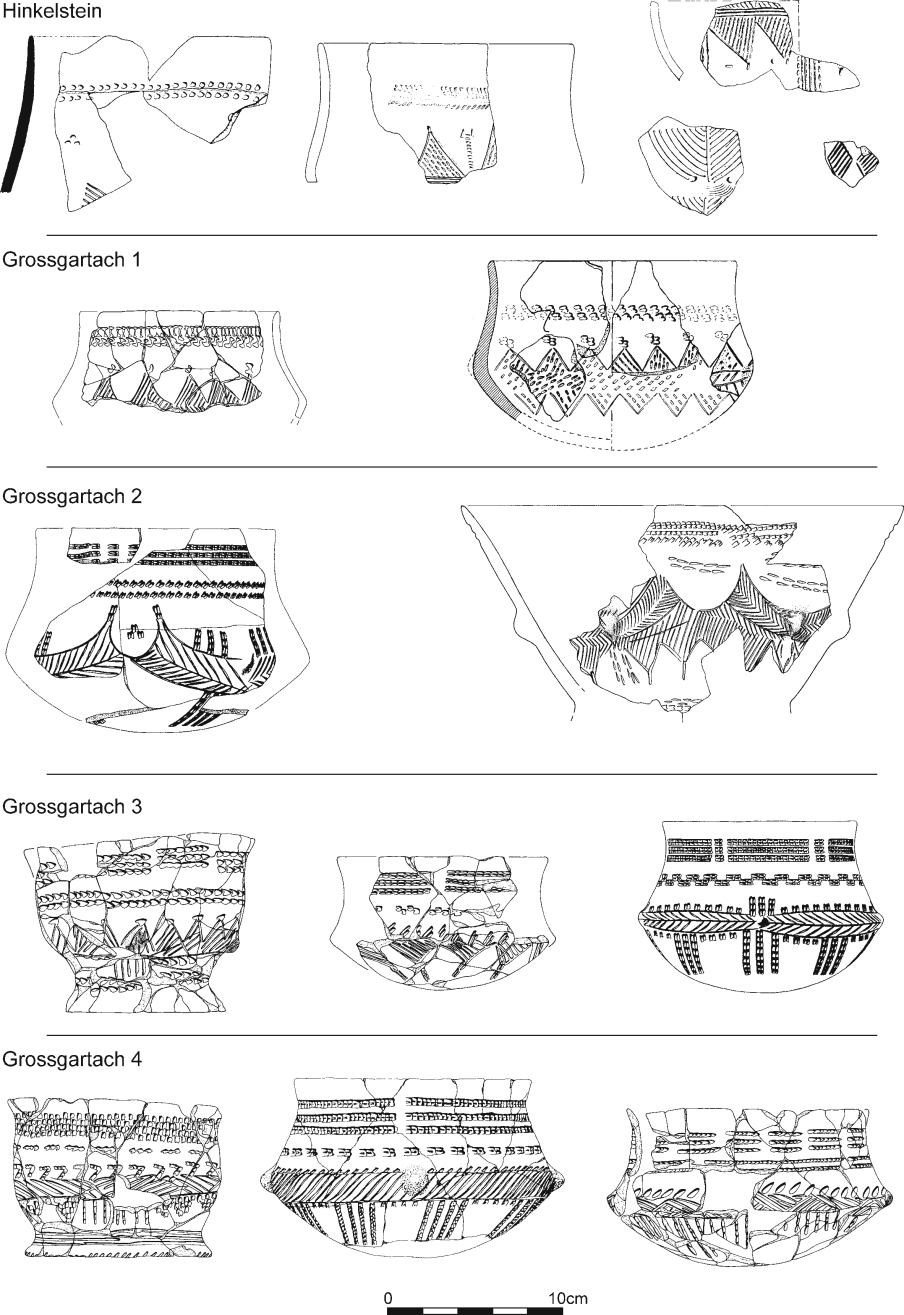
Typical decorative motifs for pottery belonging to the Hinkelstein and Grossgartach (1–4) phases of the Middle Neolithic seriation for Lower Alsace (Fig. 10; Electronic Supplementary Material Matrix 2)

The new seriation is based on 190 assemblages, predominantly Grossgartach, and 208 motifs from 27 sites (Fig. 10; Electronic Supplementary Material Matrix 2). On Fig. 10 can be seen, from right to left, the four phases, from Hinkelstein to Grossgartach and on to Planig-Friedberg and Rössen. Only three Hinkelstein assemblages have been included in the seriation, but these nonetheless include the principal characteristics of late Hinkelstein style, such as the use of comb impressions for rim decoration and incised lozenges and triangles on the body of pots (Fig. 11; Meier-Arendt 1975; Spatz 1996).

**Fig. 12:**
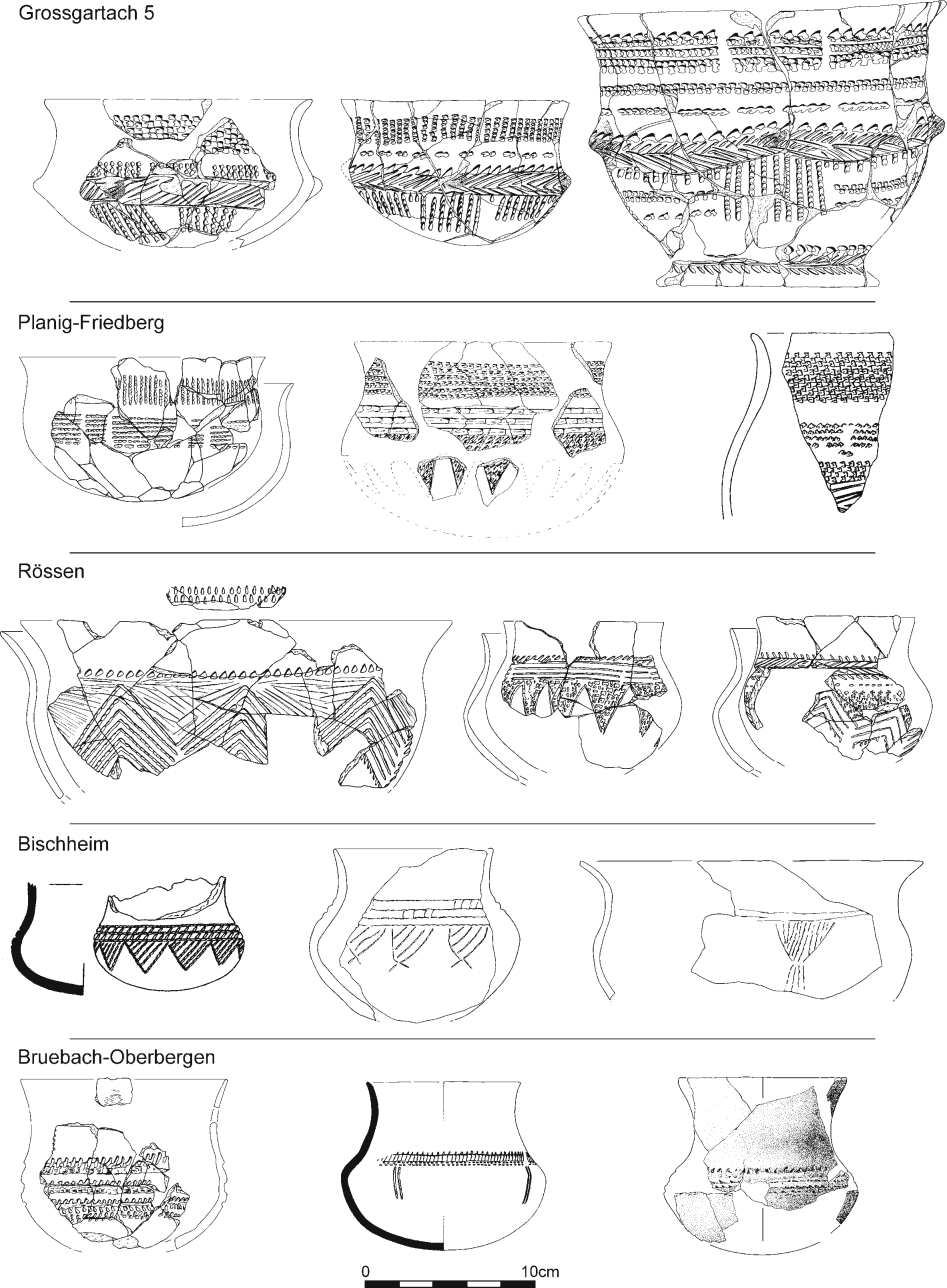
Typical decorative motifs for pottery belonging to the Grossgartach (5), Planig-Friedberg and Rössen phases of the Middle Neolithic seriation for Lower Alsace (Fig. 10; Electronic Supplementary Material Matrix 2) and Bischheim and Bruebach-Oberbergen phases

The five sub-groups previously defined for the Grossgartach (GG) style (Denaire 2009, pp. 174–257, Fig. 124) can be distinguished in the new seriation, though less clearly than before. GG 1 can be understood as transitional; friezes of opposed triangles link back to the Hinkelstein style (Fig. 11). GG 2 has ‘baroque’ decoration, combining friezes of hatched triangles with garland motifs. The absolutely typical Grossgartach double impressions belong here, both on the body of pots as well as on necks. Grooved rows of pointed impressions are omnipresent, the tool of the potter not being completely taken out between strokes, to produce the characteristic grooved effect (Fig. 11). GG 3 is stylistically very similar to GG 2. Rim decoration is more complex; there are fewer friezes with triangles and more hatched horizontal bands, and short vertical lines hanging below these bands (Fig. 11). GG 4 and 5 have increased horizontal zonation (Figs. 11 and 12), marked also by more rows of impressions below the rim.

Only 12 Planig-Friedberg assemblages could be included in the seriation. They fall clearly between Grossgartach and Rössen. There are more rows of impressions below the rim, and more extensive decoration on the belly of pots (Fig. 12).

The Rössen phase is slightly better represented in the seriation, by 16 assemblages. The most striking development is for decoration to be grouped closely on the middle of vessels and on their necks (Fig. 12). The decorative repertoire is more restricted than that of the Grossgartach style. It should be noted that only one phase of Rössen pottery has been recognised in Lower Alsace, while three have been defined in south-west and west Germany (Spatz 1996).

Bischheim and Bruebach-Oberbergen assemblages could not be included in the seriation because of their simple and homogenous decoration. The layout of decoration changes, with most pots having only one row, on the upper part of the belly. Bischheim potters preferred a horizontal band with a frieze of triangles, and those of Bruebach-Oberbergen fine bands executed with a spatula. In both these styles, some vessels had decoration restricted to a simple row of impressions linking lugs (Fig. 12).

**Fig. 13:**
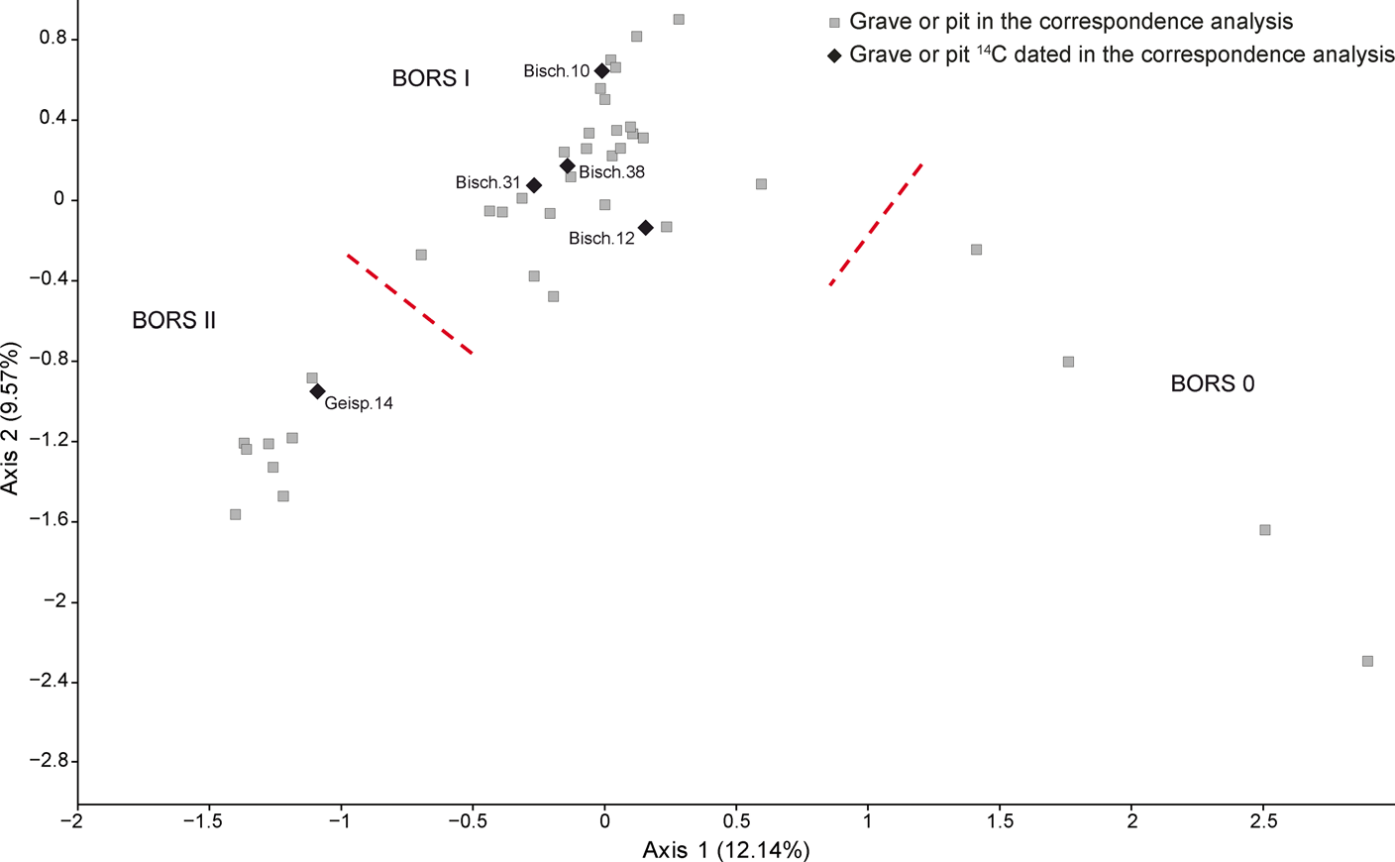
Correspondence analysis of BORS pottery in Lower Alsace

**Fig. 14:**
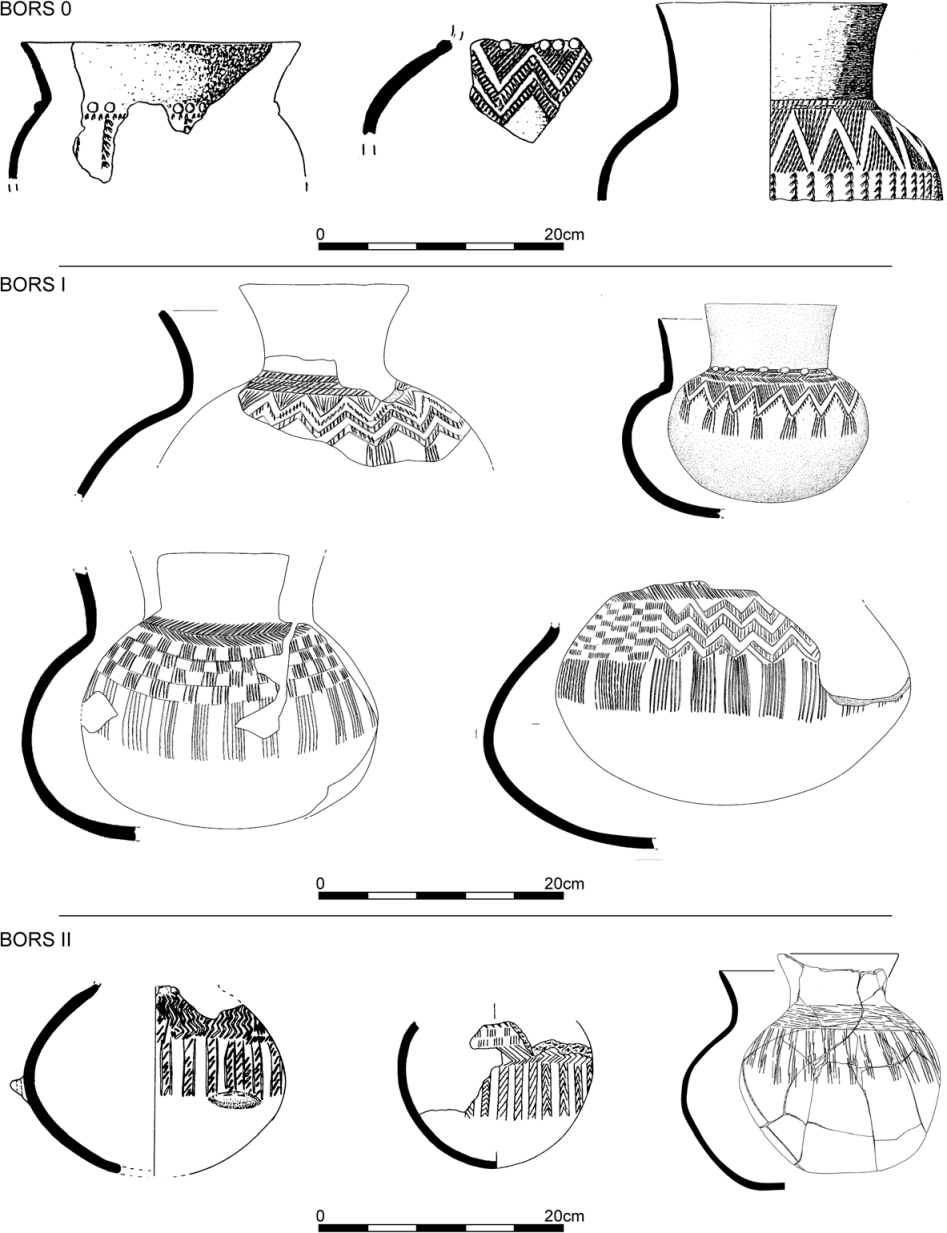
Typical decorative motifs for pottery belonging to the BORS 0, BORS I and BORS II phases of the BORS seriation for Lower Alsace (Fig. 13; Electronic Supplementary Material Matrix 3)

Assemblages under the BORS label signify a clean break with their Bruebach-Oberbergen predecessors (Jeunesse et al. 2003). Spatulated bands disappear, to be replaced by more exuberant and complex decorative schemes (Fig. 14). The recently discovered remarkable assemblages from Dambach-la-Ville (Croutsch et al. 2013) have enabled a fresh seriation of decorated BORS pottery based on 42 assemblages and 41 motifs from 11 sites, which has now produced three phases (Fig. 13; Electronic Supplementary Material Matrix 3). This is an ongoing work by Philippe Lefranc, replacing a previous two-phase scheme (Jeunesse et al. 2003). Phase 0 is characterised above all by angular borders (Fig. 14). Phase I represents the flowering of this style; angular borders and empty bands accompany rectangular chequerboard motifs and friezes of triangles, with short hanging lines. In phase II incision becomes more important than point-formed grooves, and the repertoire of motifs becomes restricted to chequerboards and T-shaped motifs. No datable material has yet been recovered from Phase 0 contexts, so this paper models the chronology of BORS I and II only. We should note, finally, the existence of closed assemblages containing both BORS II and Michelsberg II pots in Lower Alsace (Jeunesse et al. 2003; Meunier et al. 2003).

## Modelling the Chronology of the Middle Neolithic Ceramic Sequence in Lower Alsace

**Fig. 15:**
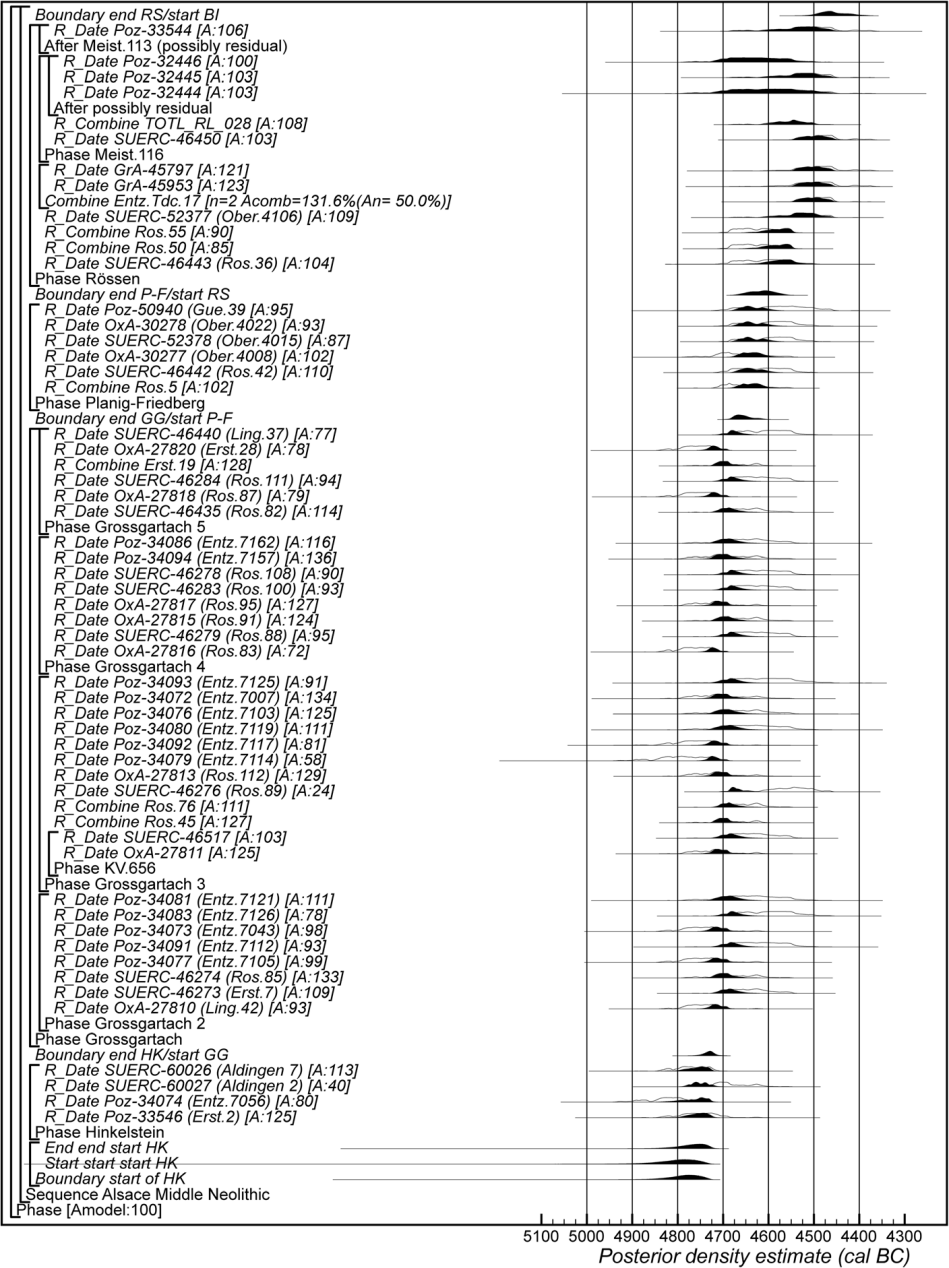
Probability distributions of radiocarbon dates from the sequence of Middle Neolithic ceramics in Lower Alsace suggested by correspondence analyses of the Hinkelstein–Rössen and BORS stages (Figs. 10 and 13; Electronic Supplementary Material Matrices 2 and 3) and the typology of the Bischheim and Brubach-Oberbergen stages: part 1 (Hinkelstein–Rössen). The format is identical to that of Fig. 8. The *large square brackets down the left-hand sides* of Figs. 15 and 16, *along with the OxCal keywords*, define the overall model exactly

**Fig. 16:**
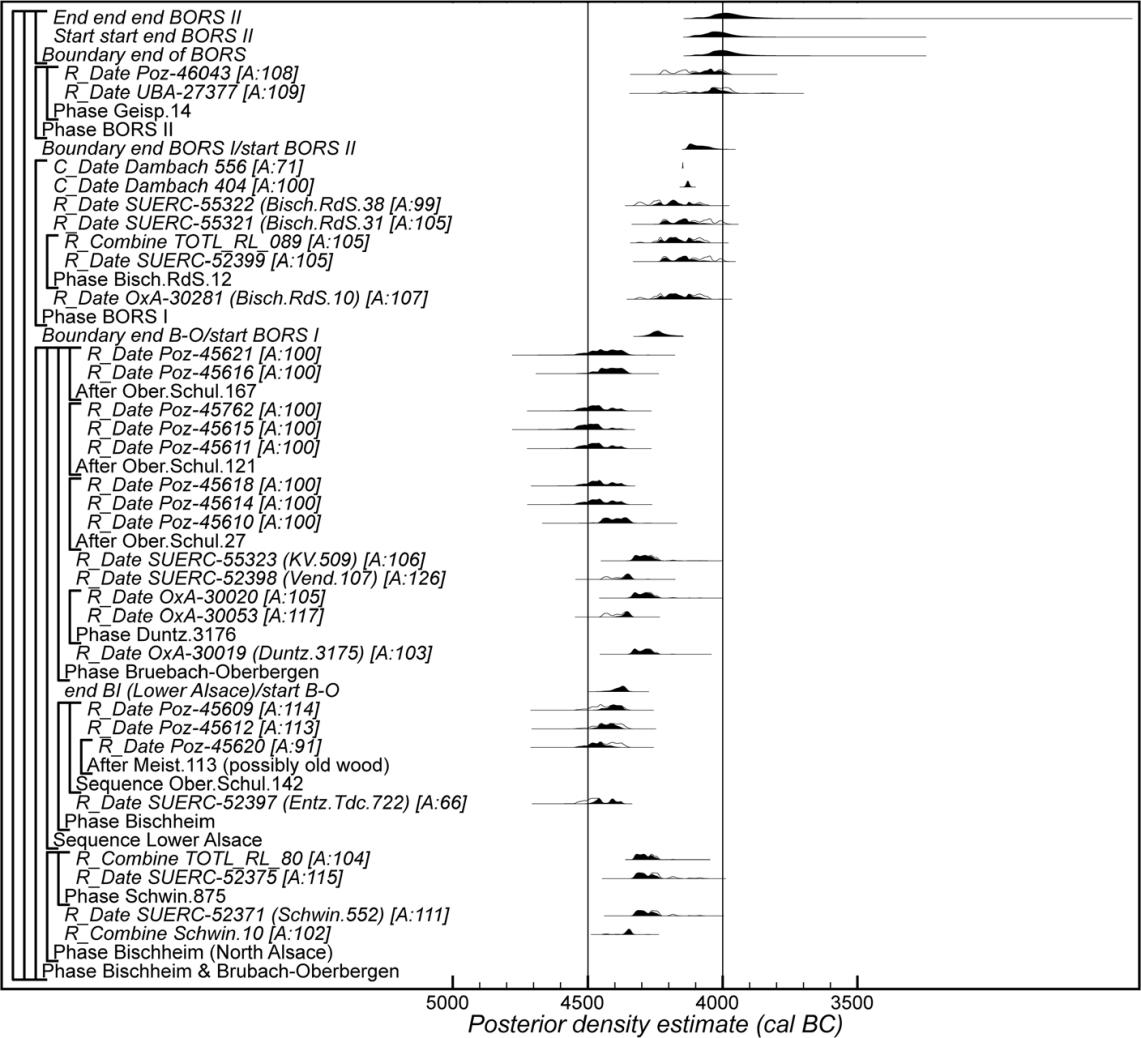
Probability distributions of radiocarbon dates from the sequence of Middle Neolithic ceramics in Lower Alsace suggested by correspondence analyses of the Hinkelstein–Rössen and BORS stages (Figs. 10 and 13; Electronic Supplementary Material Matrices 2 and 3) and the typology of the Bischheim and Brubach-Oberbergen stages: part 2 (Bischheim (BORS)). The format is identical to that of Fig. 8. The *large square brackets down the left-hand sides* of Figs. 15 and 16, *along with the OxCal keywords*, define the overall model exactly

The chronological model for the Middle Neolithic ceramic sequence is shown in Figs. 15 and 16. This model has good overall agreement (Amodel: 100). This means that the radiocarbon dates are compatible with the seriation of the Hinkelstein–Rössen ceramic motifs shown in Fig. 10, with the typological succession of ceramics from the later Middle Neolithic (allowing for the minor adjustment described below in the Bischheim/Bruebach-Oberbergen sequence), and with the seriation of BORS pottery shown in Fig. 13. The radiocarbon dates are also compatible with the limited amount of stratigraphy and tree-ring dates that are available.

We have allowed a gradual beginning to the Middle Neolithic ceramic sequence in Lower Alsace, since there is no continuity with the LBK style in this region. In contrast, the clear boundaries between the succeeding ceramic phases shown in the seriations (Figs. 10 and 13) mean that we have modelled these transitions as effectively instantaneous. We have, however, allowed the intensity of pottery production and use in different periods to have varied. Beyond the seriation, we have also modelled successive phases, because we have no evidence that these styles were in contemporary use. We have also allowed the end of the Danubian tradition of decorated ceramics to be gradual, since the succeeding Michelsberg phase has different origins and has undecorated pottery.

**Fig. 17:**
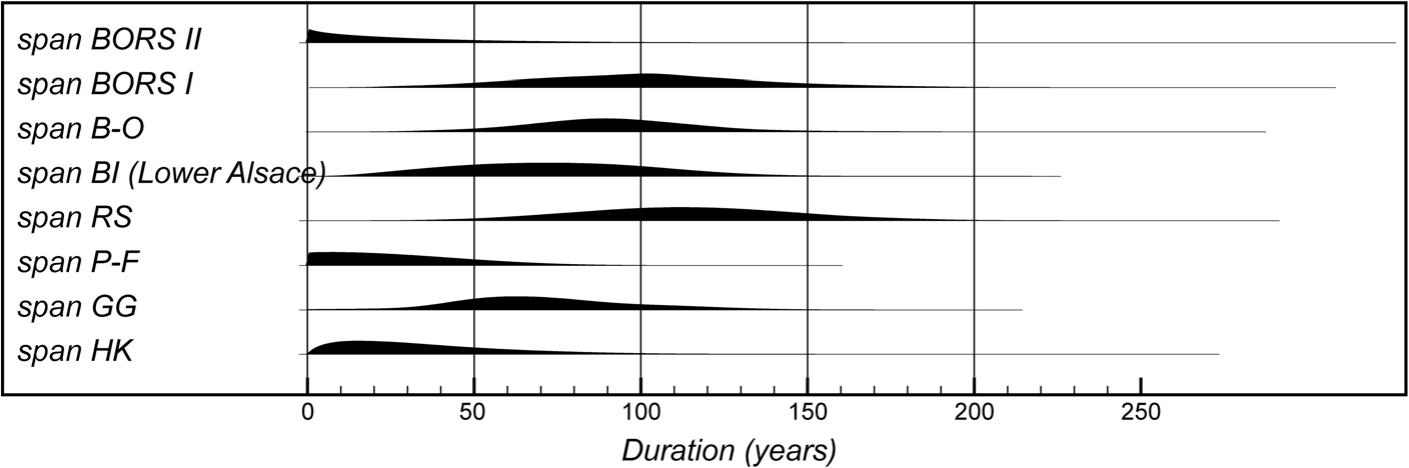
Posterior density estimates for the durations of the Middle Neolithic ceramic phases in Lower Alsace (derived from the model shown in Figs. 15 and 16)

The Middle Neolithic sequence commences with the Hinkelstein, from which just three assemblages could be included in the seriation, with only two graves providing datable material. These results provided unexpectedly late dating for the first Middle Neolithic activity in Lower Alsace, so we obtained further samples from the neighbouring Neckar valley region to assess whether these two dates from Lower Alsace were anomalous. These two dates on human burials from Remseck-Aldingen are included in the model, and are entirely compatible with dates on similar graves in Lower Alsace. This model suggests that Hinkelstein pottery made an initial appearance in this area in *4910–4725 cal BC* (*95% probability*; *start start HK*; Fig. 15), probably in *4835–4745 cal BC* (*68% probability*). It was fully established there by *4850–4715 cal BC* (*95% probability*; *end start HK*; Fig. 15), probably by *4795–4730 cal BC* (*68% probability*). The appearance of Hinkelstein ceramics in Alsace was swift: taking *1–115 years* (*95% probability*; *duration start HK*; distribution not shown), probably *1–40 years* (*68% probability*). The use of Hinkelstein pottery in Lower Alsace ended and it was replaced by the succeeding Grossgartach style in *4765–4705 cal BC* (*95% probability*; *end HK/start GG*; Fig. 15), probably in *4745–4720 cal BC* (*68% probability*). Overall, therefore, Hinkelstein ceramics were current in Lower Alsace for a period of *1–90 years* (*95% probability*; *span HK*; Fig. 17), probably for a period of *1–50 years* (*68% probability*). Hinkelstein ceramics were probably used in this area for one or two generations in the middle of the forty-eighth century cal BC.

Grossgartach pottery is much more common in Lower Alsace than the preceding Hinkelstein type, with 154 assemblages included in the seriation. Thirty-seven radiocarbon dates are available from 33 of these assemblages, from five sites. Of these dates, only one, that from Rosheim Rosenmeer grave 89 (SUERC-46276), has poor individual agreement (A: 25). The ceramics from the grave are clearly Grossgartach in style so perhaps this radiocarbon measurement is a slightly late statistical outlier. The transition to the succeeding Planig-Friedberg style occurred in *4690–4610 cal BC* (*95% probability*; *end GG/start P-F*; Fig. 15), probably in *4680–4640 cal BC* (*68% probability*). Grossgartach pottery was current in Lower Alsace for a period of *20–135 years* (*95% probability*; *span GG*; Fig. 17), probably for a period of *40–95 years* (*68% probability*). The intensity of activity in this period increases greatly from the preceding Hinkelstein period. Not only are there more ceramics but these come from a larger number of settlements and cemeteries, concentrated over perhaps three generations in the late forty-eighth and earlier forty-seventh centuries cal BC.

Attempts to incorporate the five sub-phases of the Grossgartach suggested by Denaire (2009, pp. 174–257) failed. These groupings appear to be robust (since they are apparent, albeit less clearly, in the revised seriation presented in Fig. 10), but they do not appear to represent a chronological sequence. This result is surprising, especially as it contrasts with a previous broad consensus on the stylistic development of the Grossgartach style; only the details of how to divide things up have differed from region to region (Spatz 1996; Biermann 1997; Eisenhauer 2002; Lönne 2003; Denaire 2009). Following the model presented here, alternative explanations have now to be found for contemporary variation, which we pursue in the ‘Discussion’ below.

Six assemblages from three sites have been dated from the subsequent Planig-Friedberg phase. These cover a period of *1–70 years* (*95% probability*; *span P-F*; Fig. 17), probably *1–40 years* (*68% probability*). Planig-Friedberg constitutes a transition between typical Grossgartach and typical Rössen assemblages. Fully formed Rössen assemblages emerged in *4670–4565 cal BC* (*95% probability*; *end P-F/start RS*; Fig. 15), probably in *4645–4585 cal BC* (*68% probability*).

Rössen ceramics form the final phase in the Middle Neolithic ceramic seriation (Fig. 10). Seventeen assemblages are included in the correspondence analysis of which four from two sites have radiocarbon dates. Three further Rössen assemblages have been dated, although these cannot be included in the seriation. The Rössen style ended in *4515–4395 cal BC* (*95% probability*; *end RS/start BI*; Fig. 15), probably in *4495–4430 cal BC* (*68% probability*). The style was current for a period of *45–185 years* (*95% probability*; *span RS*; Fig. 17), probably for *75–150 years* (*68% probability*), from the decades around 4600 cal BC to the middle of the forty-fifth century cal BC. The comparative scarcity of ceramics in this period probably does not reflect a lowering in the intensity of Neolithic activity in Lower Alsace; rather, changes in burial fashion mean that there are fewer large cemeteries, and disposal activities on settlements shift to smaller pits resulting in a less visible archaeology.

Given the good agreement between the radiocarbon dates and the Middle Neolithic seriation, we wanted to extend our dating programme into the later part of the Middle Neolithic in Lower Alsace. Unfortunately, it is not possible to extend the seriation, because Bischheim pottery is decorated less frequently, a limited number of motifs that were used in the Rössen period continued, and the decoration is more uniform in style. There is undoubtedly, however, typological continuity with the preceding Rössen phase and the lack of closed contexts containing both Rössen and Bischheim styles suggests that the transition from one to the other was swift.

Bruebach-Oberbergen decoration is composed of motifs that had previously been uncommon, combining them into a distinctive and homogenous new style (Fig. 12). It is difficult to determine whether there was a succession from Bischheim to Bruebach-Oberbergen or whether there was a period during which the two pot styles were contemporary. At the Gonvillars cave, in the north of Franche-Comté (Fig. 4), an unmixed Bruebach-Oberbergen layer overlies a deposit containing both Bischheim and Rössen sherds. A model which includes a simple sequence between Bischheim and Bruebach-Oberbergen has poor overall agreement (Amodel: 0). This is because the dates on paired and articulating bone from Schwindratzheim grave 10 and pit 875, which contain pottery of the Bischheim style, are clearly later than carbonised residues on Bruebach-Oberbergen sherds from Duntzenheim pit 3176 and the articulated skeleton in pit 107 from Vendenheim, which also included a Bruebach-Oberbergen decorated vessel. Schwindratzheim lies at the northern limit of the distribution of Bruebach-Oberbergen pottery in Lower Alsace, so we suggest that this site lies in an area where Bischheim ceramics continued in use, whereas further south in Lower Alsace, communities adopted the Bruebach-Oberbergen style. For this reason we have modelled separately a Bischheim-Bruebach-Oberbergen sequence in Lower Alsace, which is contemporary with the currency of the Bischheim style further north.

This model suggests that the use of Bischheim ceramics in Lower Alsace ended and the Bruebach-Oberbergen style was adopted there in *4435–4345 cal BC* (*95% probability*; *end BI (Lower Alsace)/start B-O*; Fig. 16), probably in *4400–4355 cal BC* (*68% probability*). Bischheim ceramics were therefore current in Lower Alsace for a period of *15–130 years* (*95% probability*; *span BI (Lower Alsace)*; Fig. 17), probably for *40–105 years* (*68% probability*). Basically this style was in use here for three of four generations covering most of the forty-fifth and the earlier part of the forty-fourth centuries cal BC.

Bruebach-Oberbergen pottery had been replaced by the succeeding BORS style by *4295–4165 cal BC* (*95% probability*; *end B-O/start BORS I*; Fig. 16), probably by *4270–4210* (*68% probability*). This ceramic style was thus current for a period approaching *30–150 years* (*95% probability*; *span B-O*; Fig. 17), probably for *65–120 years* (*68% probability*). These estimates should be regarded with some caution, since we have not been able to date any samples from the BORS 0 phase. We can suggest, however, that this cannot have been of long duration, as the results of the model shown in Figs. 15 and 16 are compatible with two imported Bruebach-Oberbergen pots found in the cultural layer at Egolzwil 3, Switzerland, which has a tree-ring date of 4282*–*74 BC (Siefert et al. 2013; Denaire et al. 2011). Bruebach-Oberbergen was probably thus a persistent style in use for four or five generations for much of the forty-fourth and forty-third centuries cal BC.

We have no evidence for when the Bischheim style went out of use north of the Zorn River. It may have ended with the transition to the BORS style (and is modelled thus here), but evidence for this must await further excavation.

The decorative motifs on BORS pottery do not derive from the preceding Bruebach-Oberbergen phase but rather developed from the variety of Bischheim ceramic style used in the Paris basin, in contact with the Chasséen group (Jeunesse et al. 2003). There is, however, internal sequence within the BORS in Lower Alsace (Fig. 13). No datable samples were recovered from phase 0. Thirty BORS I assemblages are included in the seriation, of which six have been dated. Eight BORS II assemblages are included in the seriation, but only one of these produced datable material. On the basis of this limited evidence, the model suggests that the transition from BORS I to BORS II occurred in *4140–4025 cal BC* (*95% probability*; *end BORS I/start BORS II*; Fig. 16), probably in *4130–4065* (*68% probability*). BORS I pottery was thus current in Lower Alsace for a period of *30–170 years* (*95% probability*; *span BORS I*; Fig. 17), probably for *60–135 years* (*68% probability*)*.* Again, this style endured for perhaps four or five generations, from the middle of the forty-third century cal BC to the decades around 4100 cal BC. It should be noted that the two tree-ring dates from Dambach (new data from C. Croutsch) are entirely compatible with our dating for this phase (4128 ± 5 BC from well 404, and 4147 BC from well 556; Fig. 16).

BORS II pottery is the final decorated Middle Neolithic style in the Danubian tradition found in Lower Alsace. It was replaced by Michelsberg II (Jeunesse et al. 2003). The beginning of the end came in *4130–3880 cal BC* (*95% probability*; *start end BORS II*; Fig. 16), probably in *4075–3965 cal BC* (*68% probability*). Such decorated ceramics had finally disappeared from Lower Alsace by *4115–3805 cal BC* (*95% probability*; *end end BORS II*; Fig. 16), probably by *4045–3920 cal BC* (*68% probability*). BORS II was current for *1–165 years* (*95% probability*; *span BORS II*; Fig. 17), probably for *1–55 years* (*68% probability*). Basically it seems to have been used through much of the forty-first century cal BC.

## Sensitivity Analyses: the Possibility of Diet-Induced Radiocarbon Offsets

You are what you eat. Diet-induced radiocarbon offsets can occur if a dated individual has taken up carbon from a reservoir not in equilibrium with the terrestrial biosphere (Lanting and van der Plicht 1998). If one of the reservoir sources has an inherent radiocarbon offset—for example if the dated individual consumed marine fish or freshwater fish from a depleted source—then the bone will take on some proportion of radiocarbon that is not in equilibrium with the atmosphere. This makes the radiocarbon age older than it would be if the individual had consumed a diet consisting of purely terrestrial resources. Such ages, if erroneously calibrated using a purely terrestrial calibration curve will produce anomalously early radiocarbon dates.

The most reliable method of checking for the presence of a reservoir offset in the human bone samples would be to date ‘perfect pairs’, in the sense of herbivore and omnivore bone from the same context, articulating, as specified above, to ensure that neither was redeposited, and to then compare the results. Unfortunately, none could be located in the archives. Only one human bone sample has been dated from the LBK seriation (Table 1; Fig. 8), but well over half the dated samples from the Middle Neolithic sequence were human bones (Table 2; Figs. 15 and 16). The radiocarbon dates on animal bone samples that are contained within this sequence, however, without exception have good individual agreement with the relative sequence provided by the seriations and typological analysis and the corpus of radiocarbon dates on human bone that have been calibrated with a fully terrestrial calibration curve (Figs. 15 and 16; e.g. KV.656 or Meist 116). This suggests that any reservoir effect that might be present in the human skeletons that have been radiocarbon dated in this study is unlikely to affect the chronologies presented substantively.

Although the good agreement between the radiocarbon dates on the human bone and on other sample types in the Middle Neolithic sequence suggests that dietary reservoir effects in Neolithic human bone from this region were probably not widespread, this does not mean that particular individuals might not have consumed a larger component of freshwater resources. For this reason, source proportional dietary modelling was undertaken on the basis of carbon and nitrogen stable isotopic values, so that mixed-source calibration models could be constructed that would account for any potential reservoir affects in particular individuals.

The first step in the analysis is to estimate the proportions of different food sources in the diets of the dated individuals.

### Dietary Analysis of Human Remains

**Fig. 18:**
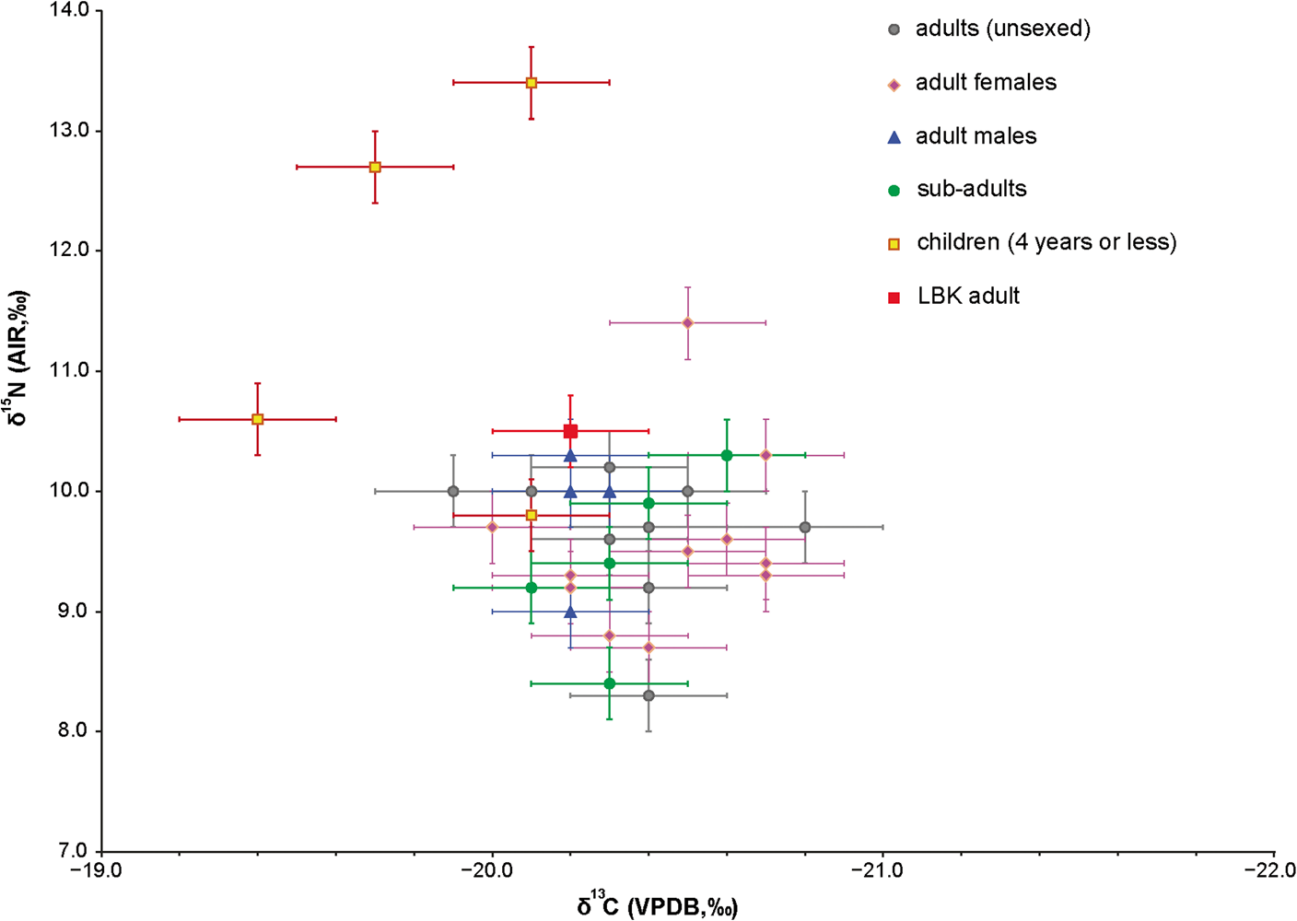
δ^13^C and δ^15^N values for human skeletons from Lower Alsace (*error bars* at 1σ)

The δ^13^C and δ^15^N values for the Middle Neolithic Rhineland population are from 30 adult and sub-adult humans, plus four children aged 4 years and under (Fig. 18). Mean isotopic values for all unsexed and sexed adults (*n* = 25) are −20.6 ± 0.2‰ for δ^13^C and +9.6 ± 0.3‰ for δ^15^N. Mean isotopic values for older children and sub-adults (ranging from 3 to 6 years to 15–19 years; *n* = 5) are −20.3 ± 0.2‰ for δ^13^C and +9.4 ± 0.3 for δ^15^N.

We examined the isotopic differences of infants relative to adults and sub-adults, as infant breast feeding and weaning onto cereal gruels should produce notable isotopic differences relative to other age cohorts. There were only four infants (3–5 months to 1–4 years; *n* = 4). As this population is small and is not normally distributed, a Mann-Whitney test was used to determine that the median δ^15^N value and standard deviation for the infants. The value of +11.6 ± 1.7‰ was significantly enriched over that of adult females (+9.6 ± 0.7‰; *P* = 0.0152) and adults with no sex determined (+9.6 ± 0.6‰; *P* = 0.0360). Infants were found to be not quite significantly enriched over sub-adults (+9.4 ± 0.7‰; *P* = 0.0635).

There is only one LBK human, an adult male approximately 20–39 years old, with isotopic values of −20.2 ± 0.2‰ for δ^13^C and +10.5 ± 0.3‰ for δ^15^N (Fig. 18).

We now turn to the dietary modelling for the population, and we will also use a special modelling treatment for infants who may have been between full breast feeding and weaning.

### FRUITS Modelling of Dietary Proportions in Diets of Dated Individuals

Diet re-construction for the dated humans from the upper Rhine utilised the Bayesian mixing model Food Re-construction Using Isotopic Transferred Signals (FRUITS) vβ2.0 (Fernandes et al. 2014). FRUITS employs the isotopic averages of possible food sources and allows the user to define isotopic offsets between diet and consumer, as well as the expected weighting and concentration of food sources. Prior information to constrain the calculations of the stable isotope mixing model can also be added. FRUITS then produces estimates of the mean percentage (and standard deviation) for each of the possible food sources making up the diet for each given consumer.

Weighted means of replicate isotopic values were used in the dietary modelling. The FRUITS results were produced from two diet proxies (δ^13^C and δ^15^N), and used the following food source data and assumptions in the model. The diet-to-consumer offsets were 4.8 ± 0.2‰ for δ^13^C (Fernandes et al. 2014) and 6.0 ± 0.5‰ for δ^15^N (O’Connell et al. 2012). The averages and the standard deviation of analytical error from δ^13^C and δ^15^N analyses of three possible food sources were used, with the weight and concentration of each of the three diet sources set at 100%. Crop values came from archaeobotanical samples of emmer and naked wheat, einkorn, barley and peas (Styring et al. in 2016) from late Neolithic sites at Hornstaad-Hörnle (*n* = 1342) and Sipplingen (*n* = 356). Terrestrial herbivore values (*n* = 60) came from faunal specimens (red deer, goat, cattle and pigs) analysed for this project from the LBK and Middle Neolithic sites.

Freshwater fish (pike and carp) are from archaeological fish data (*n* = 4) from the LBK settlement at Herxheim and the Middle Neolithic cemetery at Trebur (Durrwachter et al. 2006, Table 1). In the absence of obvious fish remains from the dated sites (but there was no systematic sieving that might retrieve such remains), the prior information used was that terrestrial herbivores probably provided a higher proportion of dietary protein than freshwater fish, and that crops also probably contributed more to the diet than freshwater fish. The mean values for the food sources used in our FRUITS diet proportion modelling are given in Table 3.

**Table 3:** Mean isotopic values for food sources used for the FRUITS modelling of adults and sub-adults and children under 4 years of age

Food source	δ^13^C (‰)	δ^15^N (‰)
Terrestrial herbivores (adults and sub-adults)	−21.2 ± 0.9	8.2 ± 1.3
Freshwater fish (adults and sub-adults)	−21.8 ± 0.9	8.9 ± 1.1
Crops (adults and sub-adults and children under 4 years of age)	−26.1 ± 0.9	5.2 ± 0.8
Breast milk (children under 4 years of age)	−20.4 ± 0.2	9.5 ± 0.7

Errors are the standard deviation on the mean values for each food source

Nursing- and weaning-age children present different considerations for diet modelling from adults and sub-adults. Enriched δ^15^N values for these subjects would be associated with breast feeding (Jay et al. 2008; Fuller et al. 2006) rather than fish or other higher protein foods, and the gradual introduction of solids which were commonly cereal gruels (Fildes 1986). There are no published values estimating the isotopic values of breast milk. The isotopic values of breast milk, however, would reflect the diet of the nursing mother, as breast milk is essentially the lactating mother’s tissue (Song 2004, 125).

We first created an estimated breast milk isotopic signature for modelling nursing/weaning children, using the mean isotopic value for the 12 female adult skeletons. The proxy baseline isotopic values for ‘breast milk’ used the mean isotopic value and standard deviation for the 12 female adult skeletons of −20.4 ± 0.3‰ for δ^13^C and 9.6 ± 0.7‰ for δ^15^N. An offset factor (the consumer’s trophic enrichment of the metabolised food) was then determined. The isotopic difference of our group of infants over female adults is +0.6‰ for δ^13^C and +2.0‰ for δ^15^N. While this could be used as an offset factor, we probably do not have actual pairs of mothers and their children, and so we chose to set the FRUITS offsets for the infants at +1.0 ± 0.5‰ for δ^13^C and +3.0 ± 0.5‰ for δ^15^N, following enrichment factors noted between mothers and nursing infants in actual paired mother–child studies (Fuller et al. 2006; Katzenberg et al. 1996; White and Schwartz 1994). The mean isotopic values for food sources used in the FRUITS modelling of infants are given in Table 3.

**Fig. 19:**
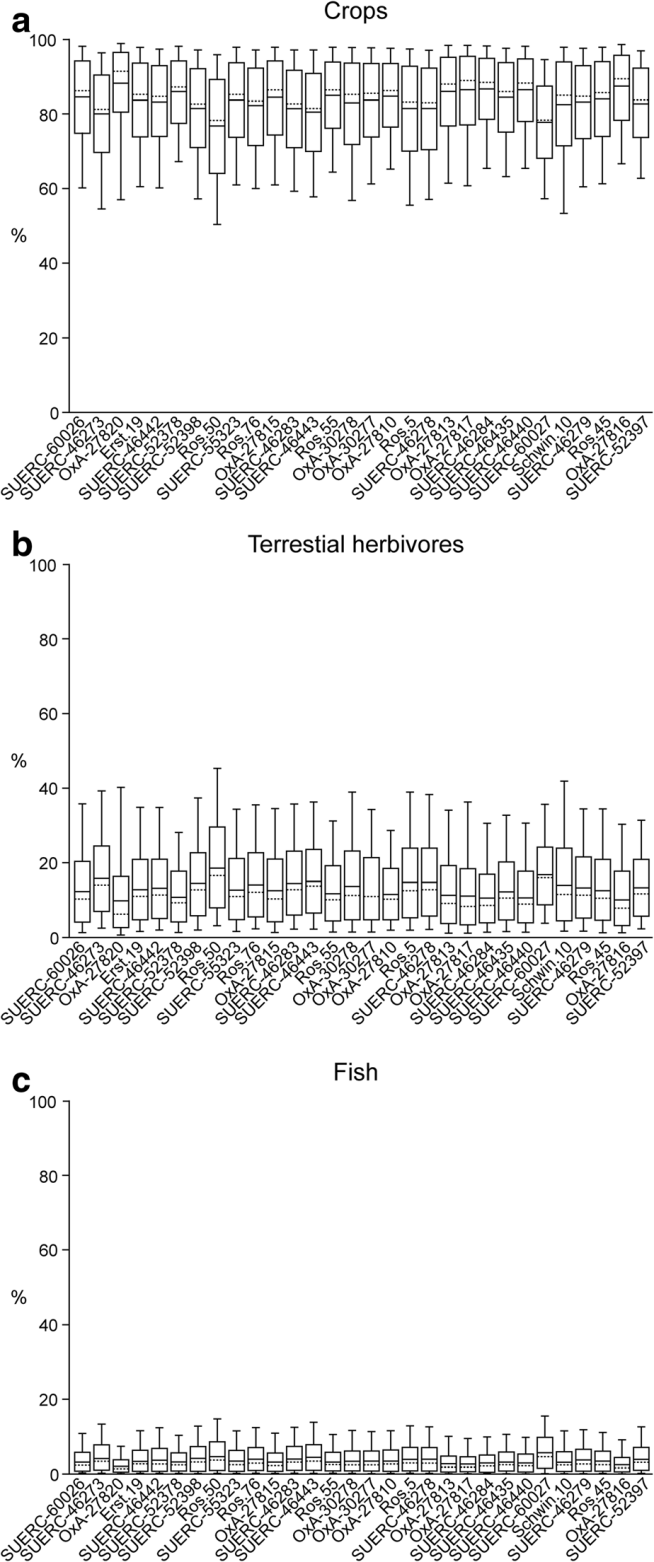
Box plots of the FRUITS estimations for each of the three diet sources, **a** crops, **b** terrestrial herbivores and **c** fish, for the adult and sub-adult human skeletons that have measured stable isotopic values (Tables 1, 2 and 4). The *boxes* provide a 68% confidence interval while the *whiskers* provide a 95% confidence interval. The *horizontal continuous line* indicates the average while the *horizontal discontinuous line* indicates the median (50th percentile). These box plots illustrate that crops are estimated to be the largest component of human diets in Lower Alsace at this period

The dietary estimates of the mean percentage (and standard deviation) for each of the possible food sources for the adult and sub-adult skeletons are shown in Table 4 (and see Fig. 19). Adult and sub-adult diet proportions show a substantial reliance on crops. Adults have a mean 79.8 ± 13.8% estimated crops, with a minimum 68.7% and maximum 89.1%. Terrestrial herbivores are a far smaller part of overall diets, with mean diet proportions of 15.9 ± 12.5%, and a minimum of 8.5% and maximum of 23.9%. Sub-adults are not significantly different, with mean estimated crops of 82.0 ± 13.1%. The FRUITS estimation of fish in the adult and sub-adult diets indicates little reliance on riverine food sources. Adult mean estimated fish is 4.2 ± 3.8%, and for sub-adults it is 3.7 ± 3.4%.

**Table 4:** FRUITS (Fernandes *et al*. 2014) estimates of diet proportions from the three food groups for the human skeletons dated as part of this study (weighted averages of replicate δ^13^C and δ^15^N analyses measured by isotope ratio mass spectrometry; see Tables 1 and 2)

Laboratory number	Burial	δ^13^C (‰)	δ^15^N (‰)	FRUITS diet proportion estimates		
				Terrestrial herbivore	Crops	Fish
LBK burial						
OxA-30789	Osth.227	−20.2 ± 0.2	10.5 ± 0.3	17.3 ± 9.0%	77.7 ± 11.0%	5.0 ± 4.0%
Middle Neolithic burials						
SUERC-60027	Aldingen 2	−20.1 ± 0.3	9.2 ± 0.3	14.8 ± 14.1%	81.7 ± 15.1%	3.5 ± 3.3%
SUERC-60026	Aldingen 7	−20.4 ± 0.3	9.2 ± 0.4	13.8 ± 11.0%	82.4 ± 12.5%	3.8 ± 3.5%
SUERC-52397	Entz.Tdc.722	−20.6 ± 0.2	10.3 ± 0.3	13.2 ± 7.7%	82.8 ± 9.1%	3.9 ± 3.4%
OxA-27819/SUERC-46436	Erst.19	−20.4 ± 0.14	8.3 ± 0.21	8.5 ± 7.8%	89.1 ± 8.7%	2.4 ± 2.3%
OxA-27820	Erst.28	−20.4 ± 0.2	9.7 ± 0.3	15.6 ± 11.5%	80.3 ± 13.0%	4.1 ± 3.8%
SUERC-46273	Erst.7	−20.1 ± 0.2	10.0 ± 0.3	18.0 ± 13.6%	77.2 ± 15.2%	4.8 ± 4.3%
SUERC-55323	KV.509	−20.3 ± 0.2	9.6 ± 0.3	14.9 ± 12.0%	81.2 ± 13.4%	4.0 ± 3.7%
SUERC-46440	Ling.37	−20.7 ± 0.2	9.4 ± 0.3	13.1 ± 10.0%	83.3 ± 11.3%	3.7 ± 3.3%
OxA-27810	Ling.42	−20.1 ± 0.2	9.8 ± 0.3	18.3 ± 14.7%	77.3 ± 16.0%	4.4 ± 4.0%
OxA-30277	Ober.4008	−20.2 ± 0.2	9.3 ± 0.3	13.4 ± 10.3%	82.9 ± 11.5%	3.6 ± 3.3%
SUERC-52378	Ober.4015	−20.8 ± 0.2	9.7 ± 0.3	15.4 ± 12.1%	80.5 ± 13.6%	4.1 ± 3.8%
OxA-30278	Ober.4022	−20.2 ± 0.2	9.2 ± 0.3	14.6 ± 13.8%	81.9 ± 14.9%	3.5 ± 3.3%
SUERC-46283	Ros.100	−20.2 ± 0.2	10.0 ± 0.3	20.4 ± 17.2%	74.8 ± 18.5%	4.9 ± 4.4%
SUERC-46278	Ros.108	−20.3 ± 0.2	8.8 ± 0.3	10.8 ± 9.0%	86.3 ± 10.1%	3.0 ± 2.7%
SUERC-46284	Ros.111	−20.7 ± 0.2	9.3 ± 0.3	13.1 ± 11.1%	83.4 ± 12.2%	3.6 ± 3.3%
OxA-27813	Ros.112	−20.0 ± 0.2	9.7 ± 0.3	16.1 ± 12.6%	79.6 ± 13.9%	4.2 ± 3.8%
SUERC-46443	Ros.36	−20.6 ± 0.2	9.6 ± 0.3	16.9 ± 14.4%	78.9 ± 15.7%	4.2 ± 3.9%
SUERC-46442	Ros.42	−20.5 ± 0.3	10.0 ± 0.3	17.0 ± 13.3%	78.3 ± 14.8%	4.7 ± 4.1%
Oxa-27814/SUERC-46277	Ros.45	−20.4 ± 0.14	9.9 ± 0.21	18.3 ± 15.1%	77.2 ± 16.5%	4.5 ± 4.2%
OxA-27821/SUERC-46441	Ros.5	−20.7 ± 0.14	10.3 ± 0.21	19.8 ± 14.0%	74.9 ± 15.6%	5.3 ± 4.6%
OxA-27972/OxA-27973/SUERC-46444	Ros.50	−20.3 ± 0.12	10.2 ± 0.17	19.8 ± 15.7%	75.2 ± 16.9%	5.0 ± 4.3%
OxA-27822/SUERC-46445	Ros.55	−20.2 ± 0.14	10.3 ± 0.21	21.7 ± 16.3%	73.0 ± 17.6%	5.3 ± 4.7%
SUERC-46275/OxA-27812	Ros.76	−20.3 ± 0.14	10.0 ± 0.21	17.91 ± 11.6%	77.2 ± 13.0%	4.9 ± 4.1%
SUERC-46435	Ros.82	−20.5 ± 0.2	9.5 ± 0.3	14.6 ± 12.4%	81.6 ± 13.7%	3.8 ± 3.6%
OxA-27816	Ros.83	−20.3 ± 0.2	8.4 ± 0.3	10.4 ± 9.8%	86.9 ± 10.9%	2.7 ± 2.6%
SUERC-46279	Ros.88	−20.3 ± 0.2	9.4 ± 0.3	14.6 ± 12.5%	81.6 ± 13.7%	3.8 ± 3.4%
OxA-27815	Ros.91	−20.2 ± 0.2	9.0 ± 0.3	11.4 ± 10.5%	85.6 ± 11.5%	3.1 ± 2.8%
OxA-27817	Ros.95	−20.4 ± 0.2	8.7 ± 0.3	10.2 ± 8.2%	87.0 ± 9.2%	2.9 ± 2.6%
OxA-30279/SUERC-52370	Schwin.10	−20.5 ± 0.14	11.4 ± 0.21	23.9 ± 13.9%	68.7 ± 15.9%	7.4 ± 5.8%
SUERC-52398	Vend.107	−19.9 ± 0.2	10.0 ± 0.3	19.5 ± 16.0%	75.6 ± 17.3%	4.9 ± 4.3%

Excavation reports for individual sites provide no evidence for fish bone or the consumption of freshwater foods, although several bone fish hooks and weights for fish nets were recovered from Grossgartach and Rössen pits and graves. A notable amount of freshwater mussel shell was discovered in Grossgartach pits, especially in Kolbsheim. These may have been a food source, yet they could also have been raw material for beads. The mean proportion of fish among Grossgartach individuals (4.0 ± 4.0%), and those from other sites (4.0 ± 4.0%) is identical.

**Fig. 20:**
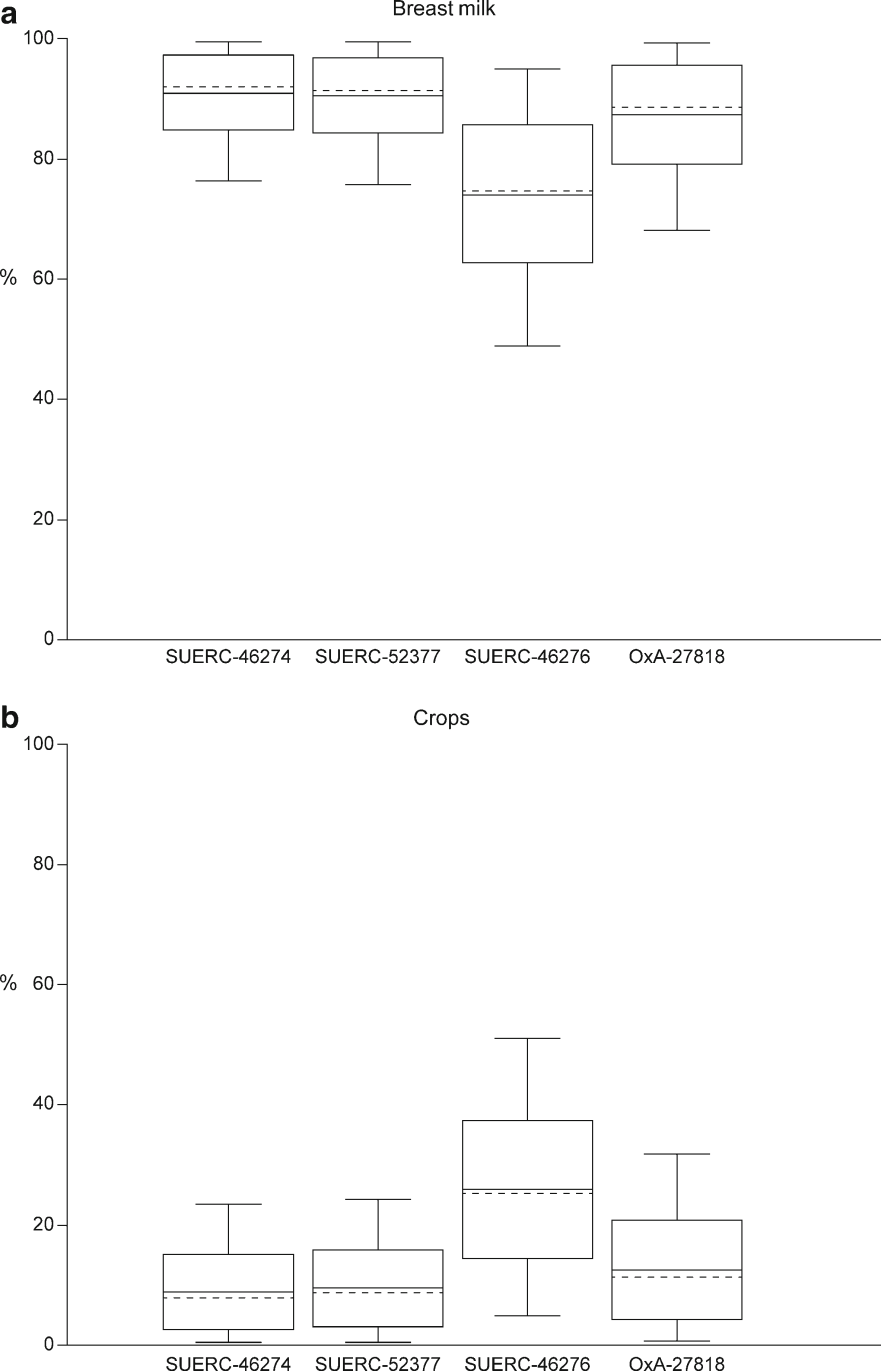
Box plots of the FRUITS estimations for the two diet sources: **a** breast milk and **b** crops for children 4 years old and under that have measured stable isotopic values (Tables 2 and 5). The *boxes* provide a 68% confidence interval while the *whiskers* provide a 95% confidence interval. The *horizontal continuous line* indicates the average while the *horizontal discontinuous line* indicates the median (50th percentile)

The dietary estimates of the mean percentage (and standard deviation) for each of the possible food sources for the infant skeletons are shown in Table 5 (and see Fig. 20). The breast milk isotopic signature proxy, derived from child-bearing age female isotope means, plus a diet-to-consumer offset for the infants of 1.0 ± 0.5‰ for δ^13^C and 3.0 ± 0.5‰ for δ^15^N (Fuller et al. 2006; Katzenberg et al. 1996; White and Schwartz 1994), produces reasonable results for proportions of breast milk feeding plus cereals as weaning food (Table 5). The case of the isotopic signatures of a 10–12-month-old infant (Ros.89; SUERC-46276), which plot among the adults and sub-adults (Fig. 18), is driven by less enriched δ^15^N compared with other infants. The depleted ^15^N in this infant may suggest a failure to thrive or obtain sufficient nourishment, as this isotopic signature does not show the δ^15^N enrichment over nursing mothers expected for infants on breast milk.

**Table 5:** FRUITS (Fernandes *et al*. 2014) estimates of diet proportions of the two food sources for children of 4 years and under dated as part of this study (weighted averages of replicate δ^13^C and δ^15^N analyses measured by isotope ratio mass spectrometry; see Tables 1 and 2)

Laboratory number	Burial	Estimated age	δ^13^C (‰)	δ^15^N (‰)	FRUITS diet proportion estimates	
					Crops	Breast milk
SUERC-46274	Ros.85	1–4 years	−19.7 ± 0.2	12.7 ± 0.3	8.9 ± 6.3%	91.1 ± 6.3%
SUERC-52377	Ober.4106	0–4 years	−20.1 ± 0.2	13.4 ± 0.3	9.5 ± 6.3%	90.5 ± 6.3%
SUERC-46276	Ros.89	10–12 months	−20.1 ± 0.2	9.8 ± 0.3	25.9 ± 11.7%	74.1 ± 11.7%
OxA-27818	Ros.87	3–5 months	−19.4 ± 0.2	10.6 ± 0.3	12.6 ± 8.3%	87.4 ± 8.3%

This exercise in estimating breast milk proportions in infant diets does not take account of the possibility that infant diets were supplemented with non-human milk (Howcroft et al. 2012). The use of non-human milk to supplement or replace breast feeding, and the introduction of weaning foods such as cooked cereals, is thought to have negative effects on infant survival, mainly due to earlier weaning (Howcroft et al. 2012, p.16; Molleson 1994).

### Mixed-Source Modelling

The sensitivity analyses for the LBK and Middle Neolithic sequences are identical in form to the models defined in Figs. 8, 15 and 16, except that we have constructed an individual calibration curve for each dated human (and the dog) that accounts for the proportion of fish in their diet.

Unfortunately, no measurements of the freshwater reservoir in the waters of the upper Rhine are currently available, and so we have used a generic offset of 500 ± 100 BP. On the basis of the evidence that is currently available, this is probably a reasonable average of the marine offset in the North Atlantic (from which any migratory species are likely to derive) and the local freshwater offset in the upper Rhine (cf. Keaveney and Reimer 2012; Bonsall et al. 2015). We use this reservoir, offset from the atmospheric calibration dataset (Reimer et al. 2013), and the Mix_Curves function of OxCal v4.2 (Bronk Ramsey 2001, amended following Jones and Nicholls 2001).

The individual mixed-source calibration curve for each dated human incorporates the aquatic reservoir in the proportion suggested by the dietary estimates provided by the FRUITS model in that particular person (Table 4). So, for example OxA-27813 (Ros.112) has been calibrated using a calibration curve including a component of 4.2 ± 3.8% aquatic resources (note that the proportion of any curve is constrained to be 0–100%). The remainder of diet sources will be in equilibrium with the contemporary atmosphere and have been calibrated using IntCal13 (Reimer et al. 2013).

There are 18 human skeletons dated at the Poznań laboratory from Middle Neolithic burials for which there are no stable isotope values. We calculated the average proportion of fish in the diets of Middle Neolithic adult females (4.1 ± 3.7%), unsexed adults (4.2 ± 3.8%), and sub-adults (3.7 ± 3.4%) and used these as an estimate of the likely fish component of the diet in these individuals. We used the average proportion of breast milk (86.0 ± 8.0%) for children aged 4 years or younger, multiplied by the average proportion of fish in the diets of Middle Neolithic adult females (4.1 ± 3.7%) for infants of this age without measured stable isotopic values. The single dog skeleton (Poz-45612) has been calibrated using the average proportion of fish in the diets of the Michelsberg-period dogs from Heilbronn-Klingenberg ‘Schlossberg’, Baden-Württemberg (11.5 ± 10.0%; Seidel et al. 2016).

Only a single human burial (OxA-30789; Osth. 227) was dated from the Early Neolithic sequence, and this is excluded from the preferred LBK model (Fig. 8) as it is anomalously early. This result was incorporated into the sensitivity analysis including the estimated proportion of fish in this individual’s diet (5.0 ± 4.0%; Table 4), but this date still has poor individual agreement (A: 7) and brings the model into poor overall agreement (Amodel: 40). It is unlikely, therefore, that this anomaly can be explained by a reservoir effect. This single result has negligible impact on the overall model (the medians of the key parameters between the preferred model and the LBK sensitivity analysis vary by a maximum of 2 years).

Far more human burials have been dated from the Middle Neolithic sequence, and the potential dietary offsets consequence have a greater impact on the outputs of the variant model. The highest posterior density estimates for the key parameters from the preferred Middle Neolithic model (Figs. 15 and 16) and the sensitivity analysis are given in Table 6. The model outputs from the sensitivity analysis are slightly later than those from the fully terrestrial model. This is particularly true of the estimated dates of the Planig-Friedberg phase, which begins and ends several decades later according to the sensitivity analysis (the medians of the distributions for the start and end of this phase vary by 38 and 36 years, respectively). The medians of all other phase boundaries between the two models vary by less than 20 years.

**Table 6:** Highest posterior density intervals for key parameters from the preferred model (Figs. 15 and 16) and the sensitivity analysis incorporating the dietary offsets suggested by the FRUITS modelling (see text) for the Middle Neolithic sequence

Parameter	Preferred models (Figs. 8, 15 and 16)		Sensitivity analyses (see text)	
	Highest posterior density interval		Highest posterior density interval	
	95% probability	68% probability	95% probability	68% probability
*s* *tart start HK*	*4910–4725 cal BC*	*4835–4745 cal BC*	*4885–4715 cal BC*	*4810–4730 cal BC*
*end start HK*	*4850–4715 cal BC*	*4795–4730 cal BC*	*4830–4700 cal BC*	*4775–4715 cal BC*
*e* *nd HK/start GG*	*4765–4705 cal BC*	*4745–4720 cal BC*	*4750–4690 cal BC*	*4735–4700 cal BC*
*e* *nd GG/start P-F*	*4690–4610 cal BC*	*4680–4640 cal BC*	*4680–4575 cal BC*	*4660–4640 cal BC (16%)* or *4635–4590 cal BC (52%)*
*e* *nd P-F/start RS*	*4670–4565 cal BC*	*4645–4585 cal BC*	*4645–4625 cal BC (2%)* or *4620–4530 cal BC (93%)*	*4600–4555 cal BC*
*e* *nd RS/start BI*	*4515–4395 cal BC*	*4495–4430 cal BC*	*4505–4385 cal BC*	*4480–4420 cal BC*
*e* *nd BI (Lower Alsace)/start B-O*	*4435–4345 cal BC*	*4400–4355 cal BC*	*4415–4340 cal BC*	*4390–4350 cal BC*
*e* *nd B-O/start BORS I*	*4295–4165 cal BC*	*4275–4210 cal BC*	*4290–4160 cal BC*	*4270–4210 cal BC*
*e* *nd BORS I/start BORS II*	*4140–4025 cal BC*	*4130–4065 cal BC*	*4140–4025 cal BC*	*4130–4065 cal BC*
*s* *tart end BORS II*	*4130–3880 cal BC*	*4075–3965 cal BC*	*4130–3885 cal BC*	*4075–3965 cal BC*
*e* *nd end BORS II*	*4115–3805 cal BC*	*4045–3920 cal BC*	*4115–3805 cal BC*	*4045–3920 cal BC*

Given the uncertainties involved in estimating past human diet accurately from stable isotopic values and the complete absence of data for any freshwater reservoir effect in the waters of the upper Rhine, we prefer the fully terrestrial models presented in Figs. 8, 15 and 16. The alternative models have, however, demonstrated that radiocarbon reservoir effects are unlikely to affect the chronologies presented here by more than a few decades at most.

## Discussion

### The Cultural Project: the Rhythm and Tempo of Change

In contrast to the passivity of traditional chronological charts or the smoothed transitions produced by summed probability distributions, the more precise date estimates offered by formal modelling in combination with rigorous seriation and typology have revealed a Neolithic world in Lower Alsace busy with comings and goings, tinkerings and adjustments, and relocations and realignments. More precise date estimates show that being busy was not always conducted at the same tempo, though most of the changes we have described would have been perceptible within a lifetime scale, and many of them within one to two generations. If a general characterisation of human life was of a ‘continual transaction, a continual argument’, with culture as a ‘reservoir of resources that people use to work on each other’ (Carrithers 2010, pp. 160 and 162), this seems to fit well with the long Danubian tradition of Lower Alsace and its surrounds. Given that, we argue that the approach of formal modelling helps to uncover much more detailed histories of social and cultural interaction than would otherwise be available, within which human agency becomes much more visible.

The formal modelling approach in combination with material analysis has also highlighted quite radical shifts, including in the latter part of the Middle Neolithic sequence, and the major discontinuity of the gap between late LBK and Hinkelstein. Agency involves choice, but whether choice was an option in the latter situation depends on how the causes of such a hiatus are to be explained. We go on now to reflect in more detail on the components of the sequence and to explore wider aspects of the proposed gap.

### Assessment: Strengths and Weaknesses

The new series of modelled date estimates produced by this project has broadly confirmed the previously proposed sequence, but has significantly modified our understanding of the rhythm of change, notably so for the start of the Middle Neolithic. Thus the Hinkelstein should no longer be considered as contemporary to the end of the LBK in Lower Alsace, but appeared significantly later; the Grossgartach phase is now placed after 4750 cal BC, with the Rössen phase beginning about 4650 cal BC at the earliest. The Planig-Friedberg is now dated in its own right, whereas previously it had been included with the Grossgartach. If the date range of the Bischheim has only altered a little, the duration of Bruebach-Oberbergen has extended; it seems that this group continued beyond the previously estimated end date of the forty-third century. The BORS now ends a little earlier, in the middle of the forty-first century.

**Fig. 21:**
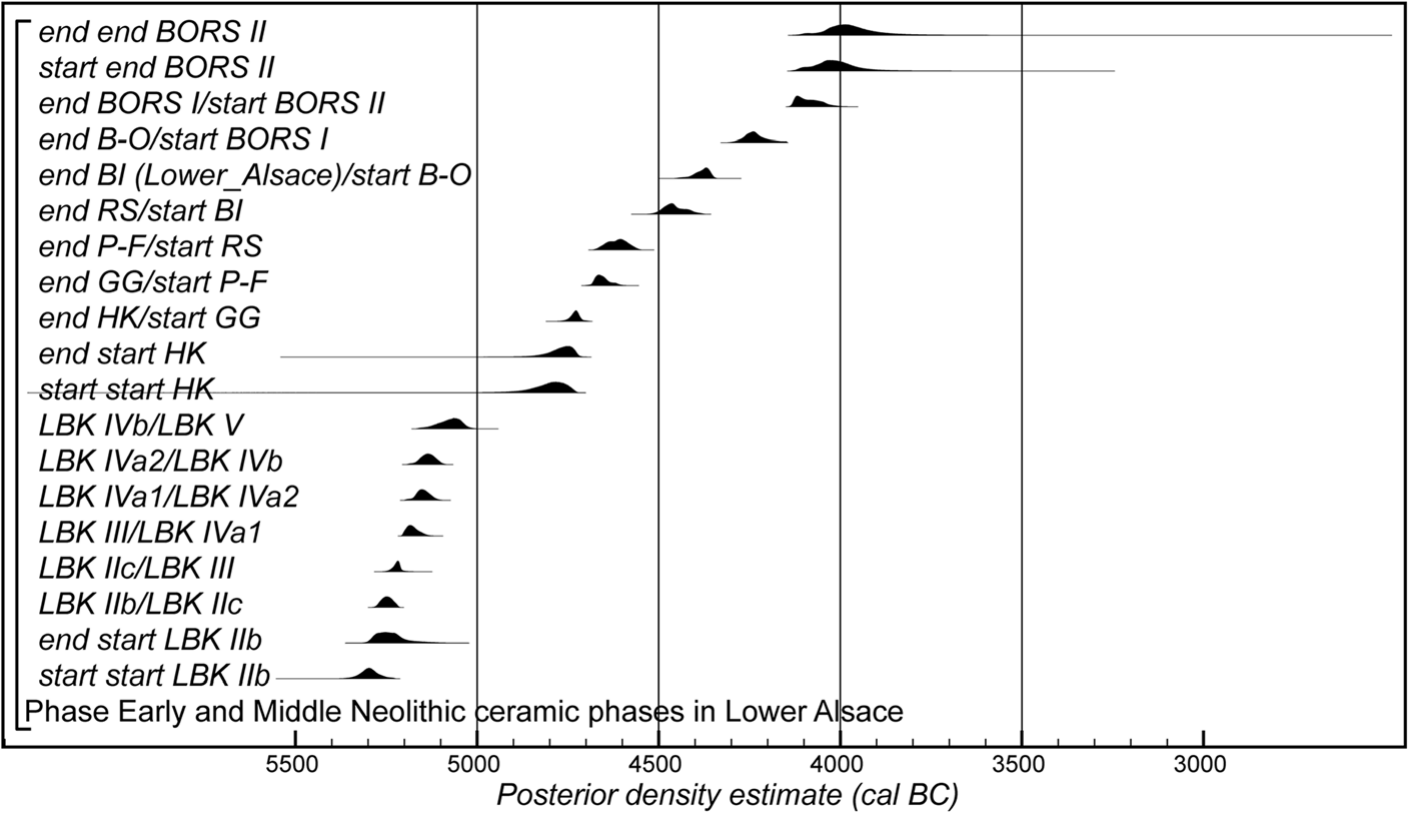
Key parameters for the chronology of LBK and Middle Neolithic ceramics in Lower Alsace (derived from the models defined in Figs. 8, 15 and 16)

**Fig. 22:**
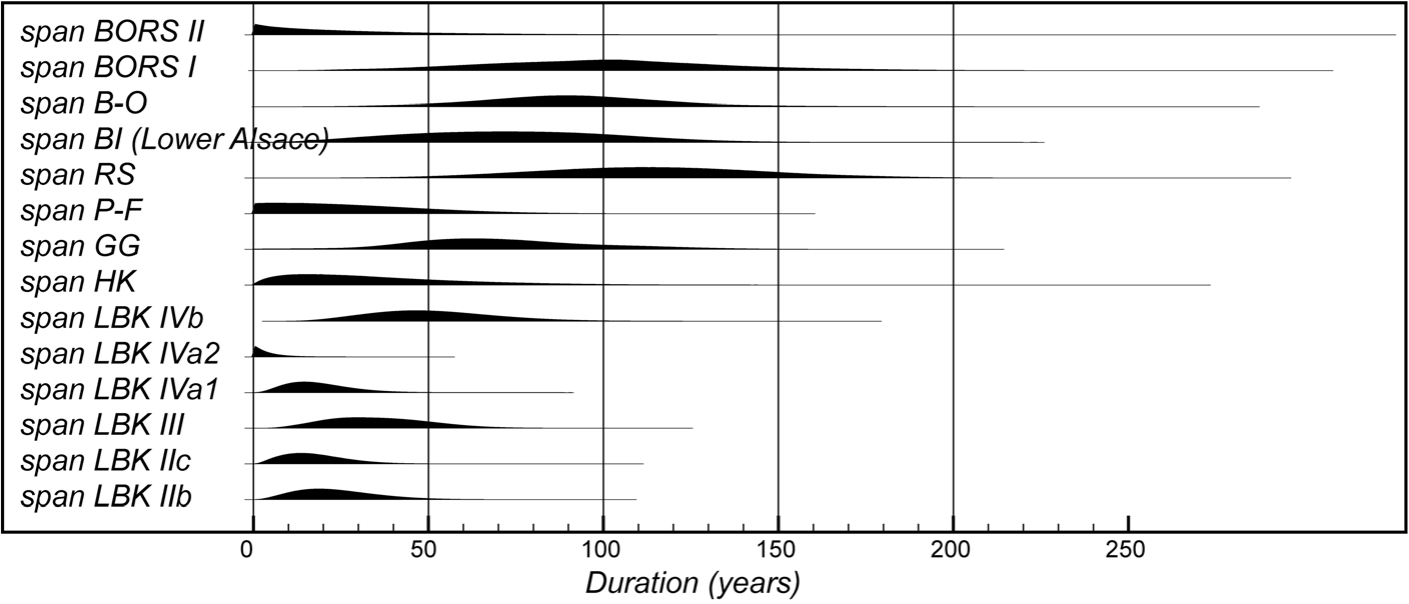
Posterior density estimates for the durations of the LBK and Middle Neolithic ceramic phases in Lower Alsace (derived from the models defined in Figs. 8, 15 and 16)

Before we discuss this in more detail, we need to reflect on the strengths and weaknesses of the models presented. A summary of the date estimates for the phase boundaries in the seriations described above is provided in Fig. 21. A summary of the duration of each phase is provided in Fig. 22.

The radiocarbon dates are in good agreement with the sequences suggested by the seriations (Figs. 5, 10 and 13) in both the LBK and Middle Neolithic periods (Amodel: 61; Fig. 8; and Amodel: 100; Figs. 15 and 16 respectively). Only three measurements (out of 56) appear to be misfits in the LBK model (Ly-865, SUERC-55325 and OxA-30789), and only two of the dated samples seem to be residual (an articulating group of cattle bones from pit 1807 at Bischoffsheim ‘AFUA du Stade’ and a pair of sheep/goat mandibles from pit 3034 at Rosheim ‘Rittergass’). No misfits were identified in the Middle Neolithic model (from the 96 measurements included). None of the submitted samples appear to be residual, although a number of measurements from disarticulated or unidentified bone that we have inherited from previous researchers have been modelled as *termini post quos* for their contexts. This is a conservative approach, since it is probable that some of this short-life material is of the same age as the feature from which it was recovered. These results are, however, a small proportion of the total (12%) and so we have chosen this approach to provide the most robust model possible.

The compatibility of our radiocarbon dates with the archaeological sequence vindicates the strict criteria for sample selection and replication strategy that we have implemented (see above). We suggest that further sampling to extend and improve the chronologies present here *must* adopt similar criteria. Short-life, single-entity samples are simply not good enough—they *must* be demonstrably contemporary with demonstrably closed assemblages of material culture.

The greatest weakness in the chronologies presented here are clearly the complete lack of dated samples associated with the final phase of the LBK seriation (LBK V) and with the first phase of the BORS seriation (BORS 0). The number of dated samples in the Hinkelstein and BORS II phases of the Middle Neolithic seriation are also clearly inadequate. It may be no coincidence that it is at these times that major transitions occur in the archaeology of Alsace. The resolution of these weaknesses must come piecemeal as suitable, clearly closed assemblages of pottery are recovered in association with clearly contemporary datable material. Dating lower-quality samples in a rush to refine these chronologies will simply undermine their reliability.

### LBK Narrative and Commentary

On the basis of our model (Fig. 8), the appearance of the LBK in Lower Alsace in LBK IIb was rapid. In the space of two generations, nearly all the loess areas were densely occupied. Only the north part of the Kochersberg plateau, north of Strasbourg, and the Andlau terrace, in the south of Lower Alsace, were unoccupied. With 36 sites known, LBK IIb marks a surge in the settlement of the region, which is probably best explained by the influx of a new population. The origin of these people was probably in the Neckar region or in the *Rubané du Nord Ouest* region, which in its early phase shares numerous traits with Lower Alsace (Lefranc 2007). It is very probable that at this time, stage IIb was also established in the area of Colmar, forming a cultural frontier that separates the Lower and Upper Alsace groups, which differ as much in their ceramic styles as in their funerary rites, livestock management strategies and architecture (Jeunesse 1995). One proposed hypothesis identifies two migration routes into Alsace by Danubian groups: one stream following the Neckar valley, the other, the Danube valley (Lefranc 2006). The meeting point of these two currents established a cultural boundary which endured throughout the Neolithic.

The transitional LBK IIc phase is also short-lived, and things run on into phase III. In LBK III, the number of known sites falls to 17. This apparent decrease translates to the abandonment of numerous sites and the absence of newly founded settlements. This phenomenon, observed in other regions, is sometimes associated with a new period of mobility and expansion to the west (Strien and Gronenborn 2005). In Upper Alsace, by contrast, the fact that groups are thought to have left for the Marne valley to the west does not translate to a decrease in the density of sites; on the contrary, the number of LBK III sites in Upper Alsace doubles compared with LBK II, and it could be this strong demographic expansion and its consequence—the filling up of the most fertile soils—that motivated the move to the west.

The formally modelled date estimates also suggest a relatively rapid series of shifts in the decorative motifs on pottery, and it is tempting to see this as part of the social conditions in which settlement was established by a new population in a new region. It was no doubt important for new identities to be established and reinforced, with decorated pottery, putatively involved in processes of social interaction beyond the households where it was created, for example through the sharing of food, an important means of signalling both affiliation and difference.

In LBK IV, more than 70 sites are known in Lower Alsace. In the first half of this stage, settlement stays relatively stable. The pattern of settlements continues from phase III and the ceramic decoration is characterised by a very regular stylistic development without significant rupture, leading, in the space of about two generations, to phase IVb. The relatively long phase IVb (two or three generations) is then marked by an increased number of settlements, which double compared with phases IVa1 and IVa2 (which together persist for a comparable two to three generations); these include now seven new settlements to the north of Kochersberg, in a new area previously uncolonised.

In the context of this further apparent intensification of settlement and hypothetically increased social tensions, there are significant changes in pottery. The decorative repertoire, which had previously seen an uninterrupted continuum, markedly expands; the ‘traditional’ angled decoration now competes with the orthogonal ‘T’ style and horizontal ladder motifs. The choice of style seems to be made at the scale of the household. It is possible to distinguish in varying quantities assemblages dominated by the ‘traditional’ decoration, assemblages dominated by the ‘orthogonal’ decoration, and ‘mixed’ assemblages, in which the two compositions appear in equal proportions (Jeunesse 2008; Lefranc 2013). Again pottery seems central to social interaction in conditions of relatively rapid change.

We can also emphasise that phase IV in Lower Alsace is marked by the appearance of large cemeteries, such as Vendenheim (Boës et al. 2007), Souffelweyersheim (Jeunesse 1997) or the recently discovered Ingenheim (Lefranc et al. 2014). In contrast to what is known in the neighbouring regions of the Rhine and Upper Alsace, no cemetery is yet attributable to LBK II and III in Lower Alsace (Lefranc et al. 2014; Denaire and Lefranc 2014). The dead now became a socially significant resource and point of reference. Distinct funerary rituals at such sites could be the expression of different local identities (Boës et al. 2007; Jeunesse 2009).

In the context of settlement intensification, it is worth noting the suggestion that, on the basis of similarities in the pottery, the LBK in the Aisne valley could be derived from Lower Alsace, by a process of outward migration, via the Marne (Jeunesse 2008).

After the dynamic shifts which characterise phase IVb in Lower Alsace, there was a dramatic shrinkage of the LBK occupation of Lower Alsace in phase V. The Kochersberg plateau, densely populated in phase IVb, becomes deserted and LBK occupation is concentrated in a small area along the foothills of the Vosges uplands.

### The Transition from the LBK to the Middle Neolithic

Possibly the most surprising outcome of our models is the yawning gap between the end of the LBK IVb in Lower Alsace in *5145–5020 cal BC* (*95% probability*; *LBK IVb/LBK V*; Fig. 24), probably in *5105–5040 cal BC* (*68% probability*), and the initial appearance of Hinkelstein pottery in *4910–4725 cal BC* (*95% probability*; *start start HK*; Fig. 24), probably in *4835–4745 cal BC* (*68% probability*). This is a gap of *145–385 years* (*95% probability*; *gap Lower Alsace*; Fig. 25), probably of *225–335 years* (*68% probability*). Given the limited quantity of LBK V material in the region, we think it is implausible that the final phase of the LBK occupies this entire period. However, LBK V in Lower Alsace could have lasted a generation or two, a supposition we have based on the duration of other LBK phases and the sparseness of the material finds. Probably there was in reality a hiatus in Lower Alsace between the final LBK occupation towards the end of the fifty-first century cal BC and the appearance of the first Hinkelstein pottery in the first half of the forty-eighth century cal BC.

As both the LBK and Hinkelstein are found in a wider geographical area of the Rhineland, we decided to model the chronologies of the final LBK phase (Meier-Arendt V, Strien 9 and Dorn-Ihmig IId) and the Hinkelstein style in the upper and middle Rhine catchment. We compiled a catalogue of radiocarbon dates on short-life materials from features that contained assemblages of the relevant pottery. The haul was pitiful.

**Fig. 23:**
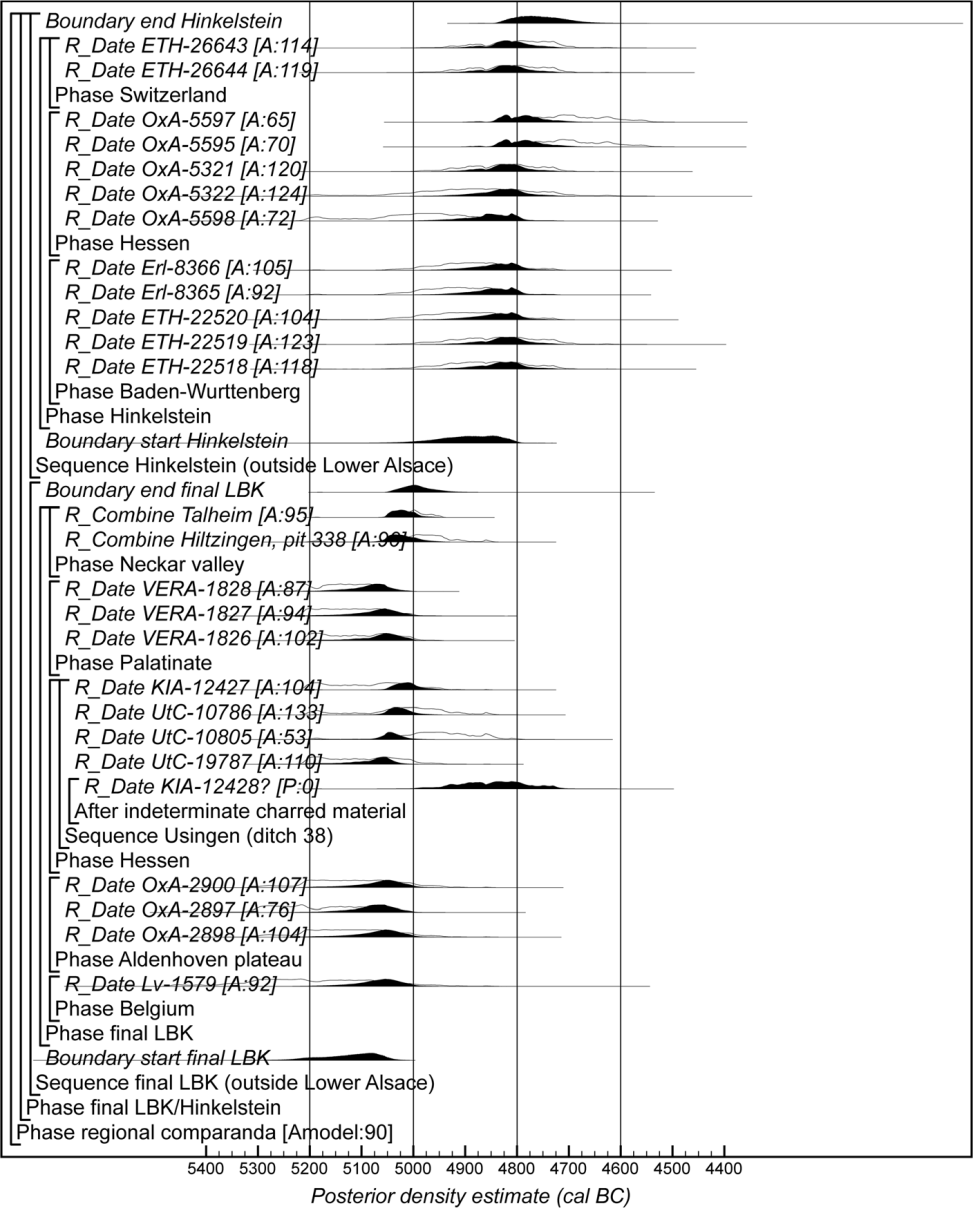
Probability distributions of radiocarbon dates from the final phase of LBK pottery, and for Hinkelstein pottery, elsewhere in the Upper Rhineland, Hessen and the Neckar valley (excluding Lower Alsace). The format is identical to that of Fig. 8. The *large square brackets down the left-hand side*, *along with the OxCal keywords*, define the overall model exactly

Radiocarbon results on short-life materials related to Hinkelstein pottery outside Lower Alsace are listed in Table 7 (we have not included any of the radiocarbon dates on human bone from the Trebur cemetery, undertaken at the Heidelberg laboratory in the 1980s, since a number of these appear to be anomalously recent for technical reasons; Spatz 2001). A model for the currency of Hinkelstein pottery outside Lower Alsace is shown in Fig. 23. The results form a coherent group with no outliers. This suggests that all the dated samples may have robust association with Hinkelstein pottery; that is, we have included disarticulated animal bones in this model and they appear not to be residual. This model suggests that Hinkelstein pottery appeared in *5015–4795 cal BC* (*95% probability*; *start Hinkelstein*; Fig. 23), probably in *4940–4820 cal BC* (*68% probability*). The imprecision of this estimate reflects the small number of radiocarbon dates available, although it is skewed towards a later beginning, probably in the forty-ninth century cal BC. It is clear (*89% probable*) that the Hinkelstein style began elsewhere in the Rhineland before it occurs in Lower Alsace. The demise of the Hinkelstein style in the Rhineland occurred in *4840–4650 cal BC* (*95% probability*; *end Hinkelstein*; Fig. 23), probably in *4815–4715 cal BC* (*68% probability*). This date estimate is compatible with the end of this style and the transition to Grossgartach in Lower Alsace.

**Table 7:** Radiocarbon and stable isotopic measurements for Hinkelstein pottery in the Upper and Middle Rhine (highest posterior density intervals are provided for samples of intrinsic interest or features and are derived from the model defined in Fig. 23)

Laboratory number	Material and stratigraphic details	Radiocarbon age (BP)	δ^13^C (‰)	Highest posterior density interval (95% probability)	References
Baden-Wurttemberg					
ETH-22518	Animal bone from Heilbronn-Neckargartach pit 2542/20	5970 ± 65	−21.9 ± 1.2	*4935–4735 cal BC*	Eisenhauer (2002)
ETH-22519	Animal bone from Heilbronn-Neckargartach pit 2543/2	5950 ± 70	−22.5 ± 1.2	*4925–4725 cal BC*	Eisenhauer (2002)
ETH-22520	Animal bone from Heilbronn-Neckargartach from pit 2745/6 (which also contains Grossgartach pottery)	6005 ± 65	−20.4 ± 1.2	*4945–4745 cal BC*	Eisenhauer (2002)
Erl-8365	Antler from a pit at Edingen-Neckarhausen	6018 ± 56	−23.0		Stadler and Wirth (2006)
Erl-8366	Animal bone from the same pit as Erl-8365 at Edingen-Neckarhausen	5995 ± 58	−24.4		Stadler and Wirth (2006)
Hessen					
OxA-5598	Femur from articulated human skeleton in grave 127, Trebur	6065 ± 70	−19.7	*4970–4770 cal BC*	Spatz (2001)
OxA-5322	Bone from articulated human skeleton in grave 132, Trebur	5980 ± 90	−19.4	*4940–4725 cal BC*	Spatz (2001)
OxA-5321	Bone from articulated human skeleton in grave 68, Trebur	5945 ± 55	−19.1	*4915–4725 cal BC*	Spatz (2001)
OxA-5595	Femur from articulated human skeleton in grave 52, Trebur	5840 ± 55	−19.9	*4885–4865 cal BC (1%)* or *4855–4690 cal BC (94%)*	Spatz (2001)
OxA-5597	Femur from articulated human skeleton in grave 107, Trebur	5835 ± 55	−19.1	*4880–4865 cal BC (1%)* or *4850–4690 cal BC (94%)*	Spatz (2001)
Switzerland					
ETH-26644	Hazelnut shell from mixed layer associated with Hinkelstein sherds, western activity zone, Zizers	5935 ± 55			Seifert (2012)
ETH-26643	Hazelnut shell from mixed layer associated with Hinkelstein sherds, western activity zone, Zizers	5920 ± 55			Seifert (2012)

**Fig. 24:**
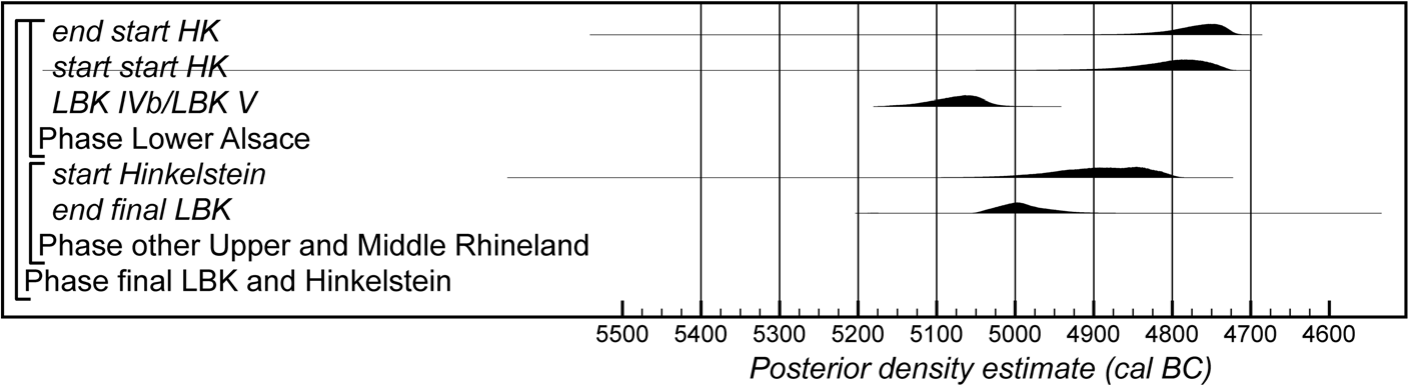
Posterior density estimates for the end of use the use of LBK ceramics and the start of the use of Hinkelstein ceramics in Lower Alsace (above) and elsewhere in the Upper Rhineland, Hessen and the Neckar valley (below), derived from the models defined in Figs. 8, 15, 16 and 23

Radiocarbon results on short-life materials related to final LBK ceramics outside Lower Alsace are listed in Table 8. Three of the 24 results considered appear to be anomalous. Vera-2021 is a clear outlier from the group of measurements from the Talheim burial pit (Wild et al. 2004, p. 381). Vera-1829, a human bone from ditch 282 from Herxheim, clearly contained exogenous older carbon that was not removed during pretreatment. Finally, KIA-14248, a very small sample of indeterminate charred material from the basal fill from ditch 38 from Usingen, Hesse, appears to be anomalously recent in relation to larger samples from the overlying fills of the ditch (Wotzka et al. 2001, footnote 36). A model for the currency of final LBK ceramics outside Lower Alsace is shown in Fig. 23. This model includes the stratigraphic sequence of samples through the ditch fills at Usingen, and takes weighted means before calibration and modelling of the measurements from Talheim (since archaeologically we interpret this as a single event) and pit 338 at Hilzingen (since the two dated samples were bulk aliquots of the carbonised plant remains from this feature). This model suggests that the final phase of LBK ceramics outside Lower Alsace began in *5240–5030 cal BC* (*95% probability*; *start final LBK*; Fig. 23), probably in *5165–5050 cal BC* (*68% probability*). The inadequate number of measurements means that this distribution is imprecise, but again it is skewed towards a later date in the fifty-first century cal BC. This is compatible with a tree-ring date of 5057 ± 5 BC for well lining 2 at Erkelenz-Kückhoven, which contains a similar assemblage, and with the boundary between LBK IVb and LBK V in Lower Alsace (Fig. 24). The last LBK pottery occurred in the Rhineland in *5050–4920 cal BC* (*95% probability*; *end final LBK*; Fig. 23), probably in *5030–4965 cal BC* (*68% probability*). It seems to have occurred in the last decades of the fifty-first century cal BC in a number of areas, including in the Neckar valley where the mass burial pit at Talheim was dug in *5055–4970 cal BC* (*95% probability*; *Talheim*; Fig. 23), probably in *5045–5000 cal BC* (*68% probability*).^1^

**Table 8:** Radiocarbon and stable isotopic measurements for the final phase of LBK pottery in the Upper and Middle Rhine (highest posterior density intervals are provided for samples of intrinsic interest or features and are derived from the model defined in Fig. 23; reported stable isotopic values were measured by isotopic ratio mass spectrometry)

Laboratory number	Material and stratigraphic details	Radiocarbon age (BP)	δ^13^C (‰)	Highest posterior density interval (95% probability)	References
Belgium					
Lv-1579	Charred cereal grain from pit 51 at Darion, Liège	6240 ± 100		*5190–4985 cal BC*	Cahen (1984)
Aldenhoven plateau					
OxA-2898	Carbonised cereal from pit 800-3 at Langweiler 2	6170 ± 60	−23.7	*5185–4990 cal BC*	Farruggia *et al*. (1973)
OxA-2897	Carbonised cereal from pit 785-14 at Langweiler 2	6230 ± 60	−24.4	*5180–4995 cal BC*	
OxA-2900	Carbonised cereals from pit 3813-7 at Langweiler 8	6160 ± 60	−23.4	*5190–4985 cal BC*	Boelicke *et al*. (1988)
Hessen					
UtC-19787	Carbonised legumes from layer XXVI in ditch 38 at Usingen	6142 ± 40			Wotzka *et al*. (2001)
UtC-10786	Carbonised cereal from layer XVI in ditch 38 at Usingen	6091 ± 48			
UtC-10805	Charred grain from layer XXXI in ditch 38 at Usingen	6050 ± 50			
KIA-12427	Pea (cf. *Pisum sativum*) from layer XVIII in ditch 38 at Usingen	6132 ± 46			
KIA-12428	Indeterminate charred material from layer III of ditch 38 at Usingen	5960 ± 52		–	
Palatinate					
VERA-1826	Human bone from ditch 281-14 at Herxheim	6145 ± 35	−19.1 ± 1.3	*5190–5165 (2%)* or *5160–4995 (93%)*	Wild *et al*. (2004) and Häusser (2000)
VERA-1827	Human bone from ditch 281-117 at Herxheim	6165 ± 40	−20.1 ± 1.2	*5175–4995 cal BC*	
VERA-1828	Human bone from ditch 282-7 at Herxheim	6190 ± 30	−20.7 ± 1.3	*5175–5015 cal BC*	
VERA-1829	Human bone from ditch 282-86 at Herxheim	6995 ± 35		–	Häusser (2000)
Neckar valley					
Hd-9913/9765	Carbonised cereals from pit 338 at Hilzingen	6000 ± 50		*5075–4945 cal BC*	Fritsch (1998)
Hd-9914/9766	Carbonised peas from the same pit as Hd-9914/9766	6130 ± 45			
Hd-8606/8827	SK 4, human bone from Talheim	5960 ± 80	−21.2	*5055–4970 cal BC*	Wild *et al*. (2004), Wahl and König (1987) and Biel (1987)
Hd-8607/8828	SK 14, human bone from Talheim	6045 ± 60	−20.6		
VERA-2022	83/1, human bone from Talheim	6130 ± 35	−21.9 ± 0.5		
VERA-2023	83/10, human bone from Talheim	6085 ± 30	−22.0 ± 0.4		
VERA-2025	SK22, human bone from Talheim	6015 ± 35	−21.5 ± 0.5		
VERA-2026	83/13, human bone from Talheim	6095 ± 35	−21.4 ± 0.3		
VERA-2046	SK21?, human bone from Talheim	6115 ± 35	−22.7 ± 1.1		
VERA-2047	83/19, human bone from Talheim	6140 ± 40	−21.6 ± 0.9		
VERA-2021	SK16, human bone from Talheim	5930 ± 35	−22.9 ± 0.5	–	Wild *et al*. (2004), Wahl and König (1987) and Biel (1987)

**Fig. 25:**
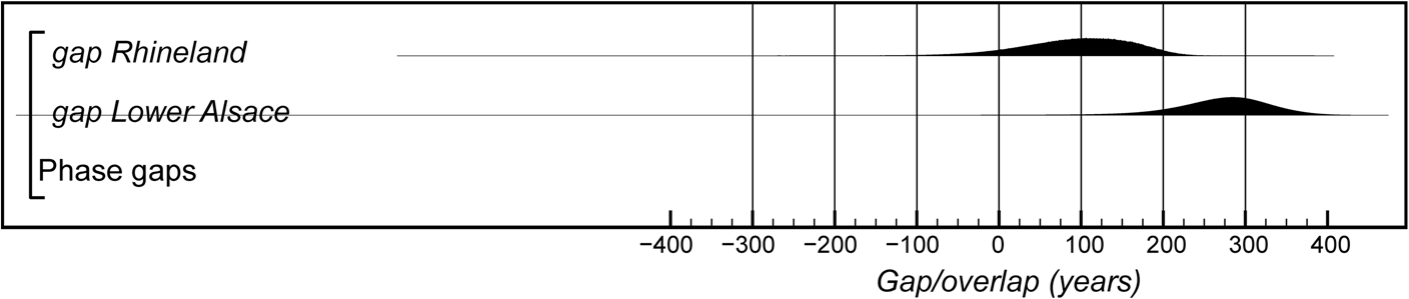
Posterior density estimates for the gap between the last LBK ceramics and the first Hinkelstein ceramics in Lower Alsace and elsewhere in the Upper Rhineland, Hessen and the Neckar valley (derived from the models defined in Figs 8, 15, 16 and 23)

In the regions of the Rhineland in which we currently have radiocarbon dates, there appears to be a hiatus between the end of the LBK and the appearance of Hinkelstein ceramics (*91% probable*). This gap lasted for *−45 ± 220 years (95% probability*; *gap Rhineland*; Fig. 25), probably for *40–175 years* (*68% probability*).^2^

The number of radiocarbon dates on short-life material associated with final LBK and Hinkelstein ceramics in the Rhine catchment is clearly inadequate. The available dates, however, do cover the geographical spread of these pottery types. The observed gap in this region has implications for the continuity of populations in the early centuries of the fifth millennium cal BC.

The reality of a gap is also suggested by the lack of Hinkelstein sites in Upper Alsace. A similar situation exists in the region of the Aldenhovener Platte, where the sequence appears to resume with Grossgartach sites. In this case, regional pollen diagrams, with regeneration followed by indications of renewed clearance (Kalis and Zimmermann 1988), appear to provide supporting evidence for a break or decline in settlement.

In what is normally taken as the core Hinkelstein area around Worms in the middle Rhine valley, traditional arguments suggest contact between the users of late LBK and Hinkelstein pottery (Jeunesse 1999; Spatz 2001). It is a pity that it has not been possible to date the putatively earliest Hinkelstein graves from Worms. It is stylistic analysis of the pottery in the Worms region which has suggested late LBK–Hinkelstein contact. Several pots in Hinkelstein graves in the Worms cemeteries have decoration closely linked to that of the LBK (Meier-Arendt 1975, pp. 128–31), and one can cite the decorated pot from Mainz-Weisenau, which has been attributed to the LBK, but has Hinkelstein-decoration-like fabric (Meier-Arendt 1975, p. 131, Abb. 24). Several assemblages with styles of pottery in them have often been used as the basis of LBK–Hinkelstein contact. Whether these are really closed assemblages, is open to question in many cases. Only that from Köln-Lindenthal (Buttler 1935) survives rigorous analysis; here a pit with sherds of the final stage of the LBK contained also an indisputably Hinkelstein pot (Spatz 2001). Note that Köln-Lindenthal lies outside the Hinkelstein distribution. So at face value, the Hinkelstein pot can only be an import contemporary with the sherds with which it was deposited, though with older discoveries one is left wondering whether recuts have been missed. It is also worth noting the presence in several Hinkelstein graves in the Worms area and Trebur graveyard of *Spondylus* ornaments seemingly identical to those of the LBK (Meier-Arendt 1975; Spatz 1999). Other ornaments, especially necklaces with alternating cylindrical and elongated beads of deer teeth and shell, also strongly evoke LBK forms (Bonnardin 2009).

### Middle Neolithic Narrative and Commentary

According to our results for Lower Alsace, the introduction of the Hinkelstein style is separate from the end of the LBK, and at a later date than accepted in research on the region up till now. This style now appears to come in rapidly, in less than 20 years. A short duration of less than two generations may be part of the explanation for only five sites being known. These are found around the edges of the areas formerly occupied by the LBK, quite close to the Rhine, which was presumably the communication route with the middle Rhine, from where the Hinkelstein style derives. The general impression is of pioneer settlers; no houses have yet been found. At Erstein and Entzheim, Hinkelstein graves start a mortuary tradition which was to be maintained till the end of the Grossgartach and the Planig-Friedberg phases (Denaire 2009, pp. 378 and 381–2; Leprovost and Queyras 2011). These two sites also show the links between the Hinkelstein and Grossgartach styles. The transition between them seems gradual, mostly within the same areas, but with a little expansion to southern Lower Alsace.

It is only during the Grossgartach phase that all the territory occupied during the LBK was re-inhabited (Denaire 2009, pp. 255–66). With a duration of perhaps only three generations, the density of Grossgartach sites in Lower Alsace is striking, matching that of the late LBK, though the size of sites was smaller; no villages on the scale of Bischoffsheim in the LBK, for example, have been found so far. Hamlets of two or three contemporary farmsteads may be a better model (Leprovost 2012; Denaire 2014). So the recolonisation of Lower Alsace in the Hinkelstein and Grossgartach phases was not the same as at the start of the LBK sequence. It was more limited, and the first steps involved few sites; it took two or three generations to take in the whole of the area previously occupied in the LBK. The points of origin seem different too, with the Hinkelstein style deriving from the middle Rhine; people might also have come from the Neckar valley in the Grossgartach phase (Denaire 2009, pp. 338–40; 2012). Considerable contemporary variation in the decorative motifs used on pottery can now be seen. Was this the same kind of scenario again as discussed above for the LBK, with material culture signalling new identities, or did conditions of stability foster both innovation and wider regional contacts? This is presumably the kind of direction in which new explanation of contemporaneity of decoration must go. That the now diverse motifs are found in the same areas and on the same sites, but not in the same pits or graves (taking these as closed assemblages), is compatible with intense local differentiation in this phase. This interpretation will require fresh appraisal and further critical examination.

The Planig-Friedberg style represents only a little more development, and was a relatively short transitional phase (Denaire 2009; Denaire and Mauvilly 2012), of about a generation or so, between Grossgartach and Rössen. From large-scale excavations of settlements and cemeteries, we can see that sites started in the Grossgartach phase continue through Planig-Friedberg to the Rössen phase. The small number of Planig-Friedberg pits and graves does not suggest any further increase in settlement density, and the area of inhabitation does not expand (Denaire 2009, p. 266). Links to other regions also seem stable. A Planig-Friedberg pot in one of the graves at Passy in the Yonne in the south of the Paris basin (Spatz 1998) illustrates the extent of connections at this time.

Although the Rössen phase, over four to five generations, lasted longer than Grossgartach, it is less visible in Lower Alsace, given the 30 sites known, because of changes in the nature of features. Big pits, so common in the Grossgartach, were no longer dug, and the small pits of settlements are harder to detect and although some cemeteries remain in use, no substantial new one was started in the Rössen phase. There was, however, an expansion of the areas inhabited, for example north of the forest of Haguenau (Denaire 2009, pp. 267–8); on the other side of the Rhine, there was also expansion, towards the south of the plain of Baden, and in Upper Alsace, towards the north of Franche-Comté, seen in finds from the Cravanche cave (Denaire 2009, pp. 267–8). There was even more considerable expansion into central Germany (Spatz 1996, pp. 463–9). Was this the result of population increase or cultural integration?

The Bischheim and Bruebach-Oberbergen phases each lasted some three or four generations, and were shorter than the Rössen phase. Settlements are still defined by concentrations of various small pits, and postholes have been lost to erosion; there were no substantial cemeteries, only small clusters of graves (Denaire and Lefranc 2014). Such sites were often located at a remove from previous occupations, making their discovery more a matter of chance. The expansion of settlement continued, including notably, outside Alsace, in the direction of the Paris basin, where a western Bischheim pottery style has been recognised (Jeunesse et al. 2003).

Regionalisation of material culture became more marked in the course of the Bruebach-Oberbergen phase, represented across Upper and Lower Alsace as well as in the Neckar valley (Jeunesse et al. 2003), once again underlining the links with the latter. Elsewhere in the Rhine valley, the Bischheim style continued to develop. In Alsace, the river Zorn, to the north of Kochersberg, represents the boundary between Bruebach-Oberbergen and Bischheim. Such territoriality did not put an end to contacts with regions further afield, seen not only in the extension of the Bischheim style into the Paris basin and of the Bruebach-Oberbergen style into the Neckar valley (Jeunesse et al. 2003), but also in the import of decorated Bischheim and Bruebach-Oberbergen pots into Egolzwil contexts on the Swiss plateau (Denaire et al. 2011). The Bischheim pots in question appear to come from the Neckar valley, while the Bruebach-Oberbergen ones could be from Alsace.

At the end of the forty-third century cal BC, Lower Alsace saw the appearance of the BORS style, whose decoration seems to represent a fusion between the techniques of the makers of western Bischheim pottery in the Paris basin and those of the Chasséen (with incised decoration), seen for example in the rows of buttons and in chequerboard motifs. BORS marks such a stylistic break with Bruebach-Oberbergen that it is tempting to think of incoming potters and even population, from the west. Such contacts with the west go back to Bruebach-Oberbergen, as seen in pit 2020 at Rosheim Rittergass, which contained both Bruebach-Oberbergen and western Bischheim pots (Lefranc et al. 2012).

More than 30 BORS sites are known in Alsace, spread over an area similar to that of the Rössen stage and extending over areas where both the Bischheim and Bruebach-Oberbergen styles occurred previously. BORS has a more concentrated distribution than previous styles, but extends into Upper Alsace, at the expense of sites of the late phase of Bruebach-Oberbergen. To the north, there are contacts with eastern Bischheim style. BORS 0 has so far only been recognised at the site of Entzheim in the region of Strasbourg. BORS I settlements are also represented by concentrations of pits, but can be larger than in the preceding two stages (Jeunesse et al. 2003). In BORS II, the number of pits declines (Denaire 2014), perhaps reflecting a change in the character of occupation or shorter spans of time. BORS mortuary practice also marks a break, especially in the replacement of supine by flexed burials.

Most pits attributed to the BORS II also contain numerous Michelsberg II pots (Lüning 1968: there are no MK I pots in Alsace), once again showing contact from the west (Jeunesse et al. 2003). The only radiocarbon date currently available for the Michelsberg II in Alsace, from a grave (ETH-36782, 5185 ± 40 BP; 4050–3940 cal BC: Stuiver and Reimer 1986; Denaire and Mauduit 2010, p. 15), is compatible with the date estimate for the end of BORS II presented here.

### Mind the Gap: Models for LBK Crisis and General Models for Collapse and Regeneration

In the long sequence for Lower Alsace discussed above, it seems clear that there is a gap between the end of the LBK and the start of the Hinkelstein phase (Fig. 24). It is also probable that other regions up and down the Rhine valley and its catchment may also have seen a gap between LBK and Hinkelstein (Fig. 25). This deserves further consideration in its own right, since it may speak to the major issue of the conditions in which the LBK as a whole ended, and challenges the default assumption of continuity stressed at the start of this paper.

Still pertinent suggestions about the end of the LBK, involving disruption and climate change, were made by earlier generations of researchers (e.g. Meier-Arendt 1975, 155; Quitta 1969). With the subsequent discoveries of dramatic finds suggesting either violence or unusual mortuary rites, or both, at sites like Talheim, Asparn/Schletz, Herxheim and others (Wahl and König 1987; Teschler-Nicola et al. 1997; Boulestin et al. 2009; Meyer et al. 2014, 2015), these possibilities have coalesced into more general models of a crisis at the end of the LBK, leading to its decline and disappearance (Spatz 2002; Farruggia 2002). These have taken several forms, and as a generalisation, often seem to concentrate solely on the end of the LBK, neglecting the aftermath or transition to the post-LBK or Middle Neolithic situation. Some researchers have drawn attention to the possibility for high population growth by the late LBK, even if people were still concentrated in particular and relatively restricted parts of the landscape (Zimmermann et al. 2009). That potential has been part of a wider view of ‘boom and bust’ in Neolithic societies as a whole, though in this case using the density of radiocarbon dates as a proxy for population levels (Shennan et al. 2013), which can be disputed on many levels. Another approach has been multi-agent modelling, which applied to the development and end of the LBK has sought to investigate the links between climate variations, population increase, and possible ‘social dislocation caused by a short-term environmental crisis producing famine, with subsequent collapse’ (Bocquet-Appel et al. 2014, pp. 55 and 65). Yet another perspective has mooted the possibility of some kind of mental, conceptual or even moral crisis in late LBK society, at least in its western distribution (Zeeb-Lanz 2009; see Bánffy et al. 2016 for perspectives from Hungary and Link 2014 for arguments supporting continuity in the LBK’s eastern distribution).

Threats to resilience are a thread running through many of these kinds of model. Following earlier papers in which a much simpler climatic determinism was advocated (Gronenborn 2007, 2010, 2012), Detlef Gronenborn and colleagues have recently modelled the end of the LBK in more complex terms (Gronenborn et al. 2014). According to this view, ‘the simple farming societies of the LBK (5600–4900 cal BC) in west-central Europe were not immediately and devastatingly affected by most climate fluctuations’ (Gronenborn et al. 2014, p. 73). It is claimed, however, that the late LBK of west-central Europe saw a general trend to less rainfall, ‘punctuated by short-term increases in precipitation’; during what is argued to be a ‘climatically highly volatile period’, it is proposed that ‘LBK reaches its population rates and at the same time experiences a period of warfare. Thereafter population rates decline and LBK *gradually* vanishes from the archaeological record, being replaced by Middle Neolithic societies’ (Gronenborn et al. 2014, p. 73; our emphasis).

We do not seek here to dispute the possibility of a general link between climate-induced stress and socio-cultural change. We do want to draw attention to the chronological dimensions of what is being proposed under this banner. First, the span of the LBK has been informally estimated (‘5600–4900 cal BC’) as potentially longer than was the case in reality. Secondly, it remains very difficult precisely to correlate the various social, cultural, environmental and climatic trends, together with potential regional variations and differences. Thirdly, we should draw attention to the seemingly innocent adverb ‘gradually’. Much depends, as ever, on the timing of events.

It should also be noted that other researchers have been sceptical of the notion of late LBK crisis in general (Petrasch 1999; Stäuble 2014). Resilience, buffering and adaptive choices could all be summoned to counter-act notions of general crisis, within a general model of cyclical and gradual cultural change, nor should the impact of research traditions and fashions be overlooked (Stäuble 2014). Nonetheless, it seems fair to observe that much of the discussion of a potential late LBK crisis, whatever its possible causes, has been conducted either by concentrating on the late LBK itself or on the assumption that life somehow subsequently returned to normal: in both cases, neglecting potential aftermath. The Lower Alsace and wider Rhine valley models which we have provided here may suggest the possibility both of rapid decline and a measurable gap or hiatus, before the resumption of activity in the later forty-ninth and forty-eighth centuries cal BC.

In this kind of scenario, it is difficult not to think of bad things happening. Our paper cannot offer specific reasons for these, but it is useful briefly to note some of the possibilities that have been proposed in the wider—and vast—literature on collapse. One authoritative review (Tainter 1988, 2006) has underlined the complexity of collapse processes. Perhaps unsurprisingly, population ‘overshoot’, environmental change and degradation, agricultural failure, hostile neighbours and internal social conditions have all been proposed as reasons for collapse. Many assertions of one or other specific cause prove very hard, in many case studies, to document in detail. There are also differences of opinion on the likelihood of gradual process or rapid change due to extreme events. In the comparatively well documented case of twelfth century AD settlement of the central Mesa Verde region in south-west Colorado, it seems likely that high levels of population encountered intensifying drought, with conditions of instability, competition for resources, violence and final collapse coming to a head between 1250 and 1280 AD (Schwindt et al. 2016). From another perspective, constant threat of severe problems can foster ‘a culture of disaster’ (Bankoff 2002), so it is important not to underestimate the extent to which societies can buffer themselves, take avoiding action or make alternative choices.

One factor less discussed is disease, though major epidemics have been noted as among favourite explanations for catastrophic endings (Tainter 1988, p. 52). The evidence we have reviewed and modelled in this paper could suggest a significant swathe of west-central Europe—up and down the Rhine valley—with very little visible human activity in it for a century or so. Either there was outward migration, for unknown reasons, or such a shift in human practice as to change archaeological visibility for three or four generations, or there were genuinely very few people on the ground. If, hypothetically, the latter were the case, then some kind of epidemic might still have to be considered. We certainly know of the devastating impact of alien infectious disease, for example among the Iroquois in the mid-seventeenth century AD (Trigger 1976). There is comparative evidence to suggest that social disruption following epidemic disease increases the biological impact of an epidemic (McGrath 1991). The rapid spread of an infectious disease can be hard to trace in the skeletal record, but DNA and microbial analysis (e.g. of dental calculus) may yet shed light on the disease load of prehistoric populations (Warinner et al. 2015). But we are still left at this stage looking for possible signs of epidemic disease in LBK populations. There are indications that tuberculosis was present in the LBK population in Hungary, Slovakia and Austria, and a pulmonary infection has been identified in an individual at Ensisheim, Upper Alsace (Fibiger in Bickle and Whittle 2013, pp. 370–2). Tuberculosis, an endemic, rather than epidemic disease, is not usually a quick killer (Roberts and Buikstra 2003). Still, the history of this disease, in which the prevalence has risen and fallen alongside changes in population density, with the growing urbanisation of later Medieval Europe and the Industrial Revolution bringing the disease to epidemic levels, perhaps remains a useful analogy. In Lower Alsace, site numbers suggest that the region was most densely occupied during phase IV. Could this relative increase in density have created the conditions in which an epidemic took hold, leading to a significant decrease in population in phase V, followed by eventual abandonment? Advances in aDNA analysis hold the promise for the future detection of significant diseases and their mutations through time (Rasmussen et al. 2015).

Finally, the case for agency—for human choice—can be made not only in response to threat and disaster, but also in post-collapse regeneration (Schwarz 2013). If there was major disruption of some kind up and down the Rhine valley at the turn of sixth millennium cal BC, this does not seem to have been the case, in our view, everywhere: neither to the west in the Paris basin nor to the east in the Carpathian basin, though that would take several other papers to argue in detail. So life continued elsewhere, and eventually decisions were taken to re-inhabit lands putatively previously abandoned. Those taking such decisions were part of networks of practice within the still enduring Danubian tradition, and in Lower Alsace at least, the re-establishment of ways of doing things may have been quite rapid (*1–40 years* at *68% probability; duration start HK;* distribution not shown).

## Conclusions

In addressing the ‘cultural project’ in one small part of Europe, we have stressed the need to take meticulous care with each step in the process of formal modelling. We have presented critical readings of existing radiocarbon samples, seriations and chrono-typologies, provided a revised seriation for the Middle Neolithic, examined the possibility of diet-induced age offsets, and obtained 115 new radiocarbon dates on carefully chosen samples of known taphonomy. These have been combined with 37 existing radiocarbon measurements to produce a series of formal chronological models for the long sequence of cultural change, using pottery as the principal means of investigation, from the later sixth to the late fifth millennium cal BC in Lower Alsace, in the upper Rhine valley. We urge the necessity of the same kind of methodology for other comparable studies elsewhere.

The combination of seriation and typology with new radiocarbon dates in a Bayesian interpretive framework has produced powerful outcomes. The result has been a significantly finer-grained picture of the character and pace of cultural diversity and change. Rather than just recite a chronicle of pots, our approach has opened up a very detailed narrative of human agency, and we have been able to track the relative trajectories of material change and the intensity of settlement, mostly at a human generational timescale. Throughout the long sequence, things rarely seem to have stayed the same for long. That observation includes two major surprises, namely the inferred gap between the end of the LBK and the start of the Hinkelstein phase, and the apparent contemporaneity of diverse Grossgartach decorative schemes. Both are controversial, since they challenge existing understanding of the rate and nature of change. All the steps in the long sequence, however, seem to us to speak to a much more dynamic story, of stops and starts, repeated changes, successive disruptions and one major hiatus, than has hitherto been appreciated. We believe that this has significant implications for how we view cultural change elsewhere, both in Neolithic Europe and beyond, and for how we approach the human agency involved in the production and use of material culture.

## Electronic Supplementary Material

ESM 1 Sorted and phased matrix of the correspondence analysis of LBK ceramics in Lower Alsace. (GIF 389 kb)

High-resolution image (TIFF 1015 kb)

ESM 2 Sorted and phased matrix of the correspondence analysis of Middle Neolithic (Hinkelstein–Rössen) ceramics in Lower Alsace. (GIF 834 kb)

High-resolution image (TIFF 1941 kb)

ESM 3 Sorted and phased matrix of the correspondence analysis of Middle Neolithic (BORS) ceramics in Lower Alsace. (GIF 152 kb)

High-resolution image (TIFF 632 kb)

ESM 4 (DOC 32 kb)

ESM 5 (OXCAL 4 kb)

ESM 6 (OXCAL 4 kb)

ESM 7 (OXCAL 2 kb)

ESM 8 (OXCAL 10 kb)

ESM 9 (OXCAL 6 kb)

## Footnotes

1 We note the mass burials from Schöneck-Kilianstädten, Hesse, with four associated radiocarbon dates (MAMS-14991-4) (Meyer et al. 2014, 2015, Table S1). Full details of its associated pottery are not yet available (Detlef Gronenborn, *pers. comm*.). On the assumption that the associated ceramics are of comparable latest LBK style, we have added these dates to the model for the final LBK outside Lower Alsace (model not shown, but of the form of that defined in Fig. 23). The inclusion of this new data changes the median of the estimated date for the end of the final LBK in this area by 3 calendar years.

2 The negative part of this range reflects the probability (*9%*) that the use of LBK and Hinklestein ceramics overlapped in this area.

## References

[CR1] Adams WY, Adams EW (2008). Archaeological typology and practical reality: a dialectical approach to artifact classification and sorting.

[CR2] Bánffy, E., Osztás, A., Oross, K., Zalai-Gaál, I., Marton, T., Nyerges, É.Á., Köhler, K., Bayliss, A., Hamilton, D., & Whittle, A. (2016). The Alsónyék story: towards the history of a persistent place. *Bericht der Römisch-Germanischen Kommission, 94 (2013), 283–318*.

[CR3] Bankoff G (2002). Cultures of disaster: society and natural hazard in the Philippines.

[CR4] Barta P, Stolc S (2007). HBCO correction: its impact on archaeological absolute dating. Radiocarbon.

[CR5] Barth F, Barth F (1969). Introduction. Ethnic groups and boundaries: the social organisation of cultural difference.

[CR6] Baxter M (1994). Exploratory multivariate analysis in archaeology.

[CR7] Bayliss A (2009). Rolling out revolution: using radiocarbon dating in archaeology. Radiocarbon.

[CR8] Bayliss A, Bronk Ramsey C, van der Plicht J, Whittle A (2007). Bradshaw and Bayes: towards a timetable for the Neolithic. Cambridge Journal of Archaeology.

[CR9] Bayliss A, Hines J, Høilund Nielsen K, McCormac FG, Scull C (2013). Anglo-Saxon graves and grave goods of the sixth and seventh centuries AD: a chronological framework.

[CR10] Bayliss, A., van der Plicht, J., Bronk Ramsey, C., McCormac, G., Healy, F., & Whittle, A. (2011). Towards generational timescales: the quantitative interpretation of archaeological chronologies. In A. Whittle, F. Healy, & A. Bayliss (Eds.), *Gathering time: dating the early Neolithic enclosures of southern Britain and Ireland* (pp. 17–59). Oxford: Oxbow Books.

[CR11] Beavan, N., Mays, S., Bayliss, A., Hines, J., & McCormac, F. G. (2011). *Amino acid and stable isotope analysis of skeletons dated for the Anglo-Saxon chronology project*. Research Department Report Series, 88/2011.

[CR12] Bickle P, Whittle A (2013). The first farmers of Central Europe: diversity in LBK lifeways.

[CR13] Biel J (1987). Abschließende Untersuchung eines Michelsberger Erdwerks bei Heilbronn-Klingenberg. Archäologische Ausgrabungen in Baden-Württemberg.

[CR14] Biermann E (1997). Grossgartach und Oberlauterbach: interregionale Beziehungen im süddeutschen Mittelneolithikum.

[CR15] Bocquet-Appel, J.-P., Dubouloz, J., Moussa, R., Berger, J.-F., Tresset, A., Ortu, E., et al. (2014). Multi-agent modeling of the trajectory of the LBK Neolithic: a study in progress. In A. Whittle & P. Bickle (Eds.), *Early farmers: the view from archaeology and science* (pp. 53–69). Oxford: Oxford University Press for the British Academy.

[CR16] Boelicke U, Von Brandt D, Lüning J, Stehli P, Zimmermann A (1988). Der bandkeramische Siedlungsplatz Langweiler 8, Gemeinde Aldenhoven, Kreis Düren.

[CR17] Boës E, Jeunesse C, Arbogast R-M, Lefranc P, Mauvilly M, Schneikert F, Sidera I, Besse M (2007). Vendenheim-Le Haut du Coteau (Bas-Rhin): remarques sur l’organisation interne d’une nécropole du Néolithique ancien danubien. Sociétés néolithiques: des faits archéologiques aux fonctionnements socio-économiques.

[CR18] Bonnardin S (2009). La parure funéraire du Néolithique ancien en Bassins parisien et rhénan. Rubané, Hinkelstein, Villeneuve-Saint-Germain.

[CR19] Bonsall, C., Vasić, R., Boroneanţ, A., Roksandić, M., Soficaru, A., McSweeney, K., et al. (2015). New AMS ^14^C dates for human remains from stone age sites in the iron gates reach of the Danube, south-east Europe. *Radiocarbon, 57*, 33–46.

[CR20] Boulestin B, Zeeb-Lanz A, Jeunesse C, Haack F, Arbogast R-M, Denaire A (2009). Mass cannibalism in the linear pottery culture at Herxheim (Palatinate, Germany). Antiquity.

[CR21] Bronk Ramsey C (1995). Radiocarbon calibration and analysis of stratigraphy: the OxCal program. Radiocarbon.

[CR22] Bronk Ramsey C (2001). Development of the radiocarbon calibration program. Radiocarbon.

[CR23] Bronk Ramsey C (2009). Bayesian analysis of radiocarbon dates. Radiocarbon.

[CR24] Bronk Ramsey C (2009). Dealing with outliers and offsets in radiocarbon dating. Radiocarbon.

[CR25] Bronk Ramsey C, Lee S (2013). Recent and planned developments of the program OxCal. Radiocarbon.

[CR26] Buck CE, Litton CD, Smith AFM (1992). Calibration of radiocarbon results pertaining to related archaeological events. Journal of Archaeological Science.

[CR27] Buck CE, Cavanagh WG, Litton CD (1996). Bayesian approach to interpreting archaeological data.

[CR28] Buttler G (1935). Ein Hinkelsteingefäß aus Köln-Lindenthal und seine Bedeutung für die Chronologie der rheinischen Bandkeramik. Germania.

[CR29] Cahen D (1984). Organisation du village rubané de Darion (province de Liège, Belgique). Bulletin de la Société Royale Belge d’Anthropologie et de Préhistoire.

[CR30] Carrithers M (1992). Why humans have cultures: explaining anthropology and social diversity.

[CR31] Carrithers M (2010). Ontology is just another word for culture: for the motion (1). Critique of Anthropology.

[CR32] Croutsch, C., Rousselet O., & Tegel, W. (2013). Dambach-la-Ville (Bas-Rhin): un village de la fin du Ve millénaire. Résultats préliminaires de la fouille de la Plateforme d’Activités d’Alsace Centrale. In *31e Colloque Internéo 2013. Occupations et exploitations néolithiques: et si on parlait des plateaux? Pré-Actes. Internéo*, 45–6.

[CR33] Croutsch C, Denaire A, Ferrier A, Pelissier A, Rousselet O, Arbogast RM (2014). Obernai Schulbach/Nouvel Hôpital (Bas-Rhin, Alsace): puits et structures domestiques du Néolithique moyen. Internéo.

[CR34] Denaire, A. (2009). *Le Néolithique moyen du sud de la plaine du Rhin supérieur et du nord de la Franche-Comté: les cultures de Hinkelstein, Grossgartach et Rössen au travers de leur production céramique*. Strasbourg: Université de Strasbourg.

[CR35] Denaire, A. (2011). Chronologie absolue de la séquence Hinkelstein-Grossgartach-Roessen-Bischheim dans le sud de la plaine du Rhin supérieur et le nord de la Franche-Comté à la lumière des dernières données. In A. Denaire, C. Jeunesse, & P. Lefranc, (Eds.). *Nécropoles et enceintes danubiennes du 5e millénaire dans le nord-est de la France et le sud-ouest de l’Allemagne. Actes de la table ronde de Strasbourg, juin 2010, Monographie d’Archéologie du Grand Est,**5,* 9–30.

[CR36] Denaire, A. (2012). La place du sud du Rhin supérieur dans la première moitié du 5e millénaire av. J.-C. / Die Stellung des südlichen Oberrheins in der ersten Hälfte des V. Jahrtausends vor Christus. In R. Gleser, & V. Becker (Eds.). *Mitteleuropa im 5. Jahrtausend vor Christus* (pp. 207–227). Münster: Neolithikum und ältere Metallzeiten, Studien und Materialien 1.

[CR37] Denaire A (2013). Kolbsheim “Vogeseblick” du village du Néolithique ancien à la position de la Bruche de 1914.

[CR38] Denaire, A. (2014). L’habitat Grossgartach d’Ittenheim « Complexe sportif et aire de jeux de la rue de l’Érable » (Bas-Rhin). *Cahiers Alsaciens d’Arts, d’Archéologie et d’Histoire, 57*, 5–18.

[CR39] Denaire, A., & Lefranc, P. (2014). Les pratiques funéraires de la culture de Roessen et des groupes épiroesséniens dans le sud de la plaine du Rhin supérieur (4750–4000 av. J.-C.). In P. Lefranc, A. Denaire, & C. Jeunesse (Eds.), *Données récentes sur les pratiques funéraires néolithiques de la Plaine du Rhin supérieur,* (pp. 75–126). Oxford: Archaeopress.

[CR40] Denaire, A., & Mauduit, A. (2010). Matzenheim « Le Lavoir » (Bas-Rhin): un nouvel habitat du Néolithique ancien et une tombe du Michelsberg ancien du Rhin supérieur. *Cahiers Alsaciens d’Archéologie, d’Arts et d’Histoire, 53*, 3–19.

[CR41] Denaire, A., & Mauvilly, M. (2012). Guémar « Rotenberger Weg », premiére grande nécropole Grossgartach et Roessen (Néolithique moyen) de Haute-Alsace. *Internéo, 9*, 73–85.

[CR42] Denaire A, Doppler T, Nicod P-Y, Van Willigen S (2011). Espaces culturels, frontières et interactions au 5e millénaire entre la Plaine du Rhin Supérieur et les rivages de la Méditerranée. Annuaire d’Archéologie Suisse.

[CR43] Denaire A, Chenal F, Jammet-Reynal L (2014). Schwindratzheim “Les Terrasses de la Zorn” (Bas-Rhin): céramique de Limbourg, habitat rubané, sépultures et enceinte Bischheim. Internéo.

[CR44] Durrwachter C, Craig OE, Collins MJ, Burger J, Alt KW (2006). Beyond the grave: variability in Neolithic diets in southern Germany?. Journal of Archaeological Science.

[CR45] Eisenhauer U (2002). Untersuchungen zur Siedlungs- und Kulturgeschichte des Mittelneolithikums in der Wetterau.

[CR46] Evin J, Marien G, Pachiaudi C (1976). Lyon national radiocarbon measurements I. Radiocarbon.

[CR47] Evin J, Marien G, Pachiaudi C (1979). Lyon national radiocarbon measurements VIII. Radiocarbon.

[CR48] Fabian J (1983). Time and the other: how anthropology makes its object.

[CR49] Farruggia J-P (2002). Une crise majeure de la civilisation du Néolithique danubien des années 5100 avant notre ère. Archeologické rozhledy.

[CR50] Farrugia JP, Kuper R, Lüning J, Stehli P (1973). Der bandkeramische Siedlungsplatz Langweiler 2. Gemeinde Aldenhoven, Kreis Düren.

[CR51] Fernandes R, Millard AR, Brabec M, Nadeau M-J, Grootes P (2014). Food reconstruction using isotopic transferred signals (FRUITS): a Bayesian model for diet reconstruction. PloS One.

[CR52] Fildes, V. A. (1986). *Breasts, bottles and babies: a history of infant feeding*. Edinburgh: Edinburgh University Press.

[CR53] Forrer, R. (1911a). Das Neolithische Gräberfeld bei Lingolsheim. *Anzeiger für Elsässische Altertumskunde, 9–10*, 149–171.

[CR54] Forrer R (1911). Das Neolithische Gräberfeld bei Lingolsheim. Anzeiger für Elsässische Altertumskunde.

[CR55] Forrer R (1912). Das Neolithische Gräberfeld bei Lingolsheim. Anzeiger für Elsässische Altertumskunde.

[CR56] Forrer, R. (1922). Nouvelles découvertes et acquisitions du musée préhistorique et gallo-romain de Strasbourg. *Anzeiger für Elsässische Altertumskunde, 49–52*, 1–34.

[CR57] Forrer R (1938). Le cimetière de Lingolsheim à poteries poinçonnées, au crâne trépané et aux tombes de la zone rubanée. Cahiers Alsaciens d’Archéologie, d’Art et d’Histoire.

[CR58] Fritsch B (1998). Die linearbandkeramische Siedlung Hilzingen “Forsterbahnried” und die altneolitische Besiedlung des Hegaus.

[CR59] Fuller BT, Fuller JL, Harris DA, Hedges REM (2006). Detection of breastfeeding and weaning in modern human infants with carbon and nitrogen stable isotope ratios. American Journal of Physical Anthropology.

[CR60] Greenacre M (1984). Theory and applications of correspondence analysis.

[CR61] Greenacre M (1993). Correspondence analysis in practice.

[CR62] Gronenborn D, Pollard T, Banks I (2007). Climate change and sociopolitical crises: some cases from Neolithic Central Europe. War and sacrifice: studies in the archaeology of conflict.

[CR63] Gronenborn D, Gronenborn D, Petrasch J (2010). Climate, crise and the “neolithisation” of Central Europe between IRD events 6 and 4. Die Neolithisierung Mitteleuropas I (the spread of the Neolithic to Central Europe).

[CR64] Gronenborn, D. (2012). Das Ende von IRD 5b: Abrupte Klimafluktuationen um 5100 den BC und der Übergang vom Alt- zum Mittelneolithikum im westlich Mitteleuropa. In S. Wolfram, & H. Stäuble (Eds.). *Siedlungsstruktur und Kulturwandel in der Bandkeramik* I, (pp. 241–50). Dresden: Arbeits- und Forschungsberichte zur sächsischen Bodenkmalpflege.

[CR65] Gronenborn D, Strien H, Dietrich S, Sirocko F (2014). ‘Adaptive cycles’ and climate fluctuations: a case study from linear pottery culture in western Central Europe. Journal of Archaeological Science.

[CR66] Häusser A (2000). Ausgrabungen in einer jüngstbandkeramischen Siedlung in Herxheim b. L., Rheinland-Pfalz — Vorberichte. Varia Neolithica.

[CR67] Hedges REM, Clement JG, Thomas CDL, O’Connell TC (2007). Collagen turnover in the adult femoral mid-shaft: modelled from anthropogenic radiocarbon tracer measurements. American Journal of Physical Anthropology.

[CR68] Howcroft R, Eriksson G, Liden K (2012). The Milky Way: the implications of using animal milk products in infant feeding. Anthropozoologica.

[CR69] Ingold T, Ingold T (1996). Against the motion (1). Key debates in anthropology.

[CR70] Jay M, Fuller BT, Richards MP, Knüsel CJ, King SS (2008). Iron age breastfeeding practices in Britain: isotopic evidence from Wetwang Slack, East Yorkshire. American Journal of Physical Anthropology.

[CR71] Jeunesse C (1995). Contribution à l’étude de la variabilité régionale au sein du Rubané. L’exemple du Sud de la plaine du Rhin supérieur. Cahiers de l’Association pour la promotion de la recherche archéologique en Alsace.

[CR72] Jeunesse C (1997). Pratiques funéraires au Néolithique ancien. Sépultures et nécropoles danubiennes 5500–4900 av. J.-C.

[CR73] Jeunesse C (1999). La synchronisation des séquences culturelles des bassins du Rhin, de la Meuse et de la Seine et la chronologie du Bassin parisien au Néolithique ancien et moyen (5200-4500 av. J. C.*)*. Bulletin de la Société Préhistorique Luxembourgeoise.

[CR74] Jeunesse C. (2008). Variations stylistiques et formation des groupes régionaux dans le Rubané occidental. L’exemple des décors orthogonaux. In F. Falkenstein, S. Schade-Lindig, & A. Zeeb-Lanz (Eds.). *Kumpf, Kalotte, Pfeilschaftglätter. Zwei Leben für die Archäologie. Gedenkschrift für Annemarie Häusser und Helmut Spatz*, (pp. 129–151). Rahden: Marie Leidorf.

[CR75] Jeunesse C, Schnitzler B (2009). Vendenheim: une nécropole du Néolithique ancien. 10 000 ans d’histoire! Dix ans de fouilles archéologiques en Alsace: Catalogue d’exposition.

[CR76] Jeunesse C, Arbogast R-M (1997). L’habitat Néolithique moyen (cultures de Grossgartach et de Roessen) de Rosheim «Mittelweg» & «Sandgrube» (Bas-Rhin) dans le cadre du Néolithique moyen du sud de la Plaine du Rhin supérieur. Deuxième partie: étude archéozoologique et synthèse générale. Cahiers de l’Association pour la Promotion de la Recherche Archéologique en Alsace.

[CR77] Jeunesse, C., & Lefranc, P. (1999). Rosheim “Sainte-Odile” (Bas-Rhin), un habitat rubané avec fossé d’enceinte — Première partie: les structures et la céramique. *Cahiers de l’Association pour la Promotion de la Recherche Archéologique en Alsace, 15*, 1–111.

[CR78] Jeunesse, C., Lefranc, P., & Denaire, A. (2003). *Groupe de Bischheim, origine du Michelsberg, genèse du groupe d’Entzheim. La transition entre le Néolithique moyen et le Néolithique récent dans les régions rhénanes*. Strasbourg: Cahiers de l’Association pour la Promotion de la Recherche Archéologique en Alsace 18/19.

[CR79] Joachim W (1993). Ein kleiner mittelneolithischer Bestattungsplatz der Hinkelstein-Kultur in Remseck-Aldingen, Kr. Ludwigsburg. Archäologische Ausgrabungen in Baden-Württemberg.

[CR80] Jones M, Nicholls G (2001). Reservoir offset models for radiocarbon calibration. Radiocarbon.

[CR81] Kalis AJ, Zimmermann A (1988). Wirkungen neolithischer Wirtschaftsweisen in Pollendiagrammen. Archäologischer Informationen.

[CR82] Karlsberg, A. J. (2006)*. Flexible Bayesian Methods for Archaeological Dating*. Unpublished PhD thesis, University of Sheffield, UK.

[CR83] Katzenberg MA, Saunders SR, Fitzgerald WR (1993). Age differences in stable carbon and nitrogen isotope ratios in a population of prehistoric maize horticulturists. American Journal of Physical Anthropology.

[CR84] Katzenberg, M. A., Harring, D. A., & Saunders, S. R. (1996). Weaning and infant mortality: evaluating the skeletal evidence. *Yearbook of Physical Anthropology, 38*, 177–199.

[CR85] Keaveney EM, Reimer PJ (2012). Understanding the variability in freshwater radiocarbon offsets. Journal of Archaeological Science.

[CR86] Knauft BM (1993). South coast new Guinea cultures: history, comparison, dialectic.

[CR87] Kromer B, Manning SW, Friedrich M, Talamo S, Trano N (2010). ^14^C calibration in the 2nd and 1st millennia BC—Eastern Mediterranean radiocarbon comparison project (EMRCP). Radiocarbon.

[CR88] Kuper A (1999). Culture: the anthropologists’ account.

[CR89] Lanting JN, van der Plicht J (1998). Reservoir effects and apparent ^14^C ages. Journal of Irish Archaeology.

[CR90] Lee S, Bronk Ramsey C (2012). Development and application of the trapezoidal model for archaeological chronologies. Radiocarbon.

[CR91] Lefranc, P. (2006). *Les groupes régionaux du Rubané et la colonisation du sud de la Plaine du Rhin supérieur. Actes du XIVème Congrès de l’UISPP, Liège. (pp. 9–17)*. Oxford: Archaeopress.

[CR92] Lefranc P (2007). La céramique du Rubané en Alsace.

[CR93] Lefranc, P. (2011). Deux enceintes de type “Rosheim” de la seconde moitié du 5e millénaire à Entzheim “Les Terres de la Chapelle” et Duntzenheim “Frauenabwand” (Bas-Rhin). Premiers résultats. In A. Denaire, C. Jeunesse, & P. Lefranc (Eds.). *Nécropoles et enceintes danubiennes du 5e millénaire dans le Nord-Est de la France et le Sud-Ouest de l’Allemagne* (pp. 85–102). Strasbourg: Monographie d’Archéologie du Grand Est.

[CR94] Lefranc P (2012). Entzheim “Les Terres de la Chapelle”.

[CR95] Lefranc P (2013). Les relations entre les groupes rubanés d’Alsace et du bassin de la Seine: l’apport des styles céramique. Bulletin de la Société Préhistorique Française.

[CR96] Lefranc P, Bakaj B, Robert F, Zehner M (2004). Bischoffsheim “AFUA du Stade”.

[CR97] Lefranc P, Serrurier A, Michler M (2012). Un ensemble mixte Bruebach-Oberbergen/Bischheim occidental sur le site de Rosheim « Rittergass » (Bas-Rhin): premiers impacts occidentaux sur le sud de la plaine du Rhin supérieur. Revue Archéologique de l’Est.

[CR98] Lefranc P, Alix G, Latron F, Lefranc P, Denaire A, Jeunesse C (2014). La nécropole Rubané récent d’Ingenheim « Bannenberg » (Bas-Rhin). Données récentes sur les pratiques funéraires néolithiques de la Plaine du Rhin supérieur.

[CR99] Lefranc P, Chenal F, Cicutta H, Gebhardt A, Guthmann E, Thomas Y, Véber C (2015). Vendenheim (Bas-Rhin), Aux portes du Kochersberg. Enceinte, habitats et systèmes de fentes néolithiques (Roessen, Bruebach-Oberbergen et Michelsberg) et camps d’entrainement romain.

[CR100] Lefranc, P., Denaire, A., & Boës, E. (2010). L’habitat néolithique ancien et moyen d'Ittenheim (Bas-Rhin). *Revue Archéologique de l'Est, 59*, 65–97.

[CR101] Leprovost C (2012). La fouille preventive de la plateforme départementale d’activités de Brumath et environs: découverte du premier village du Néolithique moyen en Alsace. Internéo.

[CR102] Leprovost, C., & Queyras, M. (2011). La nécropole d’Entzheim (Bas-Rhin): nouvelles données sur le Néolithique moyen alsacien. In A. Denaire, C. Jeunesse, & P. Lefranc (Eds.), *Nécropoles et enceintes danubiennes du Ve millénaire dans le Nord-Est de la France et le Sud-Ouest de l’Allemagne* (pp. 115–125). Strasbourg: Université de Strasbourg.

[CR103] Lichardus-Itten M (1980). Die Gräberfelder der Grossgartach Gruppe im Elsass.

[CR104] Link T (2014). Die linien- und stichbandkeramische Siedlung von Dresden-Prohlis. Eine Fallstudie zum Kulturwandel in der Region der oberen Elbe um 5000 v. Chr.

[CR105] Lüning J (1968). Die Michelsberg Kultur. Ihre Funde in zeitliche und räumliche Gliederung. Berichte der Römisch-Germanisch Kommission.

[CR106] Lönne P (2003). Das Mittelneolithikum im südlichen Niedersachsen: Untersuchungen zum Kulturenkomplex Grossgartach – Planig-Friedberg – Rössen und zur Stichbandkeramik.

[CR107] Mays S, Beavan N (2012). An investigation of diet in early Anglo-Saxon England using carbon and nitrogen stable isotope analysis of human bone collagen. Journal Archaeological Science.

[CR108] McCormac, F.G., Thompson, M., & Brown, D. (2001). Characterisation, optimisation and standard measurements for two small-sample high-precision radiocarbon counters. *Centre for Archaeol Rep*, 8/2001.

[CR109] McCormac FG, Bayliss A, Baillie MGL, Brown DM (2004). Radiocarbon calibration in the Anglo-Saxon period: AD 495–725. Radiocarbon.

[CR110] McCormac FG, Bayliss A, Brown DM, Reimer PJ, Thompson MM (2008). Extended radiocarbon calibration in the Anglo-Saxon period, AD 395–485 and AD 735–805. Radiocarbon.

[CR111] McCormac, F.G., Reimer, P.J., Bayliss, A., Thompson, M.M., Beavan, N., Brown, D., & Hoper, S.T. (2011). *Laboratory and Quality Assurance Procedures at the Queen’s University, Belfast Radiocarbon Dating Laboratory for Samples dated for the Anglo-Saxon Chronology Project*. Research Department Report Series, 89/2011.

[CR112] McGrath JW (1991). Biological impact of social disruption resulting from epidemic disease. American Journal of Physical Anthropology.

[CR113] Meier-Arendt W (1966). Die bandkeramische Kultur in Untermaingebiet.

[CR114] Meier-Arendt W (1975). Die Hinkelstein Gruppe.

[CR115] Meunier K, Sidera I, Arbogast R-M (2003). Rubané et groupe d'Entzheim à Pfuhlgriesheim “Langgarten” et “Buetzel” (Bas-Rhin). Bulletin de la Société Préhistorique Française.

[CR116] Meyer, C., Lohr, C., Gronenborn, D., & Alt, K.W. (2015). The massacre mass grave of Schöneck-Kilianstädten reveals new insights into collective violence in Early Neolithic central Europe. *Proceedings of the National Academy of Sciences*. doi: 10.1073/pnas.1504365112. 10.1073/pnas.1504365112 PMC4568710 26283359

[CR117] Meyer C, Lohr C, Kürbis O, Dresely V, Haak W, Adler CJ, Gronenborn D, Whittle A, Bickle P (2014). Mass graves of the LBK: patterns and peculiarities. Early farmers: the view from archaeology and science.

[CR118] Molleson T (1994). The eloquent bones of Abu Hureyra. Scientific American.

[CR119] O’Connell TC, Kneale CJ, Tasevska N, Kuhnle GGC (2012). The diet–body offset in human nitrogen isotopic values: a controlled dietary study. American Journal of Physical Anthropology.

[CR120] Perrin, B. (2011). L’enceinte à pseudo-fossé Roessen de Meistratzheim (Bas-Rhin). In A. Denaire, C. Jeunesse and P. Lefranc (eds), *Nécropoles et enceintes danubiennes du 5e millénaire dans le Nord-Est de la France et le Sud-Ouest de l’Allemagne*, (pp. 73–84). Strasbourg: Monographie d’Archéologie du Grand Est.

[CR121] Perrin, B. (2013). *Osthouse “Kleinfeld”*. Unpublished final excavation report, Strasbourg.

[CR122] Petrasch J (1999). Mord und Krieg in der Bandkeramik. Archäologisches Korrespondenzblatt.

[CR123] Quitta, H. (1964). Zur Herkunft des frühen Neolithikums in Mitteleuropa. In P. Grimm (Ed.), *Varia Archaeologica: Wilhelm Unverzagt zum 70. Geburtstag dargebracht* (pp. 14–24). Berlin: Deutsche Akademie der Wissenschaften zu Berlin.

[CR124] Quitta, H. (1969). Zur Deutung bandkeramischer Siedlungsfunde aus Aue und grunwassernahen Standorten. In K.H. Otto, & J. Herrman (Eds.). *Siedlung, Burg und Stadt*, (pp. 42–55). Berlin: Deutsche Akademie der Wissenschaften zu Berlin, Schriften der Sektion für Vor- und Frühgeschichte.

[CR125] Rasmussen, S., Allentoft, M. K., Nielsen, K., Orlando, L., Sikora, M., Sjogren, K.-G., et al. (2015). Early divergent strains of *Yersinia pestis* in Eurasia 5,000 years ago. *Cell, 163*, 571–582. 10.1016/j.cell.2015.10.009 PMC4644222 26496604

[CR126] Reimer, P. J., Bard, E., Bayliss, A., Beck, J. W., Blackwell, P., Bronk Ramsey, C., et al. (2013). IntCal13 and Marine13 radiocarbon age calibration curves 0–50,000 years cal BP. *Radiocarbon, 55*, 1869–1887.

[CR127] Roberts CA, Buikstra JE (2003). The bioarchaeology of tuberculosis: a global view on a re-emerging disease.

[CR128] Schier W, Schier W, Draşovean F (2014). The copper age in Southeast Europe—historical epoch or typo-chronological construct?. The Neolithic and Eneolithic in Southeast Europe: new approaches to dating and cultural dynamics in the 6th and 4th millennium BC.

[CR129] Schwarz KR (2013). Through the rearview mirror: rethinking the classic Maya collapse in the light of Postclassic rural social transformation. Journal of Social Archaeology.

[CR130] Schwindt DM, Bocinsky RK, Ortman SG, Glowacki DM, Varien MD, Kohler TA (2016). The social consequences of climate change in the central mesa Verde region. American Antiquity.

[CR131] Seidel, U., Stephan, E., Stika, H.-P., Dunbar, E., Kromer, B., Bayliss, A., Beavan, N., Healy, F., & Whittle, A. (2016). Die Zeit der großen Gräben: Modelle zur Chronologie des Michelsberger Fundplatzes von Heilbronn-Klingenberg “Schlossberg”, Stadtkreis Heilbronn, Baden-Württemberg. *Praehistorische Zeitschrift, 91*(2), 225–283.

[CR132] Seifert M, Boschetti-Maradi A, de Capitani A, Hachuli S, Niffeler U (2012). Zizers GR-Friedau—mittelneolithische Siedlung mit Hinkelsteinkeramik in Bünder Alpenrheintal (Schweiz). Form, Zeit und Raum: Grundlagen für Werner E. Stöckli zu seinem 65. Geburtstag.

[CR133] Seifert MT, Sormaz T, Stöckli EW, de Capitani A (2013). Die absolute Datierung von Egolzwil 3. Egolzwil 3. Der Keramik der neolithischen Seeufersuedlung.

[CR134] Shennan, S., Downey S. S., Timpson A., Edinborough K., Colledge S., Kerig T., Manning K., & Thomas M. G. (2013). Regional population collapse followed initial agriculture booms in mid-Holocene Europe. *Nature Communications,* 4, www.nature.com/ncomms/2013/131001/ncomms3486/full/ncomms3486.html 10.1038/ncomms3486 PMC3806351 24084891

[CR135] Song R (2004). Reconstructing infant diet and weaning behaviour of ancient Maya from Lamanai, Belize, using laser ablation-inductively coupled plasma-mass Spectometry (LA-ICP-MS).

[CR136] Spatz, H. (1996). *Beiträge zum Kulturkomplex Hinkelstein — Grossgartach — Rössen: der keramische Fundstoff des Mittelneolithikums aus dem mittleren Neckarland und seine zeitliche Gliederung*. Stuttgart: Materialhefte zur Archäologie in Baden-Württemberg.

[CR137] Spatz H (1998). Le vase de Passy sur Yonne: attribution chronologique – synchronisation. Bulletin de la Société Préhistorique Française.

[CR138] Spatz, H. (1999). *Das mittelneolithische Gräberfeld von Trebur, Kreis Groß-Gerau*. Wiesbaden: Materialien zur Vor- und Frühgeschichte von Hessen.

[CR139] Spatz H (2001). Zur Verlässlichkeit von Knochendatierungen—das Beispiel Trebur, Südhessen. Praehistoria Alpina.

[CR140] Spatz, H. (2002). Bäumchen und Sichel: Aspekte und Überlegungen zum Übergang vom frühen zum mittleren Neolithikum in Zentraleuropa. *Archeologické Rozhledy LIV*, 279–300.

[CR141] Stadler B, Wirth K (2006). Edingen-Neckarhausen, Rhein-Neckar-Kreis—eine archäologische Fundgrube am Neckar. Archäologische Ausgrabungen in Baden-Wurttemberg.

[CR142] Stäuble H, Link T, Schimmelpfennig D (2014). Die Krise am Ende der Linienbandkeramik? Oder ist es am Ende eine Krise der Bandkeramik-Forschung?! Ein archäologisches Feuilleton. No future? Brüche und Ende kultureller Erscheinungen. Beispiele aus dem 6.–2. Jahrtausend v. Chr.

[CR143] Strien H-C (2000). Untersuchungen zur Bandkeramik in Württemberg.

[CR144] Strien H-C, Gronenborn D, Gronenborn D (2005). Klima- und Kulturwandel während des mitteleuropäischen Altneolithikums (58./57.–51./50. Jahrhundert v. Chr.). Klimaveränderung und Kulturwandel in neolithischen Gesellschaften Mitteleuropas, 6700–2200 v. Chr.

[CR145] Stuiver M, Polach HA (1977). Reporting of ^14^C data. Radiocarbon.

[CR146] Stuiver M, Reimer PJ (1986). A computer program for radiocarbon age calculation. Radiocarbon.

[CR147] Stuiver, M., & Reimer, P. J. (1993). Extended 14C data base and revised CALIB 3.0 14C age calibration program. *Radiocarbon, 35*, 215–230.

[CR148] Styring A, Maier U, Stephan E, Bogaard A (2016). Cultivation of choice: new insights into farming practices at Neolithic lakeshore sites. Antiquity.

[CR149] Tainter JA (1988). The collapse of complex societies.

[CR150] Tainter JA (2006). Archaeology of overshoot and collapse. Annual Review of Anthropology.

[CR151] Taylor RE, Southon J (2013). Reviewing the mid-first millennium BC ^14^C “warp” using ^14^C/bristlecone pine data. Nuclear Instruments and Methods in Physics Research B.

[CR152] Teschler-Nicola M, Gerold F, Kranz K, Lindenbauer K, Spannagel M (1997). Anthropologische Spurensicherung. Die traumatischen und postmortalen Veränderungen an den linearbandkeramischen Skelettresten von Asparn/Schletz. Archäologie in Österreich.

[CR153] Trigger BG (1976). The children of Aataentsic: a history of the Huron people to 1660.

[CR154] Tromme F (1982). Campagnes 1980 et 1981 au site Omalien “Fond Chenai” à Awans. Notae Praehistoricae.

[CR155] Wahl J, König HG (1987). Anthropologisch-Traumatologische Untersuchung der menschlichen Skelettreste aus dem bandkeramischen Massengrab bei Talheim. Kr. Heilbronn. Fundberichte aus Baden-Württemberg.

[CR156] Ward, G. K., & Wilson, S. R. (1978). Procedures for comparing and combining radiocarbon age determinations: a critique. *Archaeometry, 20*, 19–31.

[CR157] Warinner C, Speller C, Collins MJ (2015). A new era in palaeomicrobiology: prospects for ancient dental calculus as a long-term record of the human oral microbiome. Philosophical Transactions of the Royal Society of London Series B, Biological Sciences.

[CR158] White CD, Schwarcz HP (1994). Temporal trends in stable isotopes for Nubian mummy tissues. American Journal of Physical Anthropology.

[CR159] Wild, E. M., Stadler, P., Häußer, A., Kutschera, W., Steier, P., Teschler-Nicola, M., et al. (2004). Neolithic massacres: local skirmishes or general warfare in Europe? *Radiocarbon, 46*, 377–385.

[CR160] Wotzka HP, Laufer E, Posselt M, Starossek B (2001). “Peripherie” Plätze der späten Bandkeramik im Usinger Becken (ostlicher Hintertaunus, Hessen). Vorbericht für die Jahre 1999 und 2000. Berichte der Kommission für Archäologische Landesforschung in Hessen.

[CR161] Zeeb-Lanz A, Zeeb-Lanz A (2009). Gewaltszenarien oder Sinnkrise? Die Grubenanlage von Herxheim und das Ende der Bandkeramik. Krisen—Kulturwandel—Kontinuitäten: zum Ende der Bandkeramik in Mitteleuropa.

[CR162] Zimmermann A, Wendt KP, Frank T, Hilpert J (2009). Landscape archaeology in Central Europe. Proceedings of the Prehistoric Society.

