# Supplementary figures and images for: The Cultural Project: Formal Chronological Modelling of the Early and Middle Neolithic Sequence in Lower Alsace
Source: J Archaeol Method Theory. 2017 Jan 9;24(4):1072–149. doi: 10.1007/s10816-016-9307-x (PMC5732602; doi:10.1007/s10816-016-9307-x)

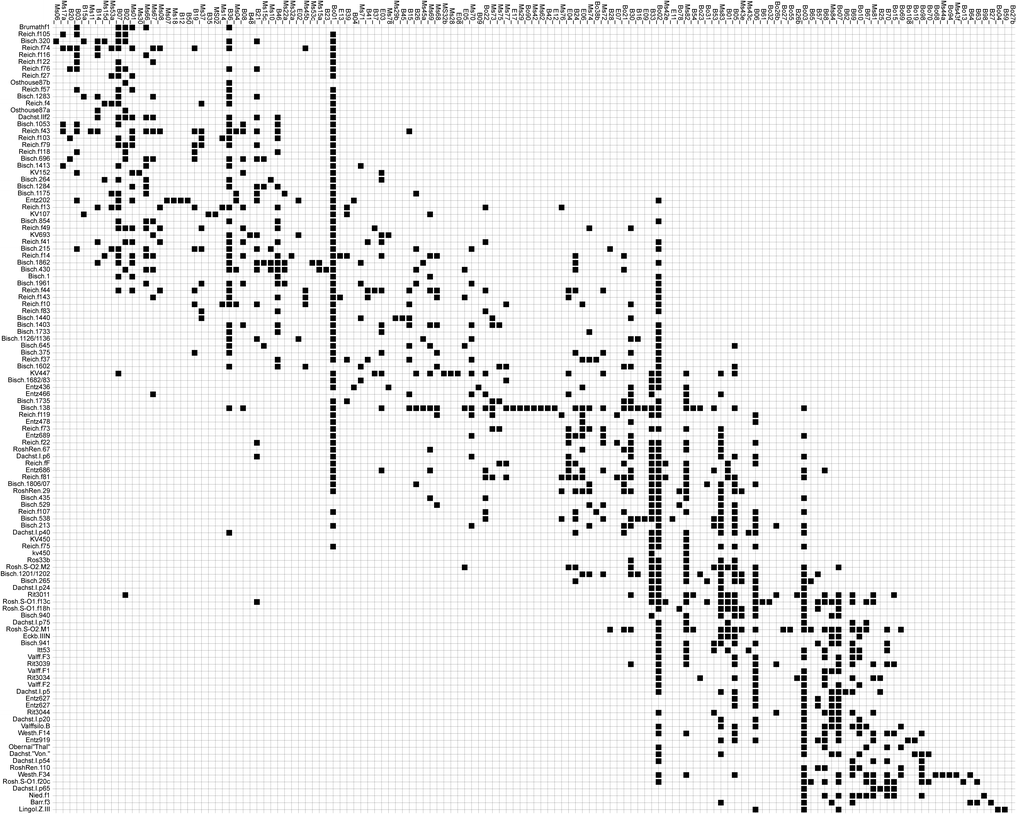

Supplement: Supplementary file 1 — Sorted and phased matrix of the correspondence analysis of LBK ceramics in Lower Alsace. (GIF 389 kb) [file 10816_2016_9307_Fig26_ESM.gif]

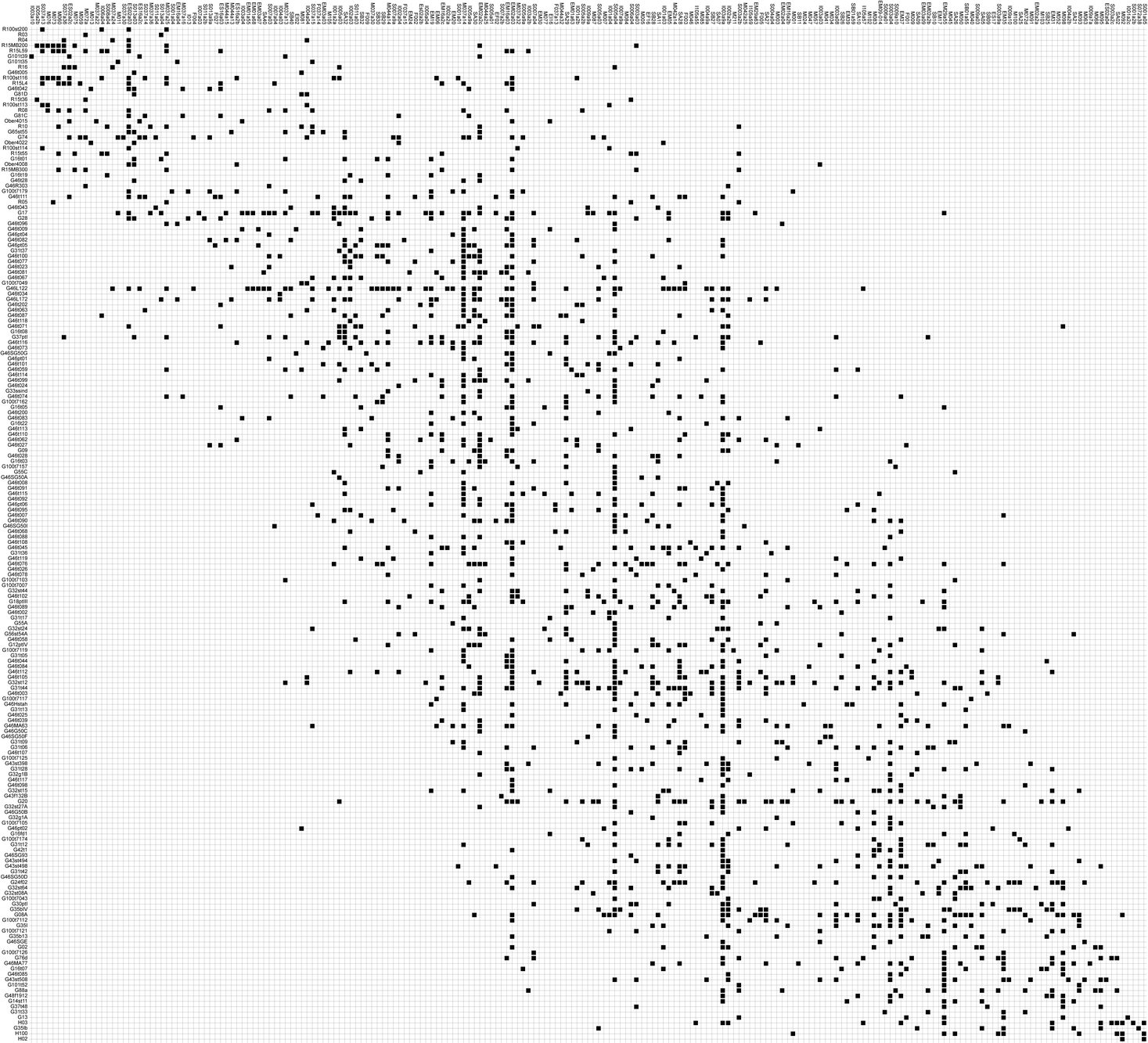

Supplement: Supplementary file 3 — Sorted and phased matrix of the correspondence analysis of Middle Neolithic (Hinkelstein–Rössen) ceramics in Lower Alsace. (GIF 834 kb) [file 10816_2016_9307_Fig27_ESM.gif]

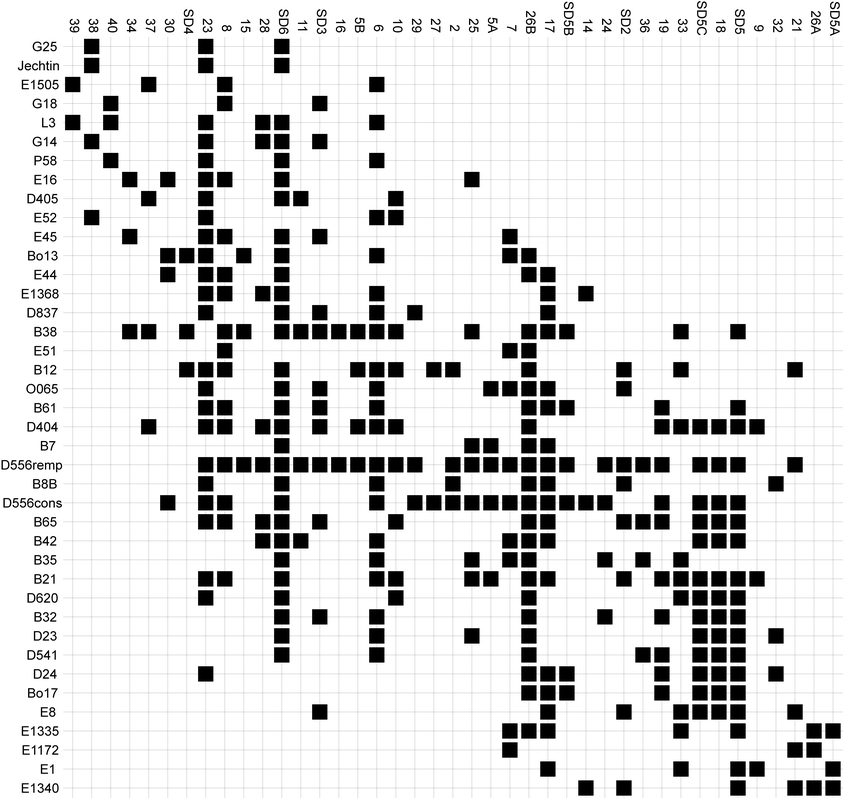

Supplement: Supplementary file 5 — Sorted and phased matrix of the correspondence analysis of Middle Neolithic (BORS) ceramics in Lower Alsace. (GIF 152 kb) [file 10816_2016_9307_Fig28_ESM.gif]
