# Supplementary material for: The Cultural Project: Formal Chronological Modelling of the Early and Middle Neolithic Sequence in Lower Alsace
Source: J Archaeol Method Theory. 2017 Jan 9;24(4):1072–149. doi: 10.1007/s10816-016-9307-x (PMC5732602; doi:10.1007/s10816-016-9307-x)
Supplement: Supplementary file 7 — (DOC 32 kb) [file 10816_2016_9307_MOESM4_ESM.doc]

Supplementary Information: Laboratory Methods

The stable isotopic values in Tables 1 and 2 were measured by Isotope Ratio Mass Spectrometry (IRMS) on sub-samples of the carbon dioxide combusted during the dating process. At the Scottish Environmental Research and Reactor Centre (SUERC) these values were used for age calculation; at the other laboratories fractionation correction was made using δ^13^C values measured by Accelerator Mass Spectrometry (AMS) during the dating process.

Sixty bone samples were dated at SUERC, East Kilbride, Scotland (SUERC-). They were processed by gelatinisation and ultrafiltration (Brock *et al.* 2010), combusted to carbon dioxide (Vandeputte *et al.* 1996), graphitised (Slota *et al.* 1987), and dated by AMS (Freeman *et al.* 2010). Stable isotopic ratios were determined as described by Sayle *et al.* (2014). Forty-seven samples of bone and six carbonised residues on pottery sherds were dated by the Oxford Radiocarbon Accelerator Unit (OxA-). Bones were prepared using gelatinisation and ultrafiltration and the residues were given an acid wash, with OxA-29695 and OxA-X-2555-56 undergoing an additional solvent extraction (Brock *et al* 2010). Samples were then combusted and graphitised (Dee and Bronk Ramsey 2000), and dated by AMS (Bronk Ramsey *et al.* 2004). Stable isotopic ratios were determined as described by Brock *et al.* (2010). Two animal bones were dated using AMS at the ^14^CHRONO Centre, The Queen’s University, Belfast (UBA-), using the revised bone gelatinisation protocol and zinc reduction graphitisation methods described by Reimer *et al.* (2015). A further 11 bone samples and two carbonised residues failed to produce sufficient material for dating in these laboratories.

As part of previous programmes of research, 24 samples of bone and six samples of carbonised plant remains had been dated by the Poznań Radiocarbon Laboratory (Poz-). Charcoal samples were pretreated as described by Mook and Waterbolk (1985) and the bone processed to gelatin and ultra-filtered (Brown *et al*. 1988), before they were combusted and graphitised (Czernik and Goslar 2001), and dated by AMS (Goslar *et al*. 2004). Two bone samples had been dated at the Rijksuniversiteit, Groningen (GrA-). These samples were gelatinised (Longin 1971), combusted to carbon dioxide and graphitised (Aerts-Bijma *et al*. 1997; 2001) and dated by AMS (van der Plicht *et al*. 2000). Three samples had been dated by the Lyon Radiocarbon Laboratory (Ly-) in the 1970s. Ly-865 was dated by gas proportional counting of carbon dioxide as described by Evin *et al*. (1969; 1973); and the other two measurements were made by liquid scintillation spectrometry as described by Evin *et al*. (1978; 1979).

**References**

Aerts-Bijma, A.T., Meijer, H.A.J. and van der Plicht, J. 1997. AMS sample handling in Groningen. *Nuclear Instruments and Methods in Physics Research B* 123, 221–5.

Aerts-Bijma, A.T, van der Plicht, J. and Meijer, H.A.J. 2001. Automatic AMS sample combustion and CO_2_ collection. *Radiocarbon 43*, 293–8.

Brock, F., Higham, T.F.G., Ditchfield, P. and Bronk Ramsey, C. 2010. Current pretreatment methods for AMS radiocarbon dating at the Oxford Radiocarbon Accelerator Unit (ORAU). *Radiocarbon* 52, 103–12.

Brown, T.A., Nelson, D.E., Vogel, J.S. and Southon, J.R. 1988. Improved collagen extraction by modified Longin method. *Radiocarbon* 30, 171–7.

Bronk Ramsey, C., Higham, T. and Leach, P. 2004. Towards high precision AMS: progress and limitations. *Radiocarbon* 46, 17–24.

Czernik, J. and Goslar, T. 2001. Preparation of graphite targets in the Gliwice Radiocarbon Laboratory for AMS^14^C dating. *Radiocarbon* 43, 283–91.

Dee, M. and Bronk Ramsey, C. 2000. Refinement of graphite target production at ORAU. *Nuclear Instruments and Methods in Physics Research B* 172, 449–53.

Evin, J., Marien, G. and Pachiaudi, C. 1969. Lyon National Radiocarbon Measurements I. *Radiocarbon* 11, 112–17.

Evin, J., Marien, G. and Pachiaudi, C. 1973 Lyon National Radiocarbon Measurements III. *Radiocarbon* 15, 134–55.

Evin, J., Marien, G. and Pachiaudi, C. 1978 Lyon National Radiocarbon Measurements VII. *Radiocarbon* 20, 19–57.

Evin, J., Marien, G. and Pachiaudi, C. 1979. Lyon National Radiocarbon Measurements VIII. *Radiocarbon* 21, 405–52.

Freeman, S.P.H.T, Cook, G.T., Dougans, A.B., Naysmith, P., Wilcken, K.M. and Xu, S. 2010. Improved SSAMS performance. *Nuclear Instruments and Methods in Physics Research Section B* 268, 715–17.

Goslar, T., Czernik, J. and Goslar, E. 2004. Low-energy ^14^C AMS in Poznan Radiocarbon Laboratory, Poland. *Nuclear Instruments and Methods in Physics Research B* ***223­–224****, 5–11.*

Longin, R. 1971. New method of collagen extraction for radiocarbon dating. *Nature* 230, 241–2.

Mook, W.G. and Waterbolk, H.T. 1985. *Radiocarbon dating.* Strasbourg: European Science Foundation*.*

Reimer, P.J, Hoper, S., McDonald, J., Reimer, R., Svyatko, S. and Thompson, M. 2015. *The Queen’s University, Belfast: laboratory protocols used for AMS radiocarbon dating at the ^14^CHRONO Centre. English Heritage Research Report 5.* Swindon: English Heritage.

Sayle, K.L., Cook, G.T., Ascough, P.L., Gestsdóttir, H., Hamilton W.D. and McGovern, T.H. 2014. Utilization of δ^13^C, δ^15^N, and δ^34^S analyses to understand ^14^C dating anomalies within a Late Viking Age community in Northeast Iceland. *Radiocarbon* 56, 811–21.

Slota, P.J. Jr, Jull, A.J.T., Linick, T.W. and Toolin, L.J. 1987. Preparation of small samples for ^14^C accelerator targets by catalytic reduction of CO. *Radiocarbon* 29, 303–6.

Vandeputte, K., Moens, L. and Dams, R. 1996. Improved sealed-tube combustion of organic samples to CO_2_ for stable isotope analysis, radiocarbon dating and percent carbon determinations. *Analytical Letters* 29, 2761–73.

van der Plicht, J., Wijma, S., Aerts, A.T., Pertuisot, M.H. and Meijer, H.A.J. 2000. Status report: the Groningen AMS facility. *Nuclear Instruments and Methods in Physics Research B* 172, 58–65.
